# Magnetic catalyst marvels: a sustainable approach to highly substituted imidazole synthesis

**DOI:** 10.1039/d5na00368g

**Published:** 2025-08-13

**Authors:** Mosstafa Kazemi, Ramin Javahershenas, Jayanti Makasana, Suhas Ballal, Munther Kadheem, Abhayveer Singh, Kattela Chennakesavulu, Kamal Kant Joshi

**Affiliations:** a Young Researchers and Elite Club, Tehran Branch, Islamic Azad University Tehran Iran mosstafakazemi@gmail.com; b Organic Development, Chemistry Faculty, Urmia University Urmia Iran jshbco@gmail.com jshbco@yahoo.com; c Marwadi University Research Center, Department of Chemistry, Faculty of Science, Marwadi University Rajkot-360003 Gujarat India jayantilal.makasana@marwadieducation.edu.in; d Department of Chemistry and Biochemistry, School of Sciences, JAIN (Deemed to be University) Bangalore Karnataka India sballalschem@gmail.com; e College of Pharmacy, the Islamic University Najaf Iraq; f College of Pharmacy, the Islamic University of Al Diwaniyah Al Diwaniyah Iraq muntherabosoda@iunajaf.edu.iq; g Centre for Research Impact & Outcome, Chitkara University Institute of Engineering and Technology, Chitkara University Rajpura Punjab 140401 India abhayveer_singh@outlook.com; h Department of Chemistry, Sathyabama Institute of Science and Technology Chennai Tamil Nadu India chennakesavulureddy.chemistry@sathyabama.ac.in; i Department of Allied Science, Graphic Era Hill University Dehradun India; j Graphic Era Deemed to be University Dehradun Uttarakhand India kkjoshi@gehu.ac.in

## Abstract

This comprehensive review delves into the recent advancements in magnetic catalyst technology, mainly focusing on their application in facilitating greener and more efficient synthetic routes for imidazole derivatives. This manuscript assesses various magnetic catalyst systems, examining their synthesis, functionalization, and mechanistic roles in promoting imidazole formation. Special attention is given to the environmental benefits of using magnetic catalysts, such as reduced solvent use, lower energy consumption, and enhanced recyclability, which align with sustainable chemistry principles. The unique properties of magnetic catalysts, including their easy recovery *via* external magnetic fields and reusability without significant loss of activity, are highlighted as key factors driving the synthetic processes' sustainability and economic viability. Furthermore, the review discusses the challenges and limitations currently faced in this realm and proposes future directions for research, including the development of novel magnetic catalyst compositions and the exploration of their utility in other heterocyclic syntheses. By providing a detailed analysis of existing data and suggesting pathways for innovation, this review aims to inspire continued advancement in sustainable catalysis, promising to revolutionize the synthesis of highly substituted imidazoles and expand their potential applications in various industries. This manuscript is a crucial resource for researchers in catalysis and sustainable chemistry. It underscores the broader implications of magnetic catalysts in enhancing green manufacturing practices in the chemical industry, thereby contributing to global sustainability goals.

## Abstract 2

This comprehensive review delves into the recent advancements in magnetic catalyst technology, mainly focusing on their application in facilitating greener and more efficient synthetic routes for imidazole derivatives.

## Graphical abstract

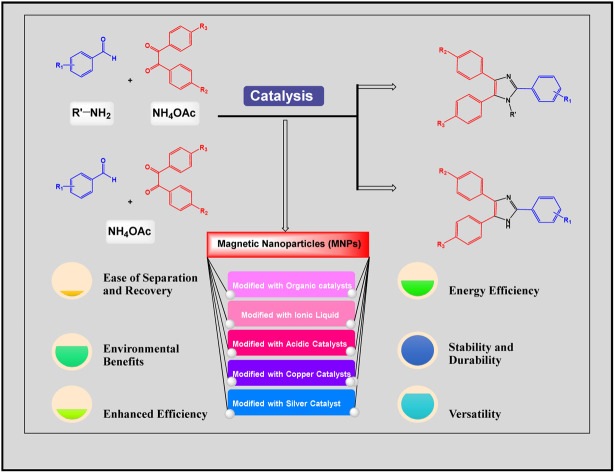

## 1. Introduction

### 1.1. Catalysts

Catalysts play a vital role in chemistry by accelerating reactions and lowering activation energies without being consumed, allowing for repeated use.^1,2^ Enzymes, as natural catalysts, reduce energy barriers, enhancing both the rate and thermodynamic feasibility of biochemical reactions.^3^ In industrial settings, catalysts improve efficiency by saving time and energy and guiding reactions selectively to increase desired products while reducing byproducts.^4^ About 90% of commercial chemical processes rely on catalysts to boost yields and drive innovation.^5^ They also enable reactions at lower temperatures and pressures, lowering costs and environmental impact,^6^ and are crucial in producing biodegradable plastics and essential pharmaceuticals.^7^ Moreover, catalysts support sustainable technologies, such as solar fuels, and help address environmental challenges through cleaner processes. An ideal catalyst is efficient, selective, durable, and recyclable—key traits for sustainable and innovative chemical transformations.^8^

**Fig. 1:**
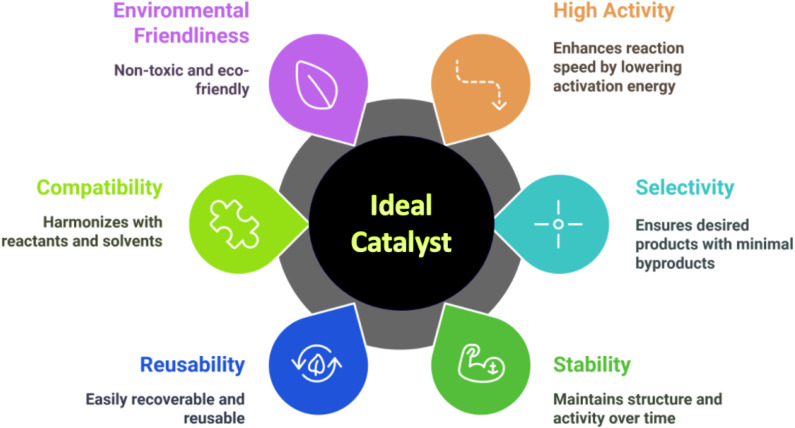
Advantageous of an ideal catalyst.

Advantageous of an ideal catalyst (Fig. 1):

✓ *High Activity*: it is expected to greatly enhance the reaction speed by reducing the activation energy, enabling the reaction to occur more rapidly and effectively.^9^

✓ *Selectivity*: a perfect catalyst ought to be extremely selective, facilitating the creation of the intended product while reducing the generation of undesired byproducts.^10^

✓ *Stability*: it should remain stable under the reaction conditions, preserving its activity and structure over time without undergoing degradation or deactivation.^11^

✓ *Reusability*: the catalyst needs to be easily recoverable and reusable without a substantial decrease in activity, which would enhance both the cost-effectiveness and sustainability of the process.^12^

✓ *Compatibility*: it needs to be compatible with the reactants and solvents involved in the reaction, making sure that it does not disrupt the entire process.^9^

✓ *Environmental friendliness*: an optimal catalyst ought to be non-toxic and environment friendly, aiding in the development of greener and more sustainable chemical processes.^13^

When selecting catalysts, it's important to consider their cost-effectiveness in both initial investments and ongoing operational expenses, particularly energy consumption. Catalysts are crucial for enhancing efficiency and reducing costs across various industrial processes. By optimizing production methods, they facilitate more sustainable and environmentally friendly manufacturing practices.^13^

### 1.2. Magnetically recoverable catalysts (MRCs)

Researchers have increasingly turned to nanocatalysts in chemical reactions, thanks to their outstanding efficiency and precision that can revolutionize the field.^13^ Nanocatalysts are incredibly small, with a large surface area compared to their volume. This characteristic allows them to have more active sites, enhancing the efficiency and speed of chemical reactions.^14^ It delivers faster reaction rates, boosts yields, conserves energy, and significantly reduces waste production.^15^ Furthermore, their remarkable ability to catalyze chemical reactions under milder conditions while providing greater accuracy has made them indispensable in a variety of fields. This includes crucial applications in the pharmaceutical industry, where precise drug development is vital, as well as in environmental cleanup efforts, where efficient and effective solutions are needed to address contamination challenges.^16^

By adopting the principles of green chemistry, magnetic catalysts offer a sustainable and eco-friendly method for chemical synthesis. They enhance efficiency while reducing harmful substances, paving the way for a cleaner, greener future in the chemical industry.^17,18^ Magnetic catalysts contribute to a more sustainable future by reducing waste, conserving energy, and minimizing the use of hazardous substances.^19,20^ The use of magnetic catalysts can enhance reaction rates and selectivity, resulting in more efficient chemical processes with reduced by-product formation.^21,22^ The use of magnetic catalysts in chemical synthesis represents a significant advancement in green chemistry. This innovative approach aims to reduce, and ideally eliminate, the use and production of hazardous substances, offering a safer and more sustainable alternative to traditional methods. By enhancing the efficiency of chemical reactions and allowing for easy recovery through magnetic manipulation, magnetic catalysts contribute to minimizing waste and resource consumption. This development is essential in the pursuit of eco-friendly solutions, promoting a cleaner environment and better public health.^23^

**Fig. 2:**
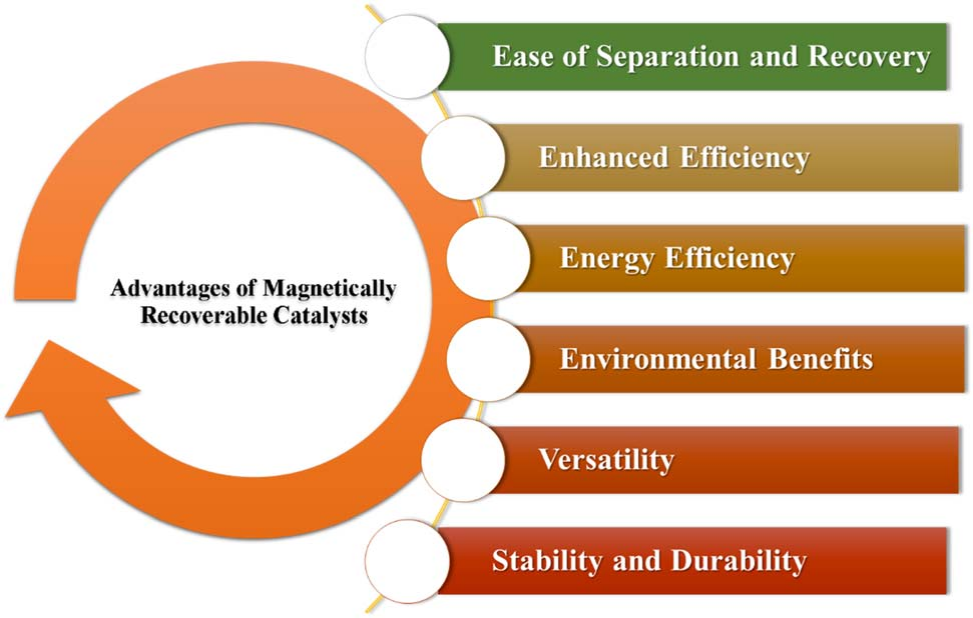
Advantages of Magnetically Recoverable Catalysts (MRCs).

Magnetic catalysts offer significant advantages in chemical reactions, positioning themselves as essential elements in the field of modern catalysis (Fig. 2). By harnessing their unique magnetic properties, these catalysts not only accelerate reaction rates but also improve selectivity, resulting in more favorable and efficient product outcomes. A standout feature of magnetic catalysts is their ease of separation from reaction mixtures; by applying a magnetic field, researchers can swiftly isolate the catalysts, simplifying the purification process and reducing waste. This blend of efficiency and user-friendly separation methods establishes magnetic catalysts as pivotal contributors to the ongoing advancement of contemporary chemical processes, fostering innovation and sustainability within the discipline.

Advantage of Magnetically Recoverable Catalysts (MRCs)

✓ *Ease of separation and recovery*: one of the primary benefits of magnetic catalysts is their ability to be easily separated from the reaction mixture using an external magnetic field. This simplifies the recovery and reuse of the catalyst, reducing costs and waste.^24^

✓ *Enhanced efficiency*: magnetic catalysts can improve the efficiency of chemical reactions. The magnetic properties allow for better dispersion of the catalyst in the reaction medium, ensuring more effective contact with reactants.^25^

✓ *Energy efficiency*: magnetic catalysts can be heated directly through electromagnetic induction, which is more energy-efficient than conventional heating methods. This localized heating reduces energy consumption and can lead to faster reaction rates.^26,27^

✓ *Environmental benefits*: the ability to recover and reuse magnetic catalysts minimizes waste and reduces the environmental impact of chemical processes.^28^ Additionally, the use of magnetic fields can eliminate the need for harmful solvents or additional purification steps.^29^

✓ *Versatility*: magnetic catalysts can be used in a wide range of reactions, including organic synthesis, environmental remediation, and industrial processes.^30^ They can be tailored to specific reactions by modifying their surface properties or combining them with other catalytic materials.^31^

✓ *Stability and durability*: many magnetic catalysts, especially those based on iron oxide, are stable under a variety of reaction conditions. This stability ensures that they maintain their catalytic activity over multiple cycles.^32^

✓ *Safety*: the use of magnetic fields for heating can be safer than traditional methods, as it reduces the risk of overheating and allows for precise control.^33^

#### 1.2.1. Bridge between homogeneous and heterogeneous catalysts

**Fig. 3:**
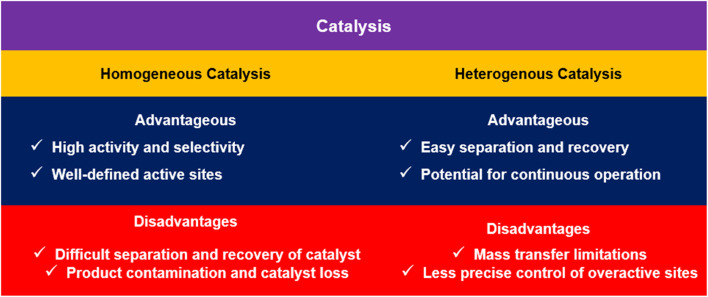
Advantages and disadvantages of homogeneous and heterogeneous catalysts.

Catalysts are crucial agents in chemical reactions, speeding up processes while remaining unchanged. They are typically divided into two primary categories: homogeneous catalysts, present in the same phase as the reactants, and heterogeneous catalysts, which exist in a different phase (Fig. 3). Homogeneous catalysts, in particular, are known for their reliable performance and selectivity, instilling confidence in their use. Interestingly, magnetic catalysts merge the characteristics of both types. Their magnetic properties facilitate easy manipulation and separation from reaction mixtures, enhancing the advantages of each type and significantly improving overall reaction efficiency.^34^ They take advantage of both kinds, with the selectivity of homogeneous catalysts and the straightforward separation and recovery associated with heterogeneous catalysts.^35,36^ This groundbreaking technique simplifies the recovery phase by eliminating the need for tedious filtration or centrifugation methods. As a result, the process becomes more efficient, significantly improving the overall effectiveness of chemical reactions while reducing the time and effort typically required for catalyst recovery.^37,38^ This process not only simplifies the retrieval and reuse of the catalysts, but it also minimizes waste and prevents contamination.^39^ Recent advancements in the realm of chemistry have unveiled an intriguing phenomenon: the ability of magnetic fields to influence the energy levels of active species involved in chemical reactions. This impact arises from the engagement with the spin states of these entities—core attributes associated with the angular momentum of subatomic particles. Scientists skillfully adjust these spin states to successfully direct the path of chemical reactions, leading them to the desired results and significantly improving reaction yields in previously thought impossible ways. This revolutionary insight paves the way for groundbreaking strategies to control chemical processes at a molecular level, offering new possibilities for innovation and discovery in the field.^40^ Subtle fluctuations in the magnetic field can significantly influence the quantum mechanical wavefunction of particles involved in a collision, causing their reaction rates to either accelerate or slow down.^41,42^ This innovative approach opens up possibilities for controlling reaction pathways and improving catalytic efficiency.^43,44^ Magnetic catalysts represent a significant breakthrough in the development of catalytic processes, combining enhanced efficiency with sustainability. By harnessing their unique magnetic properties, these catalysts not only accelerate chemical reaction rates but also allow for easier and more efficient separation from reaction mixtures. This simplified separation process minimizes waste generation and reduces overall energy consumption, leading to an eco-friendlier approach to chemical production. As a result, magnetic catalysts pave the way for innovative and greener methodologies in the industrial landscape, contributing to a healthier environment and more sustainable practices in chemical manufacturing.^45^ Magnetic catalysts combine the advantages of homogeneous and heterogeneous catalysts, providing improved efficiency and easier catalyst recovery.

**Fig. 4:**
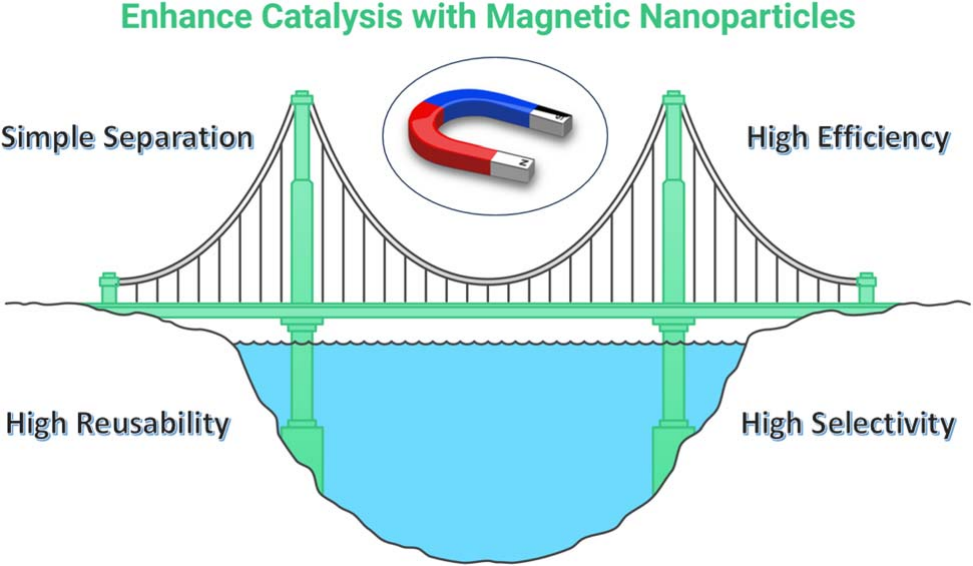
Magnetically recoverable catalysts (MRCs) as a bridge between homogeneous and heterogeneous catalysts.

Magnetically Recoverable Catalysts (MRCs) as a bridge:

Magnetic catalysts provide an answer to the constraints associated with both homogeneous and heterogeneous catalysts (Fig. 4):

➢ *Homogeneous-like activity*: magnetic nanoparticles can be modified with homogeneous catalysts, including organometallic complexes or enzymes.^46^ These catalysts maintain their strong activity and selectivity, akin to homogeneous catalysts.^47^

➢ *Heterogeneous-like separation*: the nanoparticles' magnetic properties facilitate simple removal from the reaction mixture with the aid of external magnetic fields.^48^ This streamlines the process of purifying products and facilitates the recovery and reuse of catalysts.

➢ *Improved mass transfer*: the tiny dimensions of magnetic nanoparticles improve the mass transfer between the catalyst and the reactants, leading to faster reaction rates.^49^

➢ *Controlled active sites*: the surface of magnetic nanoparticles can be meticulously designed to establish distinct active sites, resembling the functionality of homogeneous catalysts. This precise engineering enables the customization of catalytic properties, leading to enhanced selectivity and greater efficiency in various chemical reactions. Such tailored surfaces hold the promise of optimizing performance in catalytic processes, making them a valuable tool in various industrial applications.^50^

#### 1.2.2. Surface modification of magnetic nanoparticles

Surface modification of magnetic nanoparticles (MNPs) greatly enhances their functionality and compatibility for applications like catalysis and drug delivery. By tailoring the physical and chemical properties of their surfaces, this process improves interactions and boosts efficiency, leading to better stability, higher drug loading capacity, and increased catalytic activity. This versatility makes MNPs valuable tools in advanced technology.^51^ Organic catalysts, including small molecules and polymers, are crucial for modifying the surfaces of magnetic nanoparticles (MNPs). This modification prevents rapid agglomeration and oxidation, helping to maintain the nanoparticles' magnetic properties and overall effectiveness in various applications.^52,53^ These organic coatings can introduce particular functional groups that improve the surface's capacity to participate in further chemical reactions or to bond with other molecules. This ability can greatly enhance the material's characteristics and broaden its uses across different sectors.^54^ Transition metal catalysts improve the properties of metal nanoparticles (MNPs), resulting in highly effective heterogeneous catalysts for chemical transformations. Known for their excellent activity and selectivity, these catalysts enable efficient reactions that produce the desired products while minimizing byproducts, making them valuable in pharmaceuticals, materials science, and environmental chemistry.^55^ The surfaces of magnetic nanoparticles (MNPs) can be customized with catalytic metals like palladium or copper. This modification enhances their ability to drive complex chemical reactions efficiently while reducing undesired side reactions, leading to improved performance in various applications.^56^ Modified magnetic nanoparticles (MNPs) are effective catalysts due to their large surface area, which enhances reaction efficiency, and their magnetic properties, which allow for easy separation and recovery from mixtures. This makes them highly valuable in both industrial and laboratory applications.^57,58^ The expanded surface area of nanoparticles dramatically enhances the number of catalytic sites accessible for chemical reactions, which leads to significantly accelerated reaction rates. Moreover, these nanoparticles possess unique magnetic properties that allow for their easy separation from the reaction mixture when subjected to an external magnetic field. This feature not only simplifies the process of recovering and reusing the catalysts but also promotes greater sustainability and cost-effectiveness in operations. As a result, this innovative approach to catalysis presents a compelling solution for a wide range of applications in various chemical processes, highlighting its versatility and efficiency in industrial settings.^59^ This magnetic separation technique completely removes the necessity for filtration or centrifugation, guaranteeing minimal loss of the catalyst and efficiently avoiding product contamination.^60^ In the realm of organic synthesis, modified magnetic nanoparticles (MNPs) have emerged as a pivotal tool in a wide array of chemical reactions. These advanced particles possess unique properties that significantly enhance the formation of compounds recognized for their considerable biological and pharmaceutical importance. By facilitating more efficient synthesis processes, MNPs contribute to the production of high-purity products with exceptional yields. Their remarkable capabilities as catalysts in intricate chemical transformations not only highlight their effectiveness but also underscore their potential to revolutionize practices in the field, paving the way for innovative solutions and valuable new compounds.^61,62^ The innovative process of modifying the surface of magnetic nanoparticles (MNPs) with organic compounds and transition metal catalysts has opened up exciting new avenues in the realm of green chemistry. This significant advancement not only improves the catalytic properties of these nanoparticles but also facilitates the development of environmentally sustainable and highly efficient catalytic processes. By leveraging the unique characteristics and functional capabilities of these specially modified MNPs, researchers have the opportunity to design and implement chemical reactions that significantly reduce harmful environmental impacts while maximizing both performance and efficacy. The integration of these advanced materials into catalytic systems represents a pivotal step towards achieving more sustainable practices in chemical manufacturing and other industrial applications.^63^

### 1.3. Highly substituted imidazoles

Highly substituted imidazoles are remarkable heterocyclic compounds that possess a wide range of applications and significant importance across multiple fields. Their synthesis has garnered substantial interest among researchers, mainly due to the numerous benefits they offer in various contexts, including biological, medical, chemical, agricultural, and industrial sectors. The unique structural versatility of highly substituted imidazoles enables them to engage effectively with diverse biological targets, positioning them as valuable scaffolds in the realm of drug development. These compounds are well-known for their extensive array of biological and pharmacological activities, which have been documented in numerous studies. This makes highly substituted imidazole derivatives not only a focal point of scientific research but also critical components in the design of innovative therapeutic agents.

#### 1.3.1. Biological and pharmaceutical importance

**Fig. 5:**
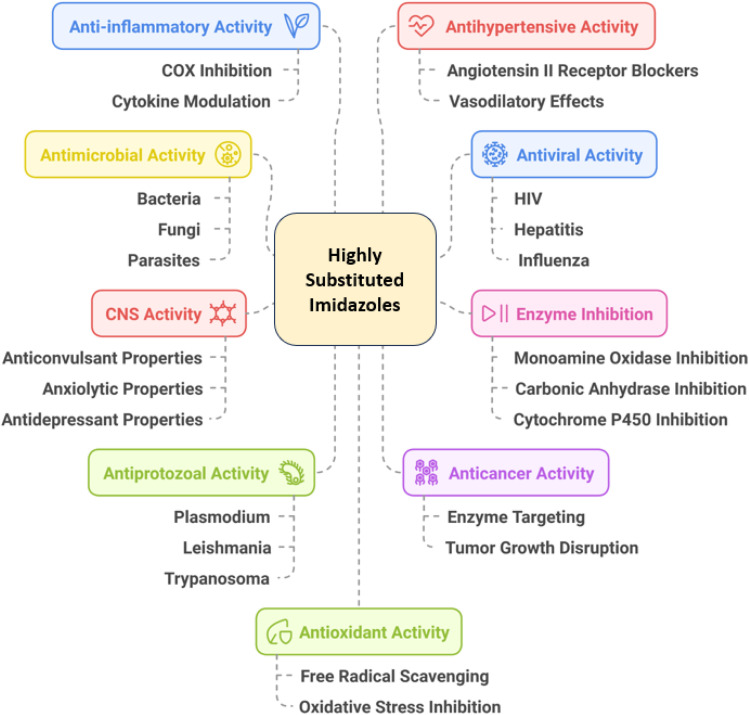
Biological and pharmacological activities of highly substituted imidazole derivatives.

Highly substituted imidazole derivatives are an important class of heterocyclic compounds with a wide range of biological and pharmacological activities.^64^ Here are several key activities associated with these compounds (Fig. 5).

**Fig. 6:**
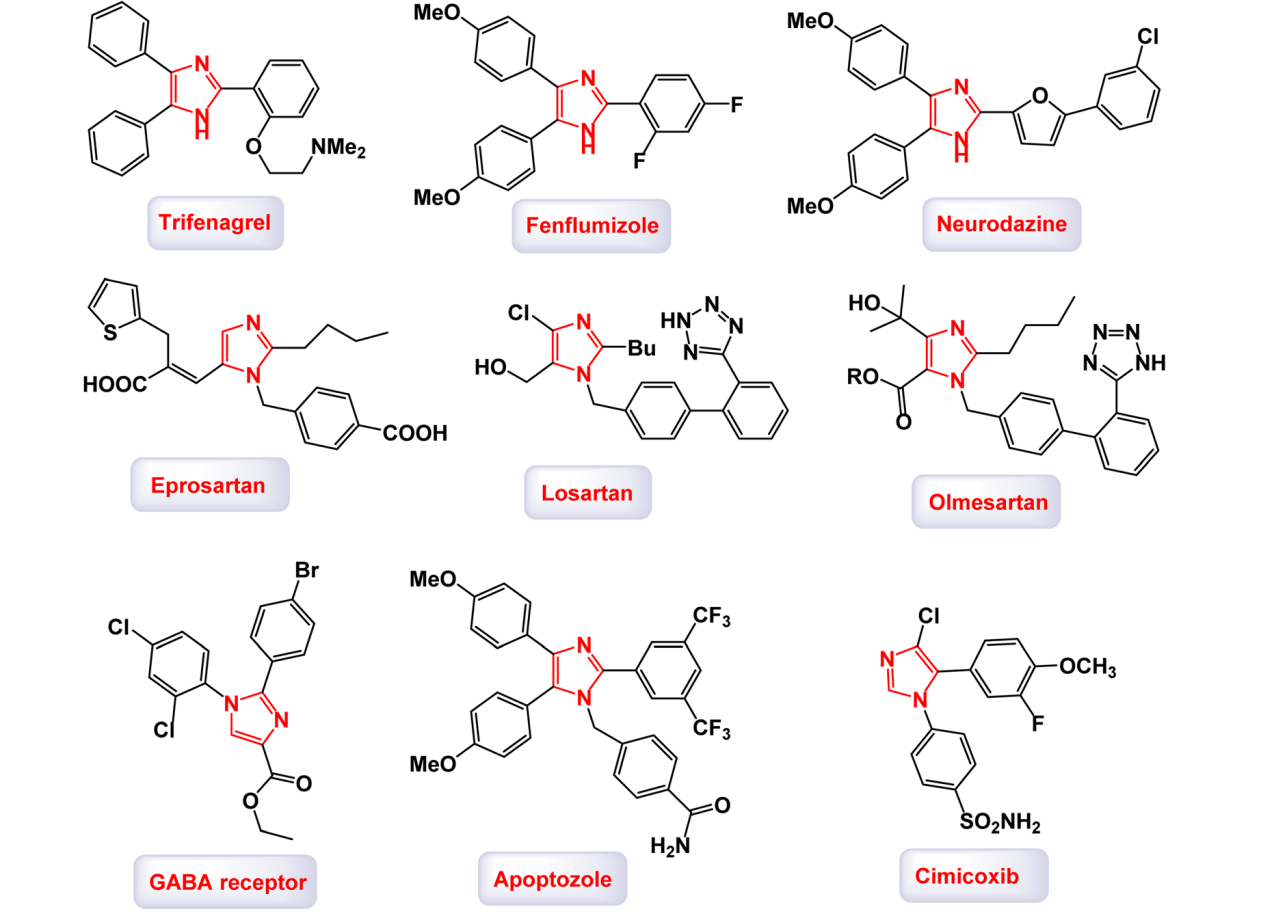
Several drugs and bioactive molecules containing a highly substituted imidazole scaffold.

Highly substituted imidazoles are prevalent in the structure of many drugs and bioactive molecules due to their unique chemical and biological properties.^65^ The illustration in Fig. 6 highlights the structural characteristics of bioactive molecules that incorporate a highly substituted imidazole scaffold.^66^

The imidazole ring, a five-membered heterocyclic structure with two nitrogen atoms, is a versatile pharmacophore capable of interacting with biological targets through hydrogen bonding, π–π stacking, metal coordination, and other molecular interactions.^67^ The introduction of substituents at various positions on the imidazole ring further enhances its biological activity, specificity, and pharmacokinetic properties.^68^ Below is an overview of their presence in drugs and bioactive molecules:

(1) Antifungal drugs

Highly substituted imidazoles are a hallmark of many antifungal drugs that inhibit fungal cytochrome P450 enzymes, disrupting ergosterol synthesis in fungal cell membranes.^69^ Examples include:

✓ *Ketoconazole*: contains multiple substituents that enhance lipophilicity and binding to fungal CYP450.^70^

✓ *Clotrimazole*: a substituted imidazole used to treat fungal infections like athlete's foot and candidiasis.^71^

✓ *Miconazole*: known for its broad-spectrum antifungal activity.^72^

(2) Anticancer drugs

Substituted imidazoles are integrated into anticancer agents due to their ability to interfere with DNA, enzymes, or signaling pathways involved in cancer progression.^73,74^

✓ *Bifonazole derivatives*: show anticancer properties through inhibition of specific enzymes.^75^

✓ *Temozolomide*: an imidazole derivative used in the treatment of glioblastoma and astrocytoma, which works by alkylating DNA.^76^

(3) Antiviral drugs

Imidazole derivatives have been developed to target viral enzymes or proteins, making them effective against viral infections.^77^

✓ *Enfuvirtide analogues*: incorporate imidazole for enhanced interaction with viral proteins. Experimental imidazole derivatives have shown potential against HIV, hepatitis B, and herpes simplex viruses.^78,79^

(4) Anti-inflammatory and analgesic agents

Highly substituted imidazoles exhibit anti-inflammatory and analgesic effects by targeting cyclooxygenase enzymes or other inflammatory mediators.^80^

✓ *Sulconazole*: known for its anti-inflammatory and antifungal dual activity.^81^

✓ *Lornoxicam derivatives*: feature imidazole moieties for enhanced anti-inflammatory effects.^82^

(5) Antihypertensive and cardiovascular drugs

Substituted imidazoles are present in several drugs for cardiovascular diseases, often as angiotensin-converting enzyme (ACE) inhibitors or calcium channel blockers.^83,84^

✓ *Losartan*: contains an imidazole group, used as an angiotensin II receptor blocker (ARB) for treating hypertension.^85^

✓ *Eprosartan*: another ARB with an imidazole moiety that enhances its binding to the AT1 receptor.^86^

(6) Neurological and CNS-active drugs

Substituted imidazoles have applications in treating neurological and psychiatric conditions due to their ability to modulate receptors or enzymes in the central nervous system.^87^

✓ *Triazolam*: a benzodiazepine with an imidazole group, used as a sedative-hypnotic. Experimental imidazole derivatives are being explored for neuroprotective effects in diseases like Alzheimer's.^88^

(7) Enzyme inhibitors

Many enzyme inhibitors rely on substituted imidazoles for their interaction with enzyme active sites.

✓ *Carbonic anhydrase inhibitors*: imidazole derivatives selectively inhibit carbonic anhydrase, useful for glaucoma treatment.^89^

✓ *Nitazoxanide*: a substituted imidazole that inhibits pyruvate : ferredoxin oxidoreductase in protozoa and anaerobic bacteria.^90^

(8) Antibacterial agents

Imidazole derivatives have shown broad-spectrum antibacterial activity by disrupting bacterial DNA, enzymes, or membrane integrity.^91^

✓ *Metronidazole*: a nitroimidazole derivative used to treat bacterial infections, particularly anaerobic bacteria and protozoa.^92^

✓ *Tinidazole*: similar to metronidazole, with an imidazole core and additional substituents for enhanced stability and activity.^93^

(9) Other bioactive molecules

✓ *Histamine H*_*2*_*receptor antagonists*: substituted imidazoles such as cimetidine inhibit stomach acid secretion by targeting H_2_ receptors.^94^

✓ *Biologically active natural products*: some natural products, like pilocarpine (used in glaucoma treatment), contain imidazole derivatives.^94^

Mechanisms of action

The pharmacological activities of substituted imidazoles are often due to their ability to:

✓ Interact with biological targets *via* hydrogen bonding and π–π interactions.^94^

✓ Modulate receptor activity, including G-protein-coupled receptors (GPCRs).^94^

✓ Act as chelating agents for metal ions in enzymes.^94^

✓ Disrupt membrane integrity in pathogens.^94^

These versatile properties make highly substituted imidazoles a significant focus in drug discovery and development.

Advantages of substituted imidazoles in drug design

✓ *Biological target versatility*: substituted imidazoles interact with a wide range of proteins, receptors, and enzymes.

✓ *Tunable properties*: functional groups at different positions on the imidazole ring can modulate solubility, lipophilicity, and binding affinity.

✓ *Stability*: the aromaticity of the imidazole ring confers thermal and chemical stability, enhancing drug shelf life.

In conclusion, highly substituted imidazoles form a critical component of many drugs and bioactive molecules, providing a scaffold that can be modified to achieve diverse biological activities. Their role in the treatment of diseases like infections, cancer, cardiovascular disorders, and neurological conditions underscores their significance in modern medicine.

#### 1.3.2. Chemical importance

Highly substituted imidazoles are chemically important due to the unique properties of the imidazole ring and its ability to accommodate a wide variety of substituents.^95^ These modifications often enhance the reactivity, binding affinity, and specificity of imidazole derivatives, making them versatile in both organic synthesis and medicinal chemistry.^96^ Below are key aspects of their chemical significance:

✓ *Versatile reactivity*: the imidazole ring contains two nitrogen atoms with distinct electronic properties: one is basic (sp^2^-hybridized with a lone pair) and the other is part of an aromatic system. This duality allows for diverse chemical reactions such as nucleophilic and electrophilic substitutions. Substitution at different positions of the imidazole ring (C-2, C-4, C-5, or on nitrogen) can tailor the electronic and steric properties of the compound, enhancing its reactivity or stability in specific environments.^96^

✓ *Coordination chemistry*: imidazole and its derivatives are excellent ligands for coordinating to metal ions due to the lone pair on the nitrogen atoms. Substituents can be introduced to fine-tune the electronic effects, making these derivatives valuable in organometallic chemistry and catalysis.^97^

✓ *Acid-base behavior*: imidazole has an amphoteric nature, meaning it can act as both an acid and a base. This property is exploited in buffer solutions and enzymatic reactions, and as a proton shuttle in biological systems. Highly substituted imidazoles allow chemists to modulate the p*K*_a_ values, enhancing their performance in specific applications like drug design or industrial catalysis.^98^

✓ *Hydrogen bonding and aromaticity*: imidazole derivatives retain aromaticity, making them thermodynamically stable. Substituted imidazoles also exhibit strong hydrogen-bonding capabilities, which are crucial for molecular recognition in biological and chemical systems. These properties make them indispensable in supramolecular chemistry, such as designing molecular sensors or materials.

✓ *Structural versatility*: the imidazole ring can be functionalized at multiple positions, allowing for structural diversity. Substituents such as alkyl, aryl, halogen, or heteroatoms can significantly alter the physicochemical properties, like solubility, lipophilicity, and electronic distribution. This versatility is crucial for optimizing lead compounds in drug discovery or tailoring materials for specific industrial uses.^99^

✓ *Biological mimicry*: imidazole is a key component of histidine, an essential amino acid found in proteins and enzymes. Substituted imidazoles can mimic biological systems or interfere with them, making them valuable in studying biological pathways and designing drugs.^100^

✓ *Applications in organic synthesis*: highly substituted imidazoles are intermediates in the synthesis of complex molecules, including pharmaceuticals, agrochemicals, and dyes. They are used as catalysts, for example, in *N*-heterocyclic carbene (NHC)-mediated reactions, enhancing the efficiency of transformations such as cross-coupling or cycloadditions.^101^

✓ *Photophysical properties*: certain imidazole derivatives exhibit interesting photophysical properties, such as fluorescence or photostability, making them useful in materials science for light-emitting devices, sensors, and imaging applications.^102^

In summary, the chemical importance of highly substituted imidazoles lies in their stability, reactivity, and versatility, which make them valuable in fields ranging from organic synthesis to biological research and materials science. Their ability to combine structural complexity with functional diversity underpins their significance in advancing both fundamental and applied chemistry.

#### 1.3.3. Agricultural importance

Highly substituted imidazoles are of significant importance in agriculture due to their ability to act as bioactive agents, offering solutions for pest control, crop protection, and growth enhancement.^103^ Here's a brief overview:

(1) Fungicides

Substituted imidazoles are widely used as antifungal agents in agriculture. They inhibit fungal cytochrome P450 enzymes, disrupting ergosterol synthesis in fungal cell membranes.^104^ Examples include:

✓ *Prochloraz*: a systemic fungicide used against fungal diseases in crops like wheat and barley.^105^

✓ *Imazalil*: protects fruits, vegetables, and ornamental plants from fungal infections.^106^

(2) Herbicides

Some highly substituted imidazoles function as herbicides, targeting enzymes critical for plant growth.^107^ For instance:

✓ *Imazapyr*: a broad-spectrum herbicide used to control weeds and invasive species in crops and forests.^108^

✓ *Imazethapyr*: selectively used for controlling weeds in legumes like soybeans.^109^

(3) Insecticides

Imidazole derivatives are employed in insect control due to their neurotoxic effects on pests.^110^ Examples include:

✓ *Chlorfenapyr*: used to manage pests in agriculture by disrupting mitochondrial function.^111^

(4) Plant growth regulators

Certain imidazole derivatives are used to regulate plant growth by influencing hormonal pathways, improving yield, and enhancing stress resistance.^112^

(5) Antibacterial agents for plant diseases

Substituted imidazoles are effective against bacterial infections in plants, protecting crops from diseases that can reduce yields.^91^

Advantages in agriculture

✓ *Targeted activity*: high specificity reduces damage to non-target organisms.^113^

✓ *Eco-friendliness*: many imidazole-based compounds are biodegradable and safer for the environment compared to older pesticides.^113^

✓ *Versatility*: they are effective against a wide range of fungi, bacteria, and pests, making them valuable for integrated pest management.^113^

In summary, highly substituted imidazoles are crucial in modern agriculture for ensuring crop health and productivity, contributing to sustainable farming practices.

#### 1.3.4. Green synthesis of highly substituted imidazoles

The green synthesis of highly substituted imidazoles focuses on developing environmentally friendly methods that minimize the use of hazardous substances and reduce waste. Here are some key aspects:

*Green catalysts*: researchers use recyclable and non-toxic catalysts, such as samarium triflate, which can be reused multiple times without significant loss of activity.^114^ These catalysts often operate under mild conditions, reducing energy consumption.^115^

*Eco-friendly solvents*: the use of water or other green solvents instead of harmful organic solvents is a common approach. Some methods even employ solvent-free conditions, further reducing the environmental impact.^116^

*Energy efficiency*: techniques like microwave irradiation and sonication (using ultrasonic waves) are employed to accelerate reactions, making them more energy-efficient.^54^ These methods often lead to higher yields and shorter reaction times.

*Minimized byproducts*: green synthesis aims to produce fewer byproducts, which simplifies purification and reduces waste.^117^ For example, some reactions only produce water as a byproduct.

*Nanocatalysts*: such as iron oxide nanoparticles, improve the efficiency and selectivity of chemical reactions. These catalysts can be easily separated and reused, which aligns with green chemistry principles.

In summary, the eco-friendly synthesis of highly substituted imidazoles represents a significant step toward more sustainable and environmentally conscious chemical processes. These techniques not only lessen the ecological impact but also enhance the efficiency and cost-effectiveness of the synthesis.

## 2. Magnetically recoverable catalysts (MRCs) in synthesis of highly substituted imidazoles

Recent advancements in the synthesis of highly substituted imidazoles have showcased the remarkable potential of magnetic catalysts to enhance reaction efficiency, sustainability, and selectivity. At the forefront of this research, scientists have engineered innovative magnetic nanocatalysts, particularly iron oxide nanoparticles that are functionalized with either acidic or basic ligands. These sophisticated catalysts facilitate crucial transformations, such as multicomponent reactions, under remarkably mild conditions. One of the most impressive features of these magnetic catalysts is their exceptional reusability, which allows them to be employed in multiple reaction cycles without a significant decline in catalytic activity. This quality aligns seamlessly with the principles of green chemistry, promoting environmentally friendly practices in chemical synthesis. Moreover, cutting-edge designs, including bifunctional magnetic catalysts and hybrid systems that integrate magnetic properties with photocatalysis or electrocatalysis, have led to even greater enhancements in catalytic performance. These advanced systems not only maximize efficiency but also expand the versatility of the catalytic processes involved. Furthermore, the development of solvent-free and aqueous reaction environments facilitated by magnetic catalysts significantly mitigates environmental impact while still delivering high yields and outstanding product purity. Nevertheless, several challenges remain, including issues related to catalyst design, such as poor magnetic core stability, loss of catalytic activity upon recycling due to nanoparticle aggregation or leaching, and the use of unstable or weakly coordinating ligands. This review aims to fill this gap by providing a comprehensive overview of recent advances in one-pot syntheses of triaryl and tetrasubstituted aryl imidazole derivatives *via* three- and four-component reactions catalyzed by magnetically recoverable catalysts (MRCs), highlighting current limitations and offering perspectives for catalyst optimization and the development of greener, more sustainable synthetic protocols.

### 2.1. Catalysis by magnetic nanoparticles

Magnetic nanoparticles (MNPs) have emerged as an exciting and innovative class of materials, showcasing remarkable potential in the realm of catalysis.^118^ These minuscule particles, with sizes typically ranging from 1 to 100 nanometers, exhibit distinctive magnetic properties that facilitate their manipulation and separation through the application of external magnetic fields.^119^ This unique feature renders MNPs exceptionally well-suited for various catalytic applications, providing numerous advantages over conventional catalysts.^120^ By leveraging the extraordinary characteristics of magnetic nanoparticles, researchers and industries are empowered to develop more efficient, sustainable, and selective catalytic processes.^119^ This advancement holds promise for addressing critical global challenges in diverse areas, including energy production, materials science, and environmental conservation. The ability to effectively control and utilize MNPs opens up new avenues for innovation and progress, making them a focal point in the ongoing quest for improved catalytic solutions.

**Scheme 1:**
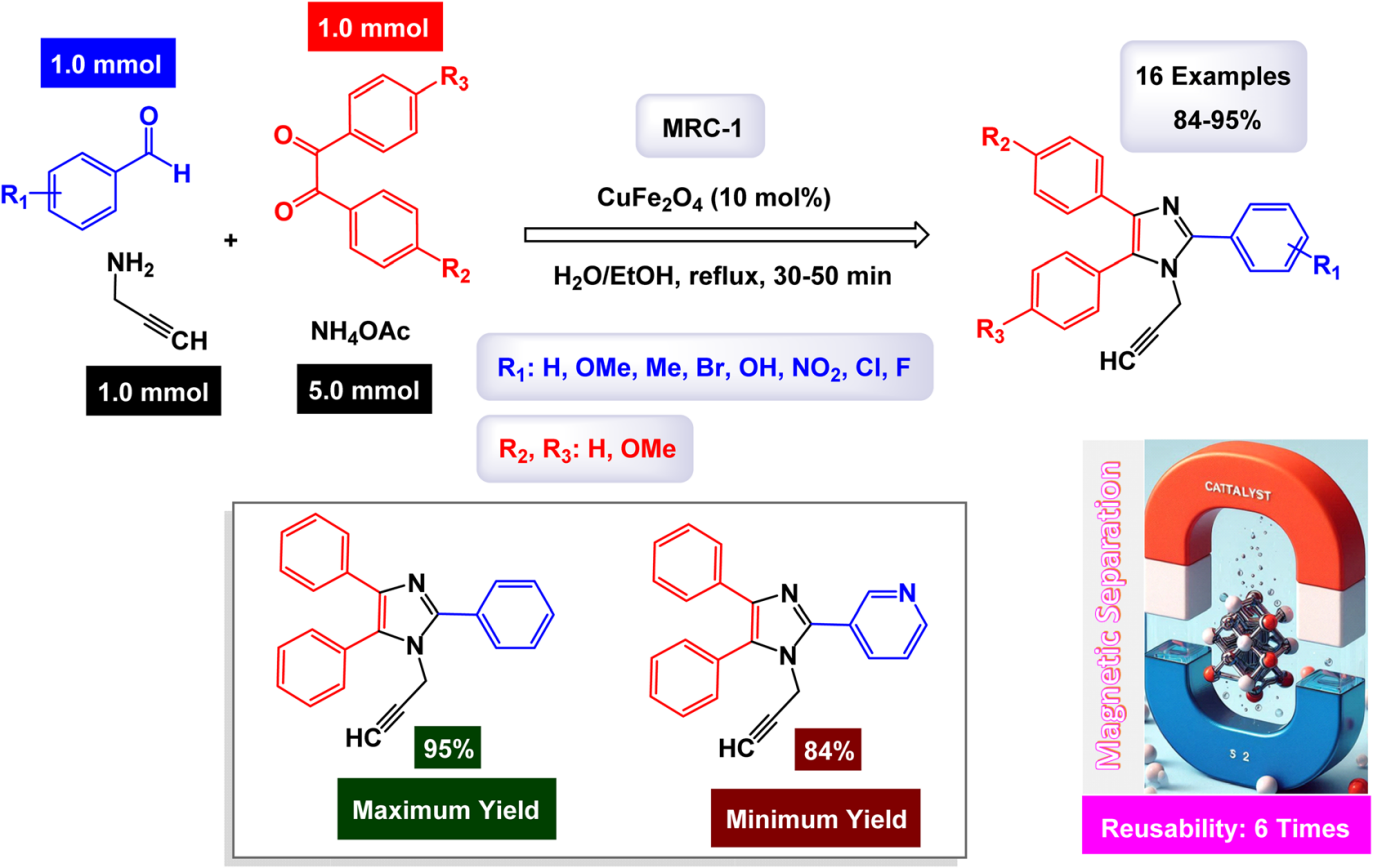
Synthesis of 1,2,4,5-tetrasubstitutedimidazoles [catalysis by CuFe_2_O_4_ NPs].

**Scheme 2:**
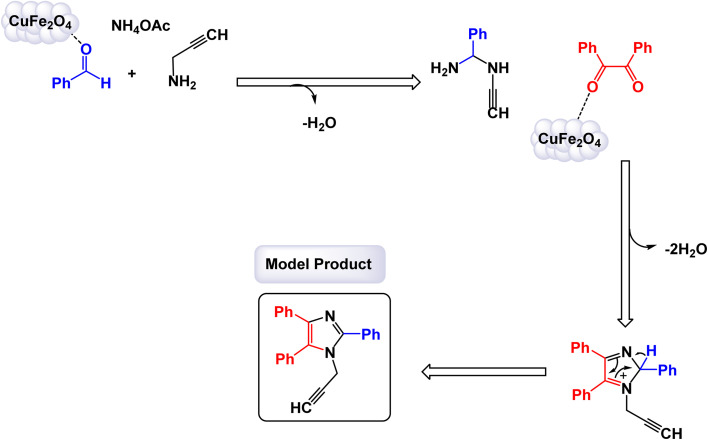
Plausible mechanism for synthesis of 1,2,4,5-tetrasubstitutedimidazoles [catalysis by CuFe_2_O_4_ NPs].

In the quest for a sustainable and eco-friendly approach, non-toxic magnetic CuFe_2_O_4_ nanoparticles have been successfully created and analyzed. These nanoparticles act as effective catalysts in producing new derivatives of 1,2,4,5-tetrasubstituted imidazoles, resulting in remarkable outcomes (as shown in Scheme 1). The dimensions of the magnetic CuFe_2_O_4_ nanoparticles [MRC-1] were meticulously determined using TEM images. Additional confirmation was obtained from XRD patterns, employing Scherrer's equation, which indicated that the average size of these nanoparticles is approximately 23.5 nm.^121^ This finding aligns closely with the data derived from the corresponding histogram analysis. The synthetic pathway proposed (depicted in Scheme 2) suggests that the reaction unfolds through the formation of a diamine intermediate. This intermediate arises from the activation of the carbonyl group present in aldehydes through the interaction with the CuFe_2_O_4_ magnetic nanoparticles. Following this initial step, the diamine undergoes condensation with a 1,2-diketone. This reaction is followed by a dehydration process and subsequent rearrangement *via* an imino intermediate, ultimately yielding the desired imidazole product. This efficient catalytic process showcases the significant potential of these magnetic nanoparticles in organic synthesis. The tests conducted on the CuFe_2_O_4_ nanocatalyst [MRC-1] for recycling demonstrated its remarkable stability and that the catalytic efficiency was retained with only minimal decline after being utilized six times.

**Scheme 3:**
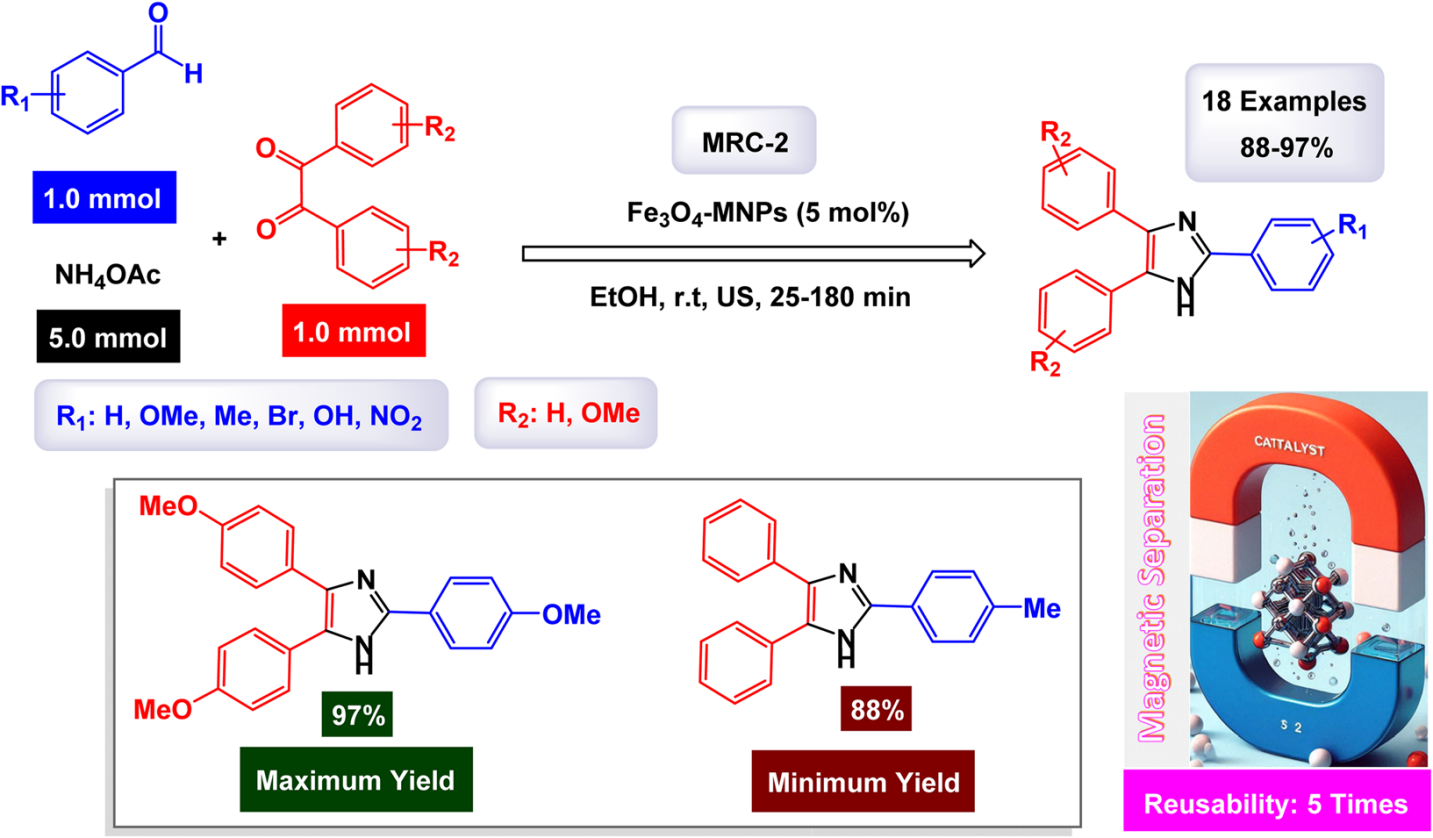
Synthesis of triaryl imidazoles [catalysis by Fe_3_O_4_ NPs].

Safari and Zarnegar extensively explored the catalytic potential of Fe_3_O_4_ nanoparticles (NPs) in the efficient synthesis of 2,4,5-trisubstituted imidazoles.^122^ Their approach involves a one-pot, three-component reaction that combines various benzil derivatives, benzaldehydes, and ammonium acetate, all conducted in an ethanol medium under the influence of ultrasonic irradiation, as illustrated in Scheme 3. The SEM image of the magnetite nanoparticles reveals a mean diameter of approximately 18 nm and a predominantly spherical morphology, findings that align closely with the XRD analysis results. This study highlights the innovative application of these nanoparticles in organic synthesis, showcasing their effective role in facilitating complex chemical reactions. The tests conducted on the Fe_3_O_4_ nanocatalyst [MRC-2] for recycling demonstrated its remarkable stability, and that the catalytic efficiency was retained with only minimal decline after being utilized five times.

**Scheme 4:**
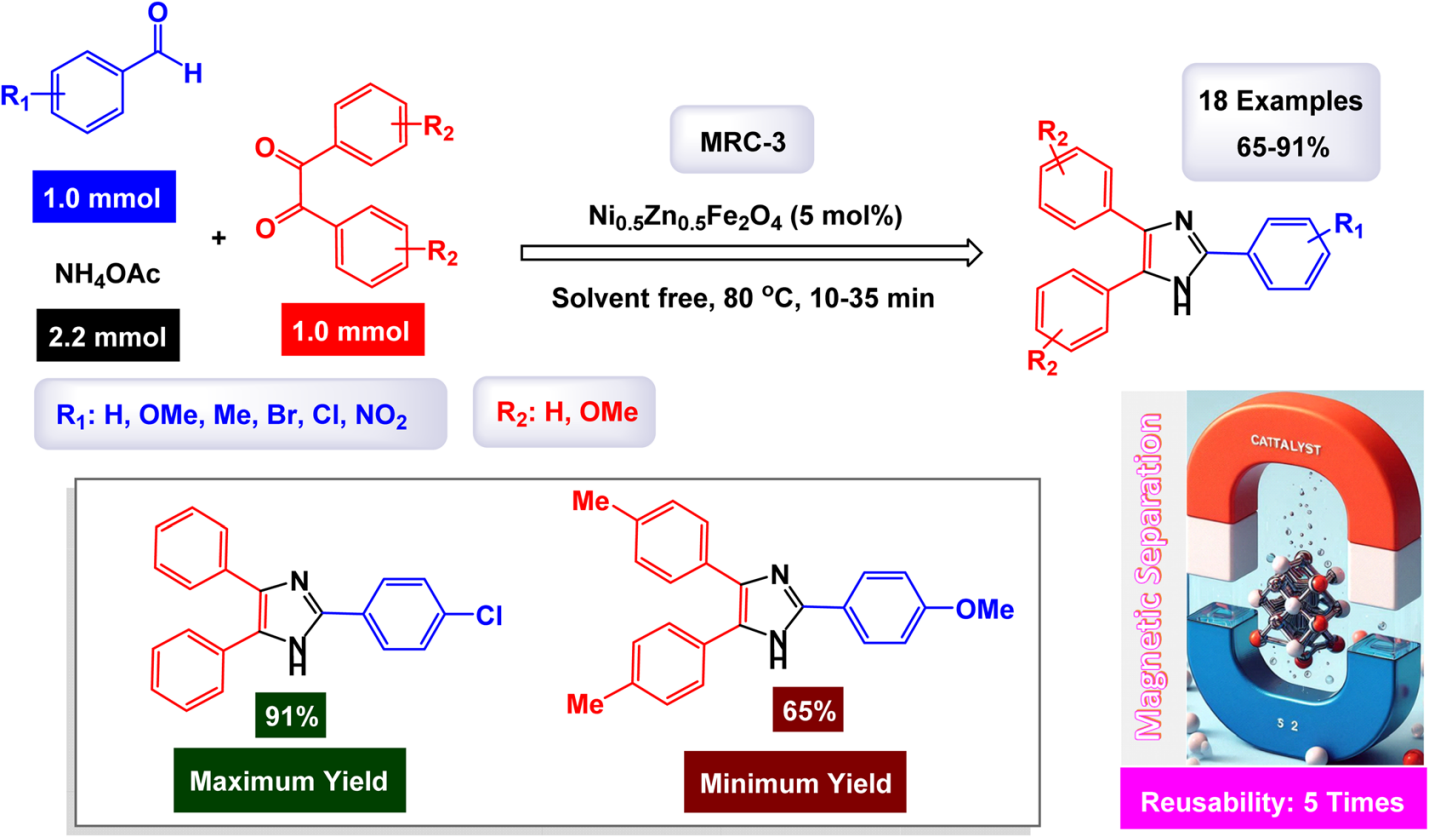
Synthesis of triaryl imidazoles [catalysis by Ni_0.5_Zn_0.5_Fe_2_O_4_ NPs].

**Scheme 5:**
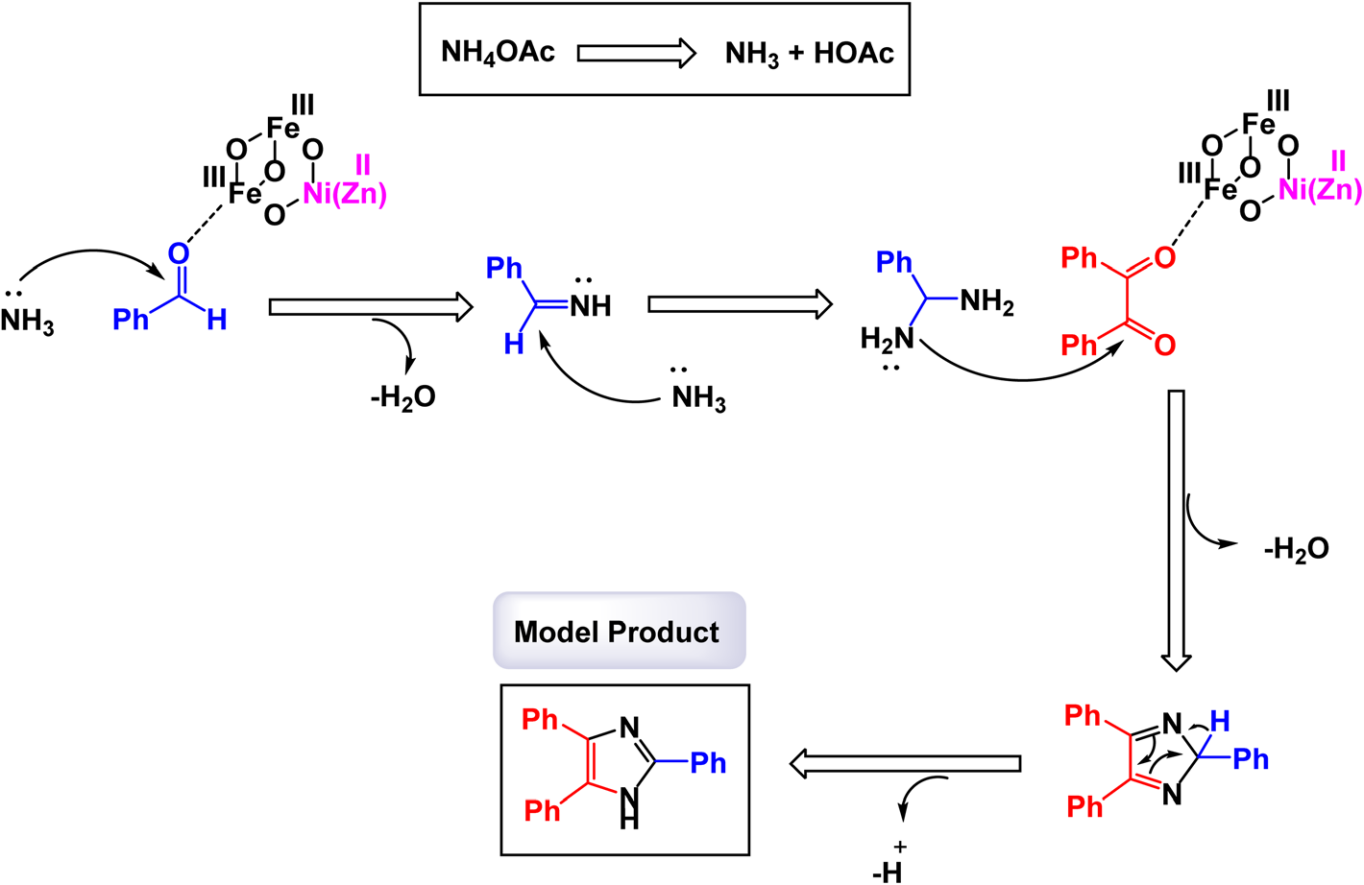
Plausible mechanism for synthesis of triaryl imidazoles [catalysis by Ni_0.5_Zn_0.5_Fe_2_O_4_ NPs].

The research group led by Khazaei has made significant advancements in the development of a novel nanomaterial, specifically Ni_0.5_Zn_0.5_Fe_2_O_4_, which demonstrated remarkable efficiency as a heterogeneous catalyst.^123^ This nanocatalyst was thoroughly characterized using various analytical techniques including IR, EDX, XRD, and SEM. These analyses confirmed its structural integrity and composition. The Ni_0.5_Zn_0.5_Fe_2_O_4_ nanocatalyst is employed in a one-pot, three-component reaction, enabling the synthesis of 2,4,5-triaryl substituted imidazoles under solvent-free conditions, as outlined in Scheme 4. The presence of different elements in the catalyst's structure was verified through EDX and elemental mapping techniques, emphasizing its effective performance in catalyzing the desired reactions. According to the proposed synthetic mechanism depicted in Scheme 5, the process begins with ammonia, which is treated with ammonium acetate (NH_4_OAc). This ammonia interacts with the carbonyl group of an activated aldehyde, resulting in the formation of 1,2-diamine. In the following step, this 1,2-diamine reacts with an activated benzyl group, leading to the elimination of two water molecules and yielding an intermediate compound. Subsequently, a hydrogen shift from this intermediate produces the final product, 2,4,5-triaryl imidazoles. Although kinetic data and theoretical studies such as DFT calculations are not available, the catalytic activity of Ni_0_._5_Zn_0_._5_Fe_2_O_4_ can likely be attributed to the presence of surface Lewis acidic sites, particularly Fe^3+^ ions, which play a crucial role in activating the carbonyl group of the aldehyde and stabilizing key reaction intermediates. The spinel structure further facilitates electron transfer and provides a high surface area with accessible active sites, which may account for the high catalytic efficiency observed under solvent-free conditions. This concise yet impactful methodology showcases the versatility and effectiveness of the Ni_0.5_Zn_0.5_Fe_2_O_4_ nanocatalyst in organic synthesis. The tests conducted on the Ni_0.5_Zn_0.5_Fe_2_O_4_ nanocatalyst [MRC-3] for recycling demonstrated its remarkable stability, and that the catalytic efficiency was retained with only minimal decline after being utilized five times.

**Scheme 6:**
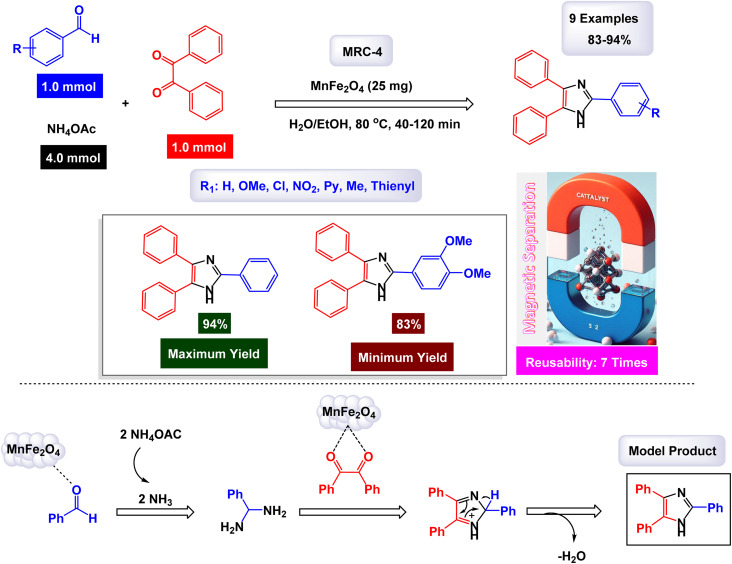
Synthesis of triaryl imidazoles [catalysis by MnFe_2_O_3_ nanoparticles].

In a groundbreaking study focused on the application of magnetic nanoparticles as catalysts, the research team led by Mahmudzadeh investigated the use of MnFe_2_O_4_ nanoparticles, denoted as MRC-4, for the synthesis of triaryl imidazoles.^124^ To fabricate these cubic manganese ferrite (MnFe_2_O_4_) nanoparticles, the researchers employed a chemical co-precipitation method, which facilitated the production of high-quality nanostructures. The catalytic performance of these manganese ferrite nanoparticles was meticulously assessed in the context of synthesizing triaryl imidazole compounds. Analysis using SEM and TEM revealed that the nanoparticles exhibited an average size ranging between 20 and 30 nanometers, a finding that was further corroborated by XRD results. Under the carefully optimized experimental conditions outlined in Scheme 6, the research demonstrated that aldehydes featuring different substituents significantly influenced the reaction outcomes. Notably, aldehydes possessing electron-withdrawing groups, such as nitro, reacted more swiftly compared to those with electron-donating substituents. This observation indicates that the chemical structure of the reactants plays a crucial role in determining both the rate and the mechanism of the reaction.

The process begins with the interaction of two equivalents of ammonia (NH_3_) and a quaternary ammonium salt (QAC), which serves as a nucleophilic precursor. The QAC, due to its unique structure, acts as a source of nucleophilic species, which are crucial for the subsequent reaction steps. The catalyst plays a crucial role by providing an active surface that enhances the reactivity of these substrates.

Introduction of the reactants to the MnFe_2_O_4_ catalyst promotes the formation of an intermediate complex. This complex stabilizes the transition state, thereby lowering the activation energy required for subsequent reactions. As the reaction progresses, the catalyst aids in aligning and orienting the reactants favorably for bond formation. Specifically, it facilitates the nucleophilic attack on the electrophilic carbon centers present in the aromatic rings. The catalytic action continues as additional reagents are introduced into the system. The MnFe_2_O_4_ nanoparticles not only enhance the rate of the reaction but also ensure selectivity towards the desired triaryl imidazole product. This is achieved through their unique electronic properties, such as their ability to influence electron density, and surface characteristics, which can facilitate charge transfer during critical steps of the reaction. For instance, the surface of MnFe_2_O_4_ nanoparticles can act as a site for electron transfer, thereby influencing the reactivity of the substrates.

As depicted in Scheme 6, after several transformation steps involving condensation and cyclization processes, water is eliminated, leading to the formation of triaryl imidazole. Throughout this mechanism, MnFe_2_O_4_ acts as a reusable catalyst, a feature that reaffirms its sustainability and economic viability. Its structural integrity allows it to maintain activity over multiple cycles without significant loss in performance. This feature is particularly advantageous in synthetic chemistry, where catalyst recovery and reusability are essential for economic viability and sustainability. MnFe_2_O_4_ nanoparticles serve as an effective catalyst by providing an active site that accelerates reaction kinetics while ensuring selectivity towards triaryl imidazoles. Their ability to stabilize intermediates and lower activation barriers underscores their significance in this synthetic pathway, highlighting their potential application in broader catalytic processes within organic chemistry.

The catalytic behavior of MnFe_2_O_4_ is likely governed by the presence of surface Lewis acidic sites and a synergistic interaction between Fe^3+^ and Mn^2+^ ions, which together contribute to the activation of carbonyl functionalities and stabilization of reactive intermediates during imidazole formation. Moreover, the stability and reusability of the MnFe_2_O_4_ nanocatalyst [MRC-4] were evaluated through a series of recycling tests. Remarkably, the catalyst retained its catalytic efficiency with only a minimal decrease in performance even after being employed seven times, underscoring its practical applicability and robustness in synthetic processes.

### 2.2. Catalysis by magnetic nanoparticles modified with organocatalysts

**Scheme 7:**
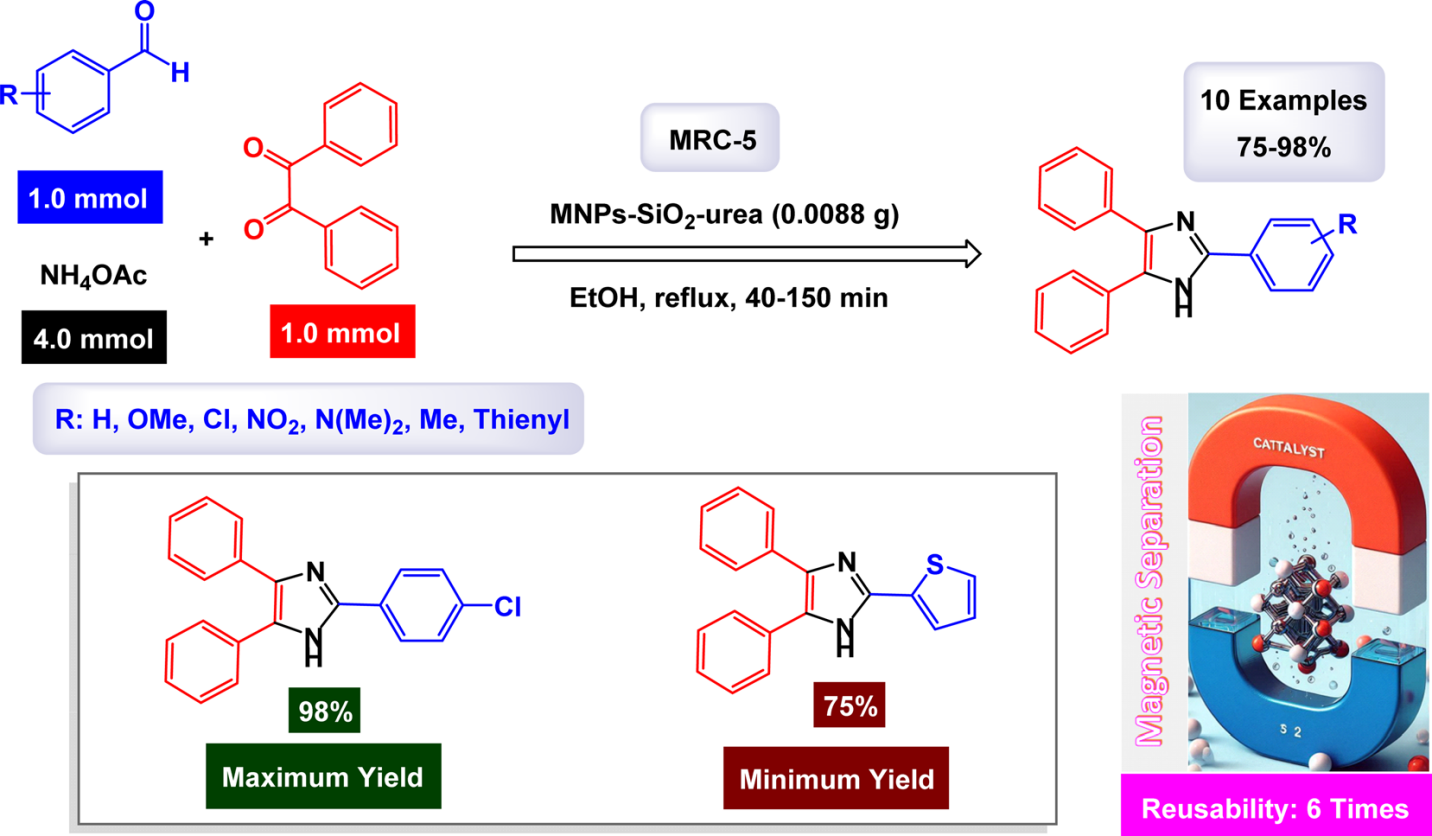
Synthesis of triaryl imidazoles [catalysis by the MNPs-SiO_2_-urea nanocomposite].

In a recent publication from Maleki's research group, they introduced an innovative nanoparticle designed with a core–shell structure, utilizing a urea-functionalized silica base. This advanced hybrid nanoparticle demonstrated remarkable efficiency as a heterogeneous nanocatalyst. The study highlighted its ability to facilitate a one-pot three-component condensation reaction, involving benzil or benzoin, along with a range of substituted aldehydes and ammonium acetate (Scheme 7). The reaction was performed under mild conditions, resulting in the successful synthesis of various imidazoles, showcasing the nanoparticle's recoverability and effectiveness in catalysis.^125^ The experiments carried out on the MNPs-SiO_2_-urea nanocatalyst, referred to as [MRC-5], showcased impressive stability during the recycling process. Remarkably, this nanocatalyst retained its catalytic efficiency with only a slight decrease in performance, even after being employed in six consecutive cycles. This durability highlights the potential of [MRC-5] for sustainable applications in catalysis.

**Scheme 8:**
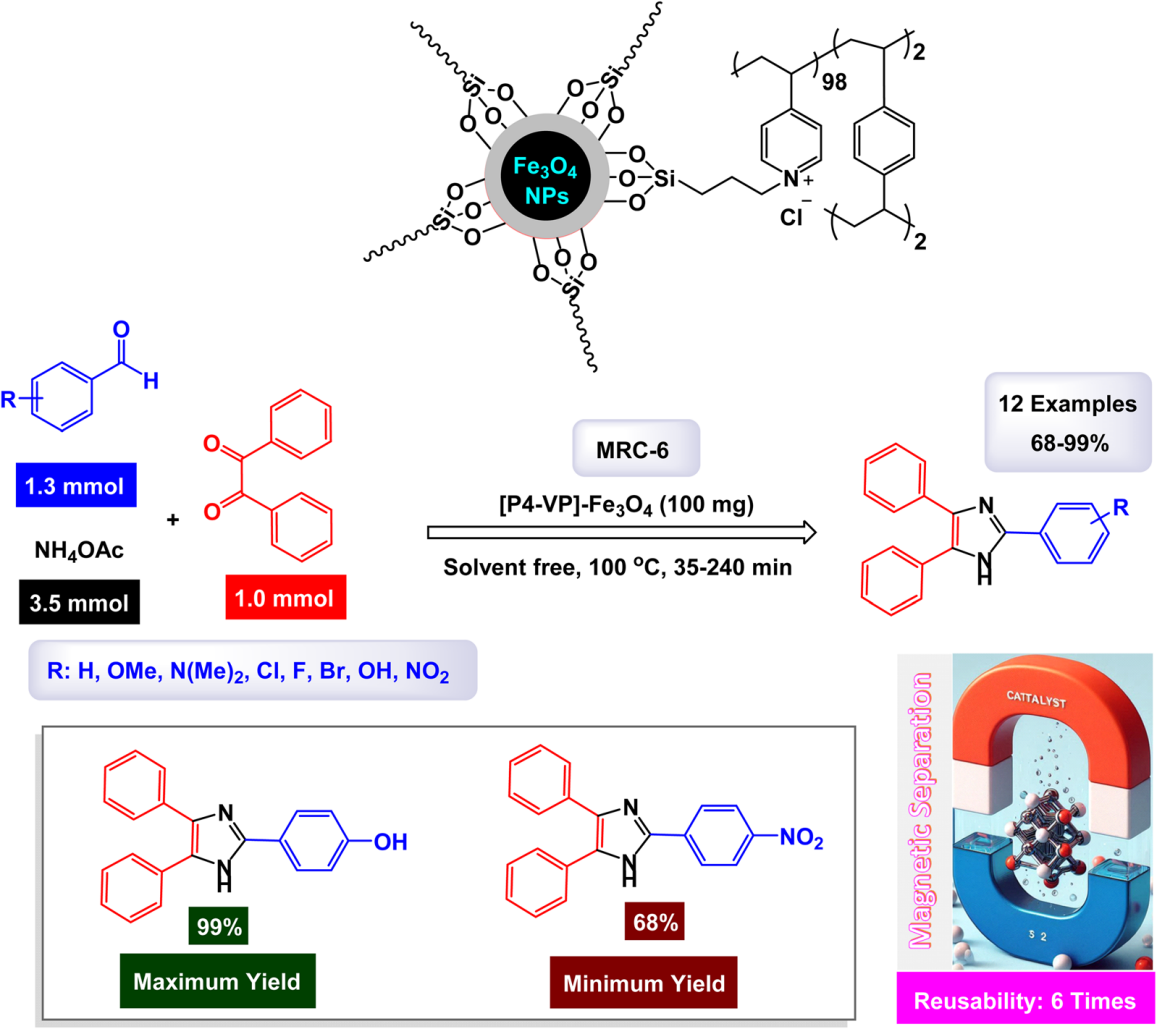
Synthesis of triaryl imidazoles [catalysis by the [P_4_-VP]-Fe_3_O_4_ nanocomposite].

**Scheme 9:**
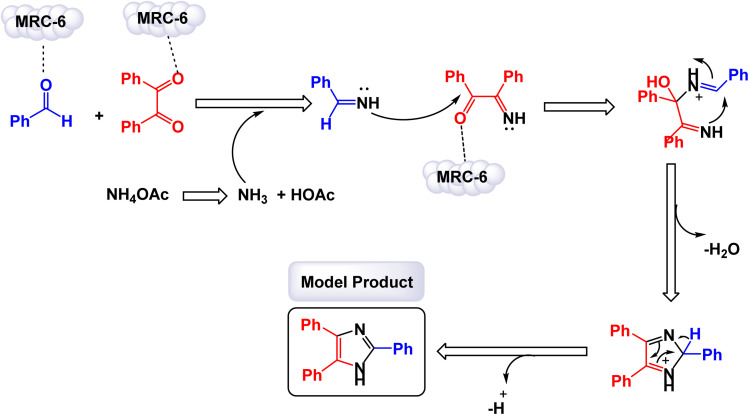
Plausible mechanism for synthesis of triaryl imidazoles [catalysis by the [P_4_-VP]-Fe_3_O_4_ nanocomposite].

Hamidi and Ali Karimi Zarchi reported an innovative approach to catalyst development with the construction of cross-linked poly(4-vinylpyridine) supported Fe_3_O_4_ nanoparticles, which are referred to as [P_4_-VP]-Fe_3_O_4_ NPs.^126^ This novel magnetic polymeric catalyst has shown significant promise in efficiently facilitating the synthesis of imidazole derivatives. The catalytic activity of these nanoparticles was impressive, yielding a range of imidazole products with high efficiency, achieving yields between 68% and 99%. Notably, these reactions occurred in a remarkably short time frame, ensuring that the resulting products maintained high purity, as illustrated in Scheme 8. The mechanism proposed for this catalytic process, detailed in Scheme 9, begins with the formation of imine intermediates. This occurs through the nucleophilic addition of ammonia to an activated aldehyde, as well as to an activated 1,2-diketone. Following this initial step, the imine intermediate undergoes a condensation reaction with the carbonyl carbon of the 1,2-diketone imine, resulting in the creation of a carbocation. This carbocation is then attacked by the nitrogen atom of the imine, leading to the formation of an iminium ion. The subsequent cyclization, accompanied by dehydration, yields iso-imidazole. The polymeric ligand likely facilitates substrate activation, in addition to the acidic synergistic effect provided by the iron ions. Finally, this iso-imidazole rearranges through a^1,5^ sigmatropic hydrogen shift to form the desired imidazoles, efficiently completing the synthesis. The tests conducted on the [P_4_-VP]-Fe_3_O_4_ NPs nanocatalyst [MRC-6] for recycling demonstrated its remarkable stability, and that the catalytic efficiency was retained with only minimal decline after being utilized six times.

**Scheme 10:**
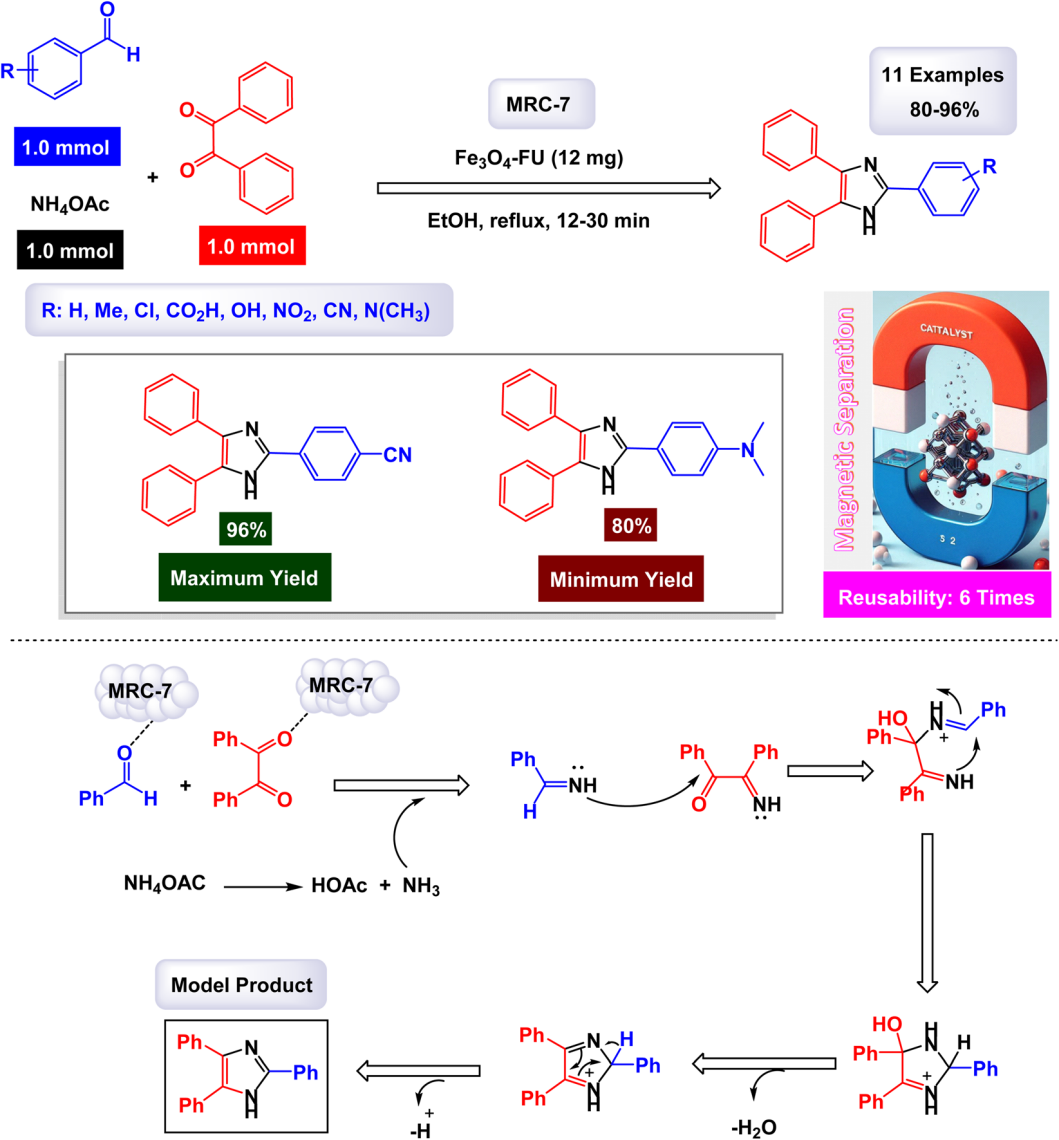
Synthesis of triaryl imidazoles [catalysis by Fe_3_O_4_-FU NPs].

In the synthesis of triaryl imidazoles depicted in Scheme 10, the catalyst, a [P_4_-VP]-Fe_3_O_4_ nanocomposite, is a key player in the reaction mechanism. The process is initiated with the interaction of the starting materials, an aromatic aldehyde and an amine, in the presence of the catalyst. The [P_4_-VP]-Fe_3_O_4_ nanocomposite, with its unique properties, significantly boosts the reactivity of these substrates by providing an active surface that fosters effective collisions between reactants. As the reaction commences, the catalyst stabilizes the formation of a key intermediate through its ability to coordinate with the electrophilic carbon atom of the aromatic aldehyde. This interaction lowers the activation energy required for nucleophilic attack by the amine, allowing for a more favorable transition state. The catalyst not only facilitates this initial step but also ensures that the orientation of reactants is optimal for subsequent reactions. After the initial nucleophilic attack, a series of transformations, including condensation and cyclization steps, lead to the formation of triaryl imidazole. Throughout these stages, the catalyst, [P_4_-VP]-Fe_3_O_4_, remains actively involved, maintaining an environment that promotes reactivity. Its surface characteristics play a crucial role in stabilizing intermediates and promoting electron transfer processes, which are essential for bond formation.

Another significant advantage of using [P_4_-VP]-Fe_3_O_4_ as a catalyst is its recyclability. After completing a reaction cycle, it can be easily separated from the product mixture and reused in subsequent reactions without substantial loss in catalytic activity. This feature not only enhances economic efficiency but also aligns with sustainable practices in synthetic chemistry, making it a practical choice for organic synthesis. The [P_4_-VP]-Fe_3_O_4_ nanocomposite catalyst is vital in orchestrating the synthesis of triaryl imidazoles by lowering activation barriers and stabilizing reactive intermediates throughout the mechanism. Its ability to facilitate critical steps while remaining recoverable underscores its importance in advancing efficient and sustainable organic synthesis methodologies.

**Scheme 11:**
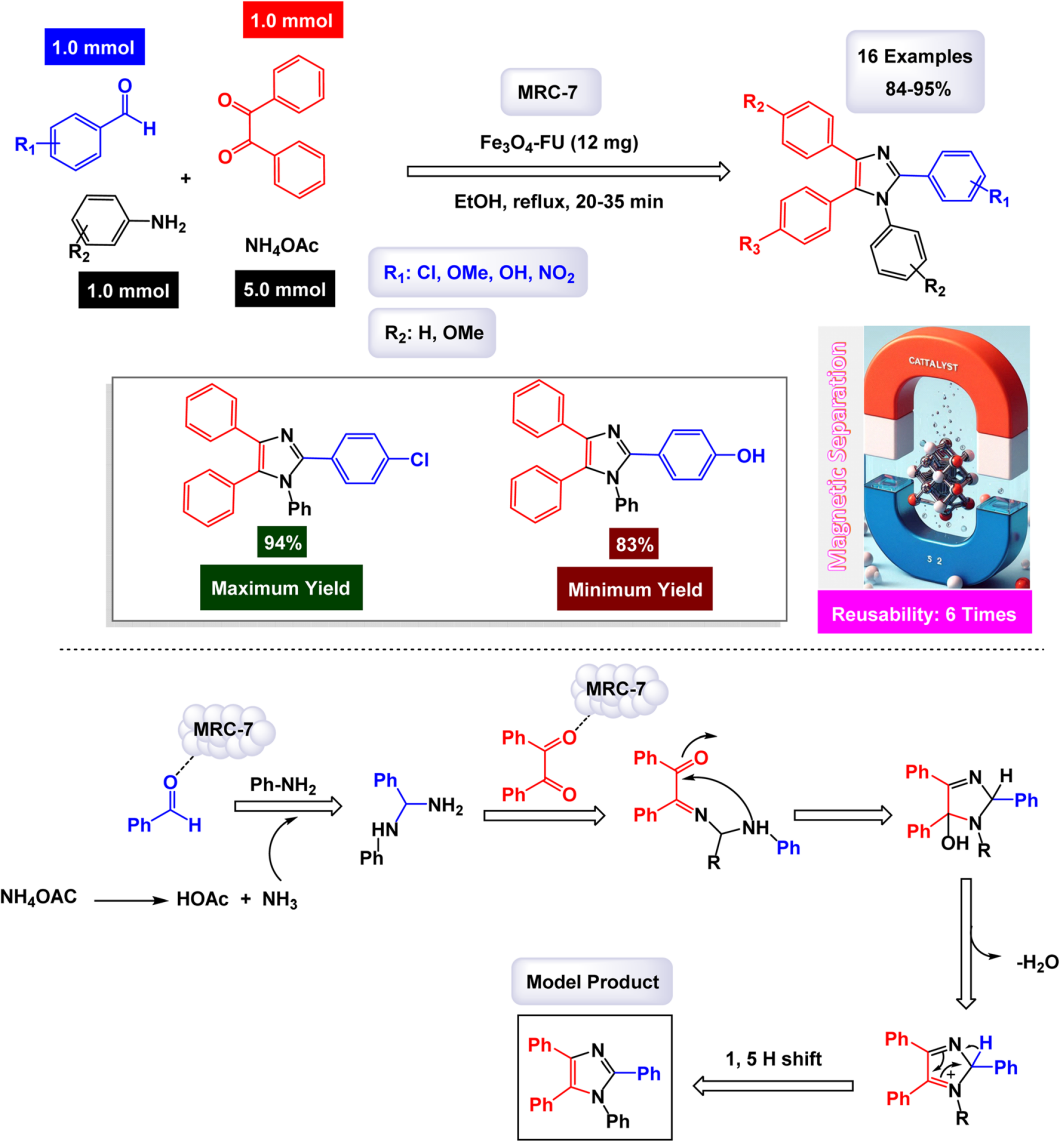
Synthesis of 1,2,4,5-tetrasubstituted imidazoles [catalysis by Fe_3_O_4_-FU NPs].

In a captivating research report, Javanshir and his team introduced a novel and highly effective methodology for the one-pot multicomponent synthesis of tri- and tetra-substituted imidazoles.^127^ This innovative approach hinges on the use of the Fe_3_O_4_@FU nanomaterial, which is derived from fucoidan (FU), a fucose-rich sulfated polysaccharide obtained from the brown algae *Fucus vesiculosus*. Fe_3_O_4_@FU serves as a magnetically reusable catalyst (denoted as MRC-7), making the process both efficient and environmentally friendly. The successful modification of the iron oxide nanoparticles (Fe_3_O_4_ NPs) with fucoidan was confirmed through advanced spectroscopic techniques, with FT-IR providing clear evidence of this transformation. Additionally, the catalyst's impressive magnetic properties were validated by VSM analysis. As detailed in Schemes 10 and 11, the methodology involves three- and four-component reactions that utilize benzyl groups, aldehydes, and ammonium acetate (NH_4_OAc), with the latter enhanced by the introduction of amines in the tetra-substituted imidazole formation. Under reflux conditions in ethanol, the Fe_3_O_4_@FU nanomaterial effectively catalyzes these reactions, leading to the generation of tri- and tetra-substituted imidazole products in exceptionally high yields, where the functional groups present in fucoidan, such as SO_3_H, may participate in hydrogen bonding or coordination interactions with the substrates, thereby facilitating substrate activation and stabilizing key intermediates during the catalytic cycle.

In the synthesis of triaryl imidazoles illustrated in Scheme 10, the catalyst, Fe_3_O_4_@FU nanoparticles, plays a pivotal role in facilitating the reaction mechanism. The process begins with the activation of the starting materials, which include an aromatic aldehyde and an amine. The presence of the Fe_3_O_4_@FU catalyst enhances the reactivity of these substrates by providing an active surface that promotes effective interactions. The unique properties of the nanoparticles, such as their ability to interact favorably with the aromatic aldehyde and lower the activation energy for nucleophilic attack, make them effective catalysts. This interaction leads to the formation of a key intermediate, which the catalyst stabilizes, making it easier for the reaction to proceed. These unique properties not only facilitate the initial step but also ensure that subsequent transformations occur efficiently. As the reaction progresses, condensation and cyclization steps occur, culminating in the formation of triaryl imidazole. Throughout these stages, the catalyst continues to play a pivotal role by maintaining an environment conducive to reactivity. It aids in stabilizing intermediates and, importantly, promotes electron transfer processes necessary for forming new bonds, a unique property that piques scientific curiosity.

Another significant advantage of using Fe_3_O_4_@FU nanoparticles as a catalyst is their recyclability. After completing a reaction cycle, they can be easily separated from the product mixture and reused in subsequent reactions without significant loss in catalytic activity. This feature not only enhances economic efficiency but also aligns with sustainable practices in synthetic chemistry, making them a valuable catalyst in the field. Fe_3_O_4_@FU nanoparticles are crucial in orchestrating the synthesis of triaryl imidazoles by lowering activation barriers and stabilizing reactive intermediates throughout the mechanism. Their ability to facilitate critical steps while remaining recoverable underscores their importance in advancing efficient and sustainable organic synthesis methodologies.

The overall process highlights not only the versatility of the catalyst but also the promising potential for broader applications in the synthesis of complex organic compounds. The tests conducted on the Fe_3_O_4_@FU nanocatalyst [MRC-7] for recycling demonstrated its remarkable stability, and that the catalytic efficiency was retained with only minimal decline after being utilized six times.

**Scheme 12:**
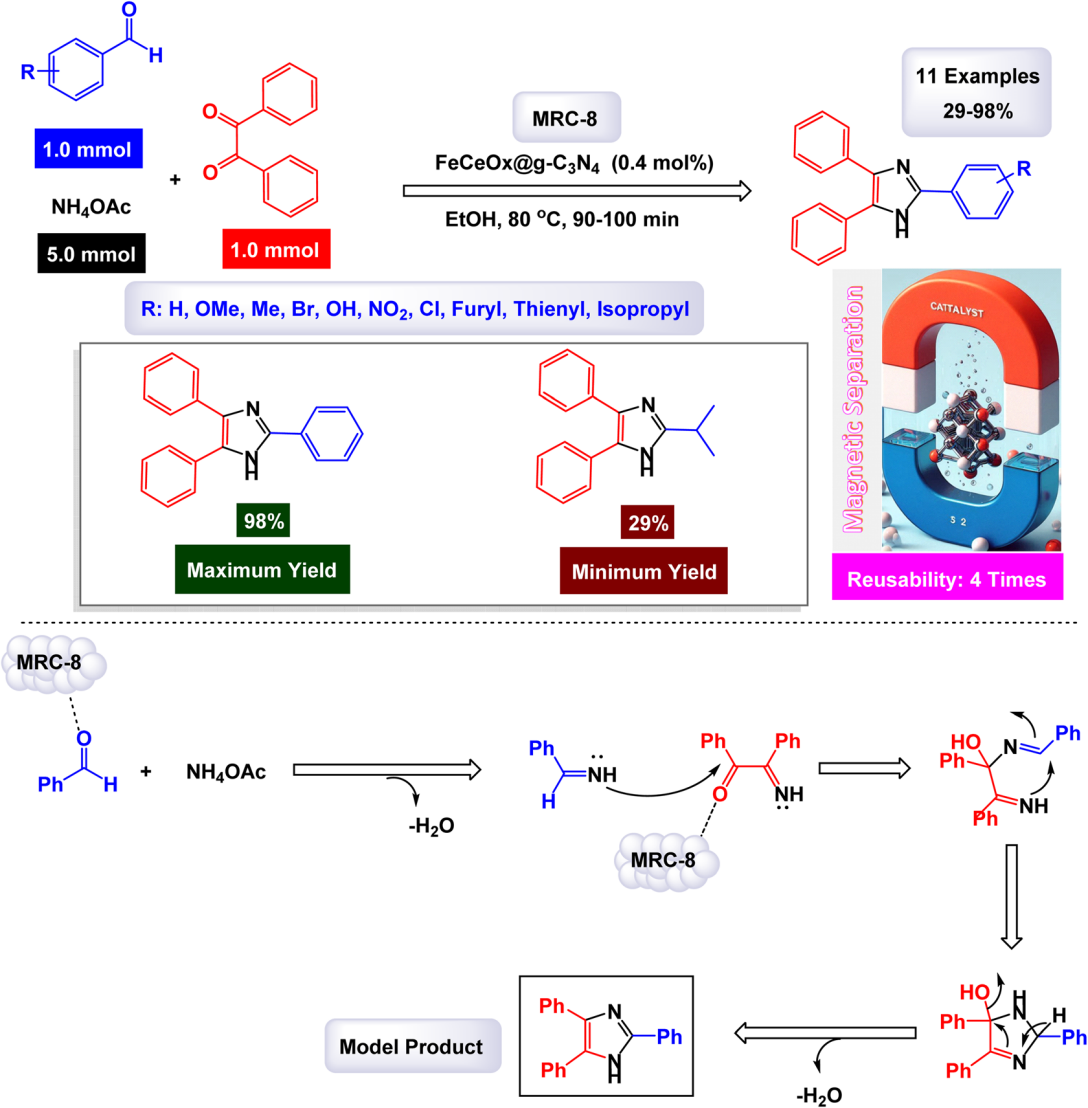
Synthesis of triaryl imidazoles [catalysis by the FeCeO_*x*_@g-C_3_N_4_ nanomaterial].

**Scheme 13:**
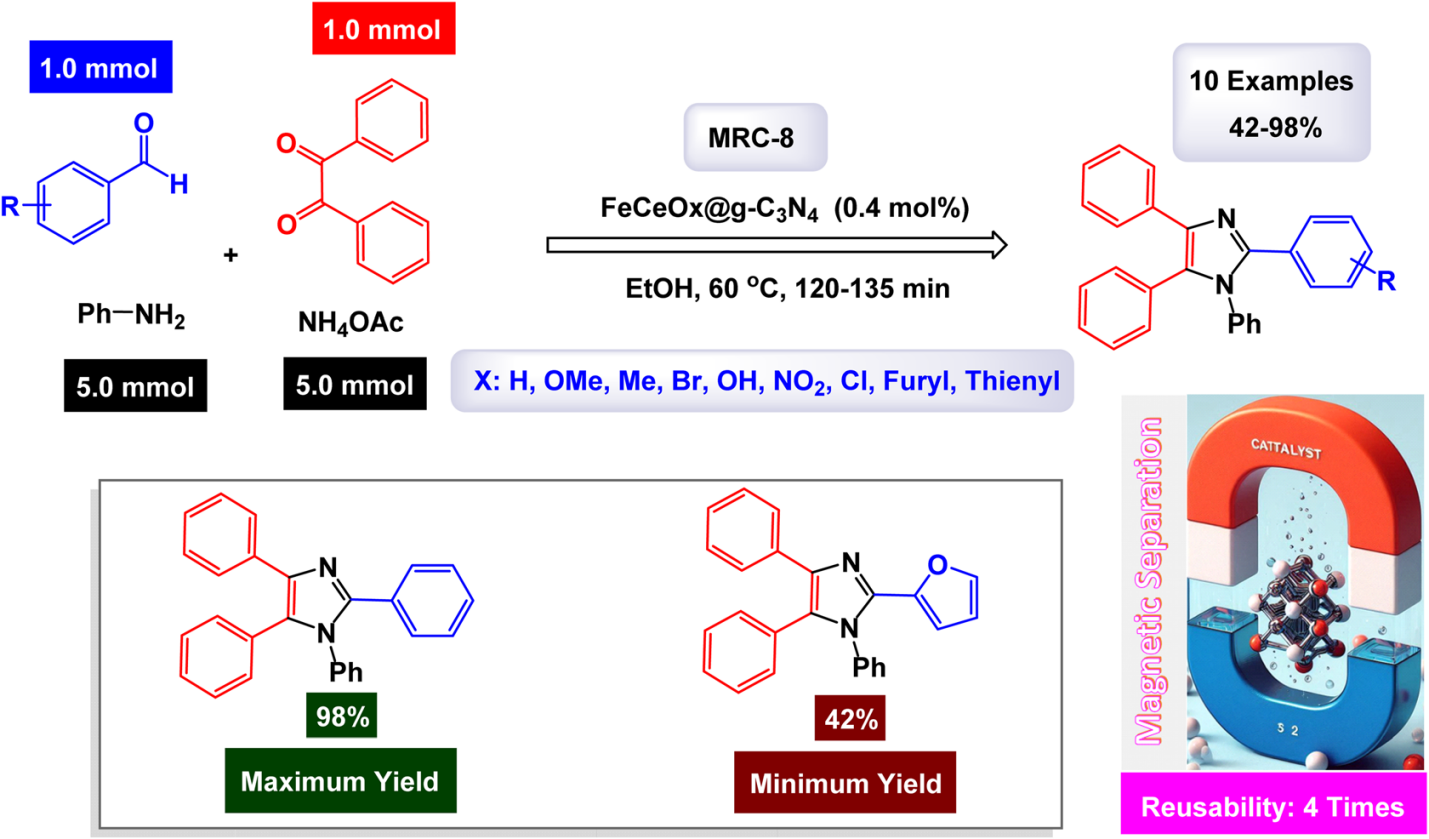
Synthesis of 1,2,4,5-tetrasubstituted imidazoles [catalysis by the FeCeO_*x*_@g-C_3_N_4_ nanomaterial].

Saadat and his team of researchers engineered a groundbreaking composite material known as FeCeO_*x*_@g-C_3_N_4_. This innovative catalyst was synthesized through a series of advanced techniques, including sonication, sintering, and hydrothermal processing. Their studies focused on the catalyst's exceptional activity in the one-pot synthesis of complex imidazole derivatives, specifically 2,4,5-trisubstituted and 1,2,4,5-tetrasubstituted imidazoles, as illustrated in Schemes 12 and 13. The structural characteristics of the FeCeO_*x*_@g-C_3_N_4_ nanocatalyst, denoted as [MRC-8], were meticulously analyzed using various analytical methods.^128^ FT-IR was employed to explore the presence and nature of chemical bonds within the material. SEM and TEM provided detailed images that revealed both the surface morphology and internal architecture of the catalyst. Furthermore, TGA was conducted to evaluate the thermal stability of the compound, while EDX offered insights into its elemental composition. Additionally, XRD analysis was performed to determine the crystalline structure of the composite. In the reaction pathway depicted in Scheme 13, the FeCeO_*x*_@g-C_3_N_4_ catalyst is essential for forming an imine intermediate. This happens when the ammonium acetate's amine group adds nucleophilically to an aldehyde's carbonyl group. Afterward, the imine intermediate interacts with a benzyl compound, with the nitrogen atom of the imine functioning as a nucleophile. This leads to a nucleophilic attack on the electrophilic carbon of the benzyl group. Following this attack, a significant rearrangement transpires within the molecular structure, ultimately yielding a trisubstituted imidazole product. This rearrangement involves the migration of substituents within the intermediate, a process that is crucial for attaining the desired imidazole derivatives.

In the synthesis of triaryl imidazoles depicted in Scheme 12, the FeCeO_*x*_@g-C_3_N_4_ nanomaterial catalyst plays a vital role in facilitating the reaction mechanism. The process begins with the interaction of an aromatic aldehyde and an amine, which are crucial starting materials for the formation of imidazole derivatives. The presence of the FeCeO_*x*_@g-C_3_N_4_ catalyst enhances the reactivity of these substrates by providing an active surface that promotes effective molecular interactions. Initially, the aromatic aldehyde is activated upon contact with the catalyst, allowing it to adopt a favorable orientation that lowers the activation energy required for nucleophilic attack by the amine. This interaction leads to the formation of a key intermediate, where the catalyst stabilizes this transition state, thereby facilitating its conversion into further reaction products. The unique properties of the FeCeO_*x*_@g-C_3_N_4_ nanomaterial not only assist in this critical initial step but also ensure that subsequent transformations proceed efficiently.

As the reaction progresses through condensation and cyclization steps, resulting in the formation of triaryl imidazoles, the catalyst continues to play an essential role by maintaining an environment conducive to reactivity. It stabilizes various intermediates formed during these stages and promotes necessary electron transfer processes that are vital for forming new bonds. Additionally, one significant advantage of using FeCeO_*x*_@g-C_3_N_4_ as a catalyst is its recyclability. After completing a reaction cycle, this nanomaterial can be easily separated from the product mixture and reused in subsequent reactions without significant loss in catalytic activity. This feature enhances economic efficiency and aligns with sustainable practices in synthetic chemistry. The FeCeO_*x*_@g-C_3_N_4_ nanomaterial is instrumental in orchestrating the synthesis of triaryl imidazoles by lowering activation barriers and stabilizing reactive intermediates throughout the mechanism. Its ability to facilitate critical steps while remaining recoverable underscores its importance in advancing efficient and sustainable organic synthesis methodologies.

The precision and effectiveness of this catalytic process underscore the potential applications of FeCeO_*x*_@g-C_3_N_4_ in synthetic chemistry. Moreover, the presence of cerium oxide (CeO_2_) in the FeCeO_*x*_@g-C_3_N_4_ nanocomposite likely enhances both catalytic activity and stability, exerting a synergistic effect that boosts the yield of trisubstituted imidazole. The tests conducted on the FeCeO_*x*_@g-C_3_N_4_ nanocatalyst [MRC-8] for recycling demonstrated its remarkable stability, and that the catalytic efficiency was retained with only minimal decline after being utilized four times.

**Scheme 14:**
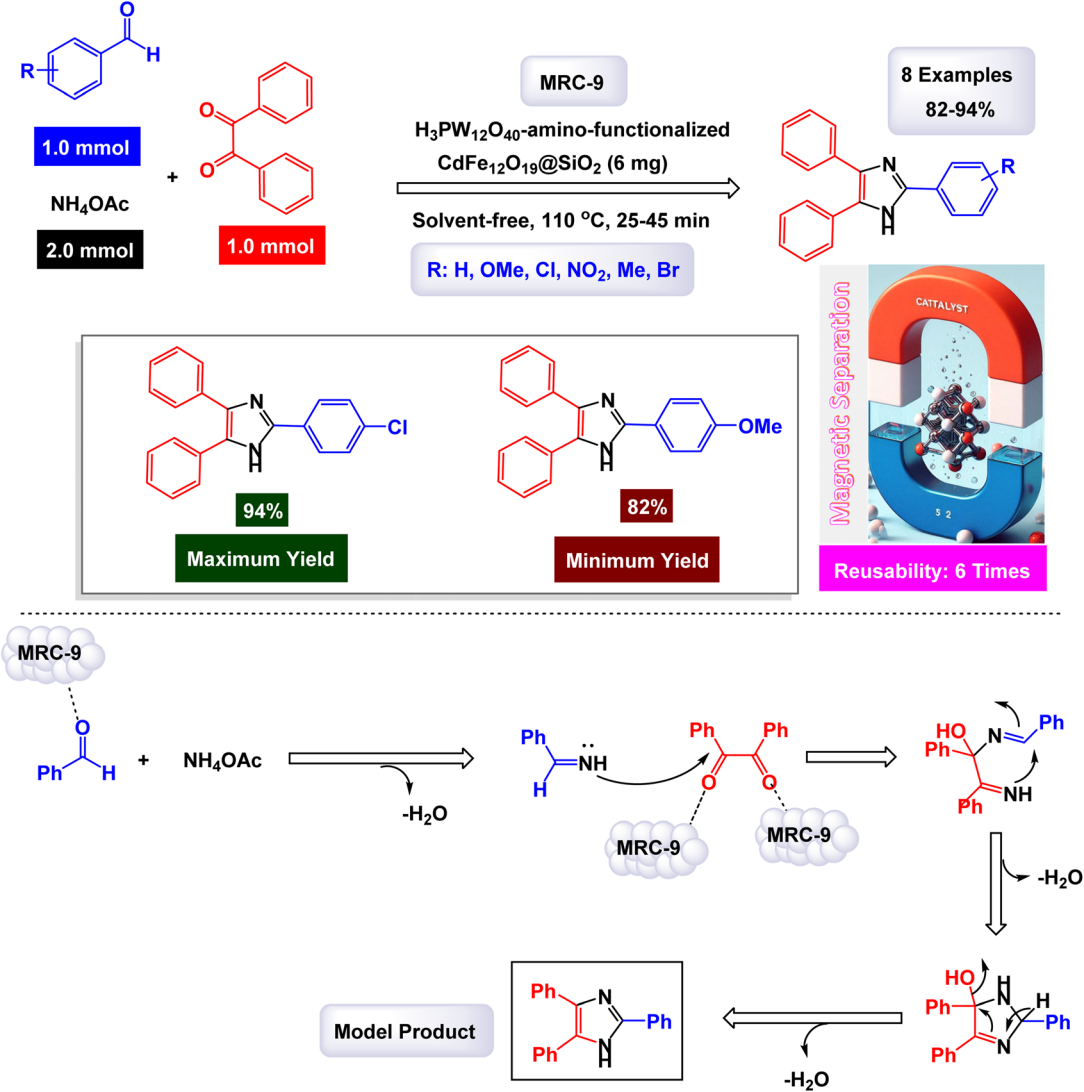
Synthesis of triaryl imidazoles [catalysis by the H_3_PW_12_O_40_-amino-functionalized CdFe_12_O_19_@SiO_2_ nanomaterial].

The Safaie-Ghomi research group pioneered a comprehensive and effective methodology for synthesizing triaryl imidazoles through a one-pot, three-component reaction involving benzil, ammonium acetate, and various benzaldehydes.^129^ This innovative process leverages a specially designed nanomaterial, H_3_PW_12_O_40_-amino-functionalized CdFe_12_O_19_@SiO_2_, enabling the reaction to take place under solvent-free conditions. As illustrated in Scheme 14, the reaction is believed to initiate with the formation of a diamine intermediate. This occurs when the nanocatalyst activates the carbonyl group of the benzaldehyde, facilitating its condensation with two molecules of ammonia. Following this initial reaction, the resultant intermediate undergoes further condensation with the carbonyl carbons of benzil. A subsequent dehydration step leads to the formation of an imino intermediate, which ultimately rearranges to yield the desired triaryl imidazole product. The catalytically active sites are most likely the H_3_PW_12_O_40_ (HPA) units anchored onto the support, which serve as strong Brønsted acid sites responsible for activating the carbonyl group of the aldehyde. This sophisticated mechanism not only showcases the efficiency of the nanocatalyst but also highlights the potential for environmentally friendly chemical processes.

In the synthesis of triaryl imidazoles illustrated in Scheme 14, the catalyst H_3_PW_12_O_40_-amino-functionalized CdFe_2_O_4_@SiO_2_ nanomaterial plays a crucial role in facilitating the reaction mechanism. The process begins with the interaction of an aromatic aldehyde and an amine, which are essential starting materials for generating imidazole derivatives. The presence of this catalyst significantly enhances the reactivity of these substrates by providing a conducive surface that promotes effective molecular interactions. Initially, the aromatic aldehyde is activated upon contact with the H_3_PW_12_O_40_-amino-functionalized CdFe_2_O_4_@SiO_2_ catalyst, allowing it to adopt a favorable orientation that lowers the activation energy required for nucleophilic attack by the amine. This interaction leads to the formation of a key intermediate, where the catalyst stabilizes this transition state, facilitating its conversion into further reaction products. The unique properties of this nanomaterial not only assist in this critical initial step but also ensure that subsequent transformations proceed efficiently.

As the reaction continues through condensation and cyclization steps, resulting in the formation of triaryl imidazoles, the catalyst maintains an environment conducive to reactivity. It stabilizes various intermediates formed during these stages and promotes necessary electron transfer processes vital for forming new bonds. The amino functionalization on the catalyst surface enhances its ability to interact with reactants, further increasing its effectiveness. Moreover, one significant advantage of using H_3_PW_12_O_40_-amino-functionalized CdFe_2_O_4_@SiO_2_ as a catalyst is its recyclability. After completing a reaction cycle, this nanomaterial can be easily separated from the product mixture and reused in subsequent reactions without significant loss in catalytic activity. This characteristic not only improves economic efficiency but also aligns with sustainable practices in synthetic chemistry. The H_3_PW_12_O_40_-amino-functionalized CdFe_2_O_4_@SiO_2_ nanomaterial is instrumental in orchestrating the synthesis of triaryl imidazoles by lowering activation barriers and stabilizing reactive intermediates throughout the mechanism. Its ability to facilitate critical steps while remaining recoverable underscores its significance in advancing efficient and sustainable organic synthesis methodologies.

The tests conducted on the H_3_PW_12_O_40_-amino-functionalized CdFe_12_O_19_@SiO_2_ nanocatalyst [MRC-9] demonstrated its remarkable stability, and that the catalytic efficiency was retained with only minimal decline after being utilized six times.

**Scheme 15:**
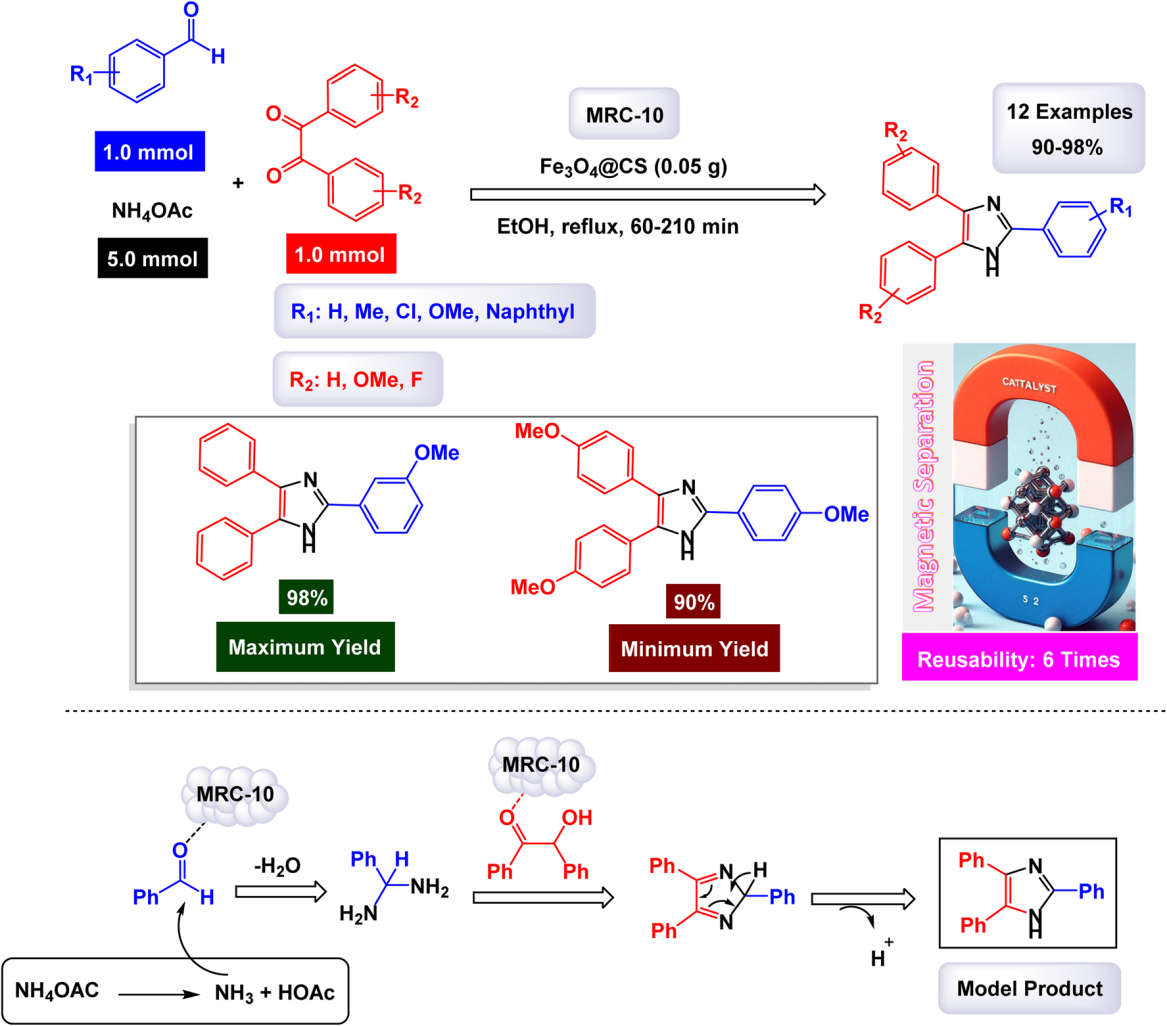
Synthesis of triaryl imidazoles [catalysis by the Fe_3_O_4_@CS nanocomposite].

In a noteworthy study conducted by Safari and Zarnegar, chitosan-coated iron oxide nanoparticles, referred to as Fe_3_O_4_@CS [MRC-10], were developed and utilized as an effective heterogeneous catalyst for the one-pot synthesis of 2,4,5-trisubstituted imidazoles (Scheme 15).^130^ This innovative procedure involved the condensation of benzil derivatives, aryl aldehydes, and ammonium acetate in an ethanol (EtOH) medium. The abundant free OH and NH_2_ groups on the chitosan-supported Fe_3_O_4_ surface actively enhance the activation of the aldehyde carbonyl group. The successful immobilization of chitosan onto the magnetic nanoparticles was confirmed through FT-IR, demonstrating the effective integration of the two components. Additionally, SEM images provided clear visual evidence of the proper support of Fe_3_O_4_ nanoparticles on chitosan, illustrating a well-structured composite material that enhances catalytic activity.

In the synthesis of triaryl imidazoles depicted in Scheme 15, the Fe_3_O_4_@CS nanocomposite catalyst plays a pivotal role in facilitating the reaction mechanism. The process begins with the interaction of an aromatic aldehyde and an amine, which are essential starting materials for generating imidazole derivatives. The presence of the Fe_3_O_4_@CS catalyst enhances the reactivity of these substrates by providing a surface that promotes effective molecular interactions. Initially, when the aromatic aldehyde comes into contact with the catalyst, it undergoes activation. This activation allows the aldehyde to adopt a favorable orientation that lowers the activation energy required for nucleophilic attack by the amine. As a result, a key intermediate is formed, wherein the catalyst stabilizes this transition state and facilitates its conversion into further reaction products. The unique properties of the Fe_3_O_4_@CS nanocomposite not only assist in this critical initial step but also ensure that subsequent transformations proceed efficiently.

As the reaction progresses through condensation and cyclization steps, leading to the formation of triaryl imidazoles, the catalyst continues to maintain an environment conducive to reactivity. It stabilizes various intermediates formed during these stages and promotes necessary electron transfer processes that are vital for forming new bonds. The composition of the nanocomposite enhances its ability to interact with reactants, thereby increasing its overall effectiveness. Moreover, one significant advantage of using Fe_3_O_4_@CS as a catalyst is its recyclability. After completing a reaction cycle, this nanocomposite can be easily separated from the product mixture and reused in subsequent reactions without significant loss in catalytic activity. This feature not only improves economic efficiency but also aligns with sustainable practices in synthetic chemistry. The Fe_3_O_4_@CS nanocomposite is instrumental in orchestrating the synthesis of triaryl imidazoles by lowering activation barriers and stabilizing reactive intermediates throughout the mechanism. Its ability to facilitate critical steps while remaining recoverable underscores its importance in advancing efficient and sustainable organic synthesis methodologies.

The tests conducted on the Fe_3_O_4_@CS nanocatalyst [MRC-10] demonstrated its remarkable stability, and that the catalytic efficiency was retained with only minimal decline after being utilized six times.

### 2.3. Catalysis by magnetic nanoparticle supported ionic liquids

**Scheme 16:**
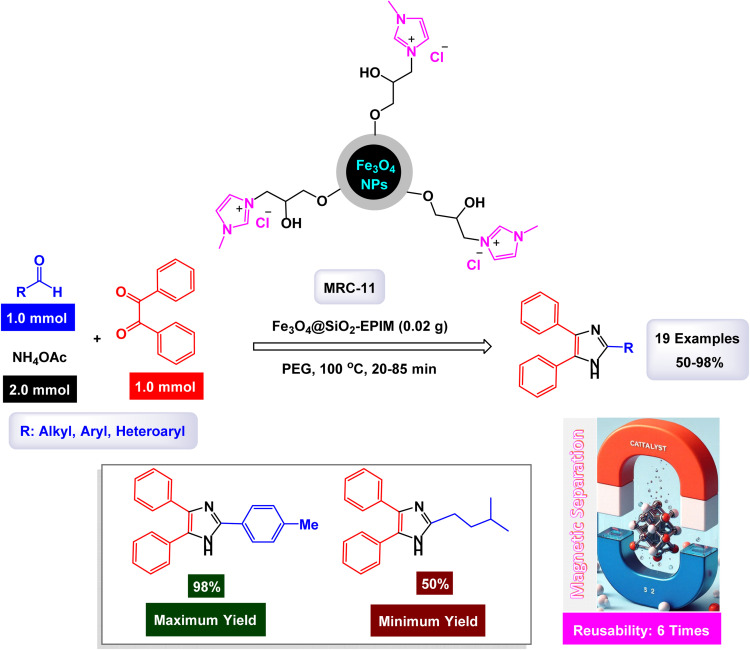
Synthesis of 1,2,4,5-tetrasubstitutedimidazoles [catalysis by the Fe_3_O_4_@SiO_2_-EPIM nanocomposite].

Ionic liquids are a type of compound that remains in a liquid form at room temperature and is made up of ions.^131^ They are recognized for their distinct properties, such as low volatility, high thermal stability, and the ability to dissolve diverse substances.^132^ These features render them outstanding solvents and catalysts in various chemical processes.^133^ When used as catalysts, ionic liquids can improve reactions' speed and selectivity, playing a vital role in green chemistry due to their reusability and limited environmental impact.^134,135^ Their design can be customized for particular reactions, making them adaptable tools in contemporary synthetic chemistry. In a captivating study, the research team led by Khalifeh successfully designed and synthesized a novel green nanocatalyst, identified as Fe_3_O_4_@SiO_2_ modified with epichlorohydrin and 1-methyl-imidazole (Fe_3_O_4_@SiO_2_-EPIM).^95^ This innovative catalyst was evaluated for its effectiveness in facilitating the synthesis of trisubstituted imidazoles through one-pot condensation reactions involving benzil, an aldehyde, and ammonium acetate. The reactions were conducted under optimal conditions, specifically at a temperature of 100 °C with a catalyst dose of 0.02 g, as illustrated with PEG 200 (Scheme 16). The structural characterization of the nanocatalyst revealed that the silicon dioxide (SiO_2_) shell possesses a thickness of approximately 25 nm, while the embedded magnetic nanoparticles exhibit an average diameter of around 10 nm, as observed in the TEM and SEM images. A detailed mechanism for the condensation reactions-engaging benzil, benzaldehyde, and ammonium acetate-catalyzed by the Fe_3_O_4_@SiO_2_-EPIM nanocomposite [MRC-11] is illustrated in Scheme 18. In this process, the desired product emerges through a series of steps that include dehydration, subsequent rearrangement, and the elimination of a proton, leading to the formation of the targeted trisubstituted imidazoles. The quaternary ammonium cation anchored on the surface of Fe_3_O_4_@SiO_2_-EPIM serves as the active site, activating the aldehyde carbonyl group through electrostatic interaction, thereby making it more susceptible to nucleophilic attack due to ammonia generated from ammonium acetate.

**Scheme 17:**
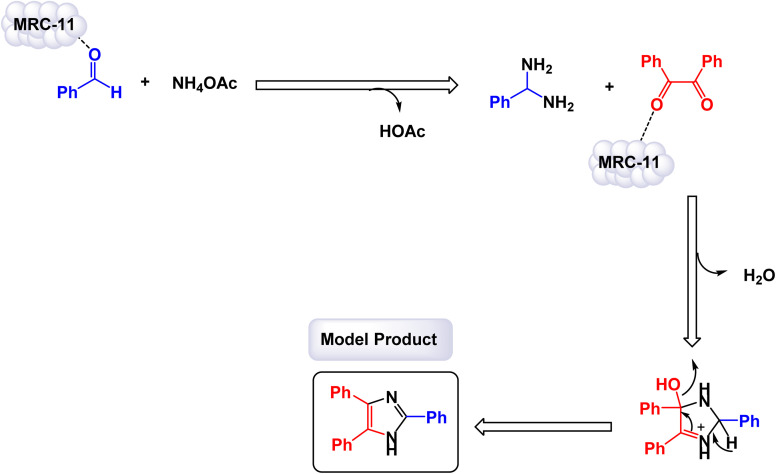
Plausible mechanism for synthesis of 1,2,4,5-tetrasubstitutedimidazoles [catalysis by the Fe_3_O_4_@SiO_2_-EPIM nanocomposite (MRC-11)].

In the synthesis of 1,2,4,5-tetrasubstituted imidazoles as illustrated in Scheme 17, the Fe_3_O_4_@SiO_2_-EPIM nanocomposite catalyst plays an essential role in facilitating the reaction mechanism. The process begins with the interaction between an aromatic aldehyde and an acetamide derivative, which serves as the initial substrates for generating the desired imidazole derivatives. The presence of this catalyst significantly enhances the reactivity of these substrates by providing a surface that promotes effective molecular interactions. Initially, when the aromatic aldehyde interacts with the catalyst, it is activated through coordination to the iron species present in the Fe_3_O_4_@SiO_2_-EPIM nanocomposite. This activation lowers the energy barrier for nucleophilic attack due to the acetamide, allowing for a more favorable transition state to form. As a result, a key intermediate is generated, wherein the catalyst stabilizes this transition state and facilitates its conversion into further reaction products.

As the reaction progresses through subsequent steps involving condensation and cyclization, leading to the formation of tetrasubstituted imidazoles, the catalyst continues to play a critical role. It stabilizes various intermediates formed during these stages and promotes necessary electron transfer processes vital for forming new bonds. The unique properties of the Fe_3_O_4_@SiO_2_-EPIM nanocomposite enhance its ability to interact with reactants effectively, thereby increasing its overall catalytic efficiency. Additionally, one of the significant advantages of using Fe_3_O_4_@SiO_2_-EPIM as a catalyst is its recyclability. After completing a reaction cycle, this nanocomposite can be easily separated from the product mixture and reused in subsequent reactions without a substantial loss in catalytic activity. This characteristic not only improves economic efficiency but also aligns with sustainable practices in synthetic chemistry. The Fe_3_O_4_@SiO_2_-EPIM nanocomposite is instrumental in orchestrating the synthesis of 1,2,4,5-tetrasubstituted imidazoles by lowering activation barriers and stabilizing reactive intermediates throughout the mechanism. Its ability to facilitate critical steps while remaining recoverable underscores its significance in advancing efficient and sustainable methodologies in organic synthesis.

The tests conducted on the Fe_3_O_4_@SiO_2_-EPIM nanocatalyst [MRC-11] demonstrated its remarkable stability, and that the catalytic efficiency was retained with only minimal decline after being utilized six times.

**Scheme 18:**
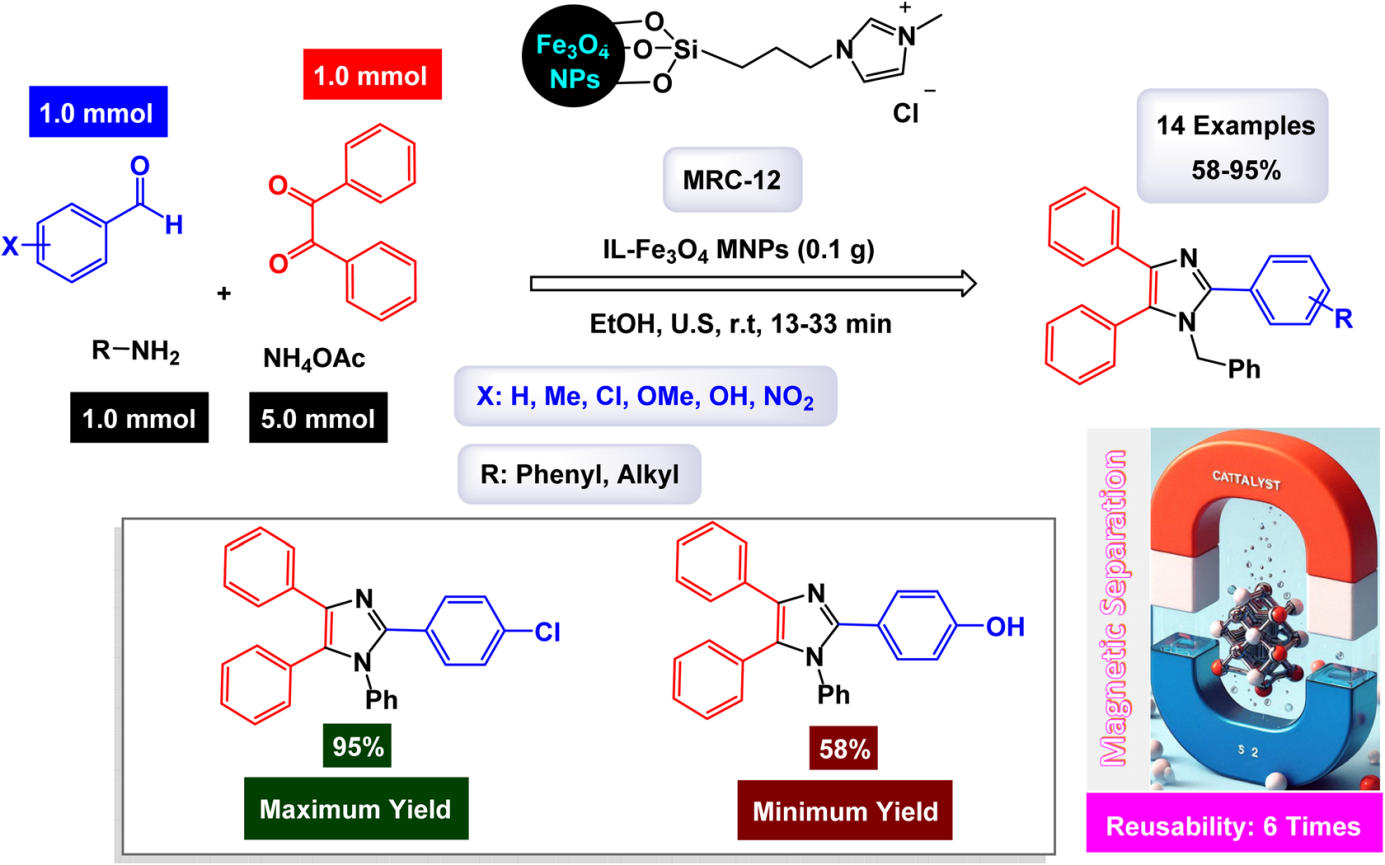
Synthesis of 1,2,4,5-tetrasubstituted imidazoles [catalysis by the IL-Fe_3_O_4_ MNPs nanomaterial].

Safari and Zarnegar developed a novel ionic liquid, specifically 1-methyl-3-(3-trimethoxysilylpropyl)imidazolium chloride, which is utilized in conjunction with magnetite nanoparticles (Fe_3_O_4_) to create a highly effective magnetically recoverable catalyst denoted as IL-Fe_3_O_4_ MNPs [MRC-20]. This innovative catalyst facilitates the one-pot synthesis of 1,2,4,5-tetrasubstituted imidazoles through an efficient multicomponent reaction process conducted in ethanol under ultrasonic conditions, as illustrated in Scheme 18.^131^ The structural characteristics of the catalyst were analyzed using SEM and TEM images, which revealed that the magnetite Fe_3_O_4_ nanoparticles possess an average diameter of approximately 18 nm and exhibit a nearly spherical morphology. Additionally, VSM analysis demonstrated that the IL-Fe_3_O_4_ MNP nanocatalyst [MRC-12] exhibits significantly high magnetic properties. The results of this study further highlighted that the incorporation of electron-withdrawing groups on the aromatic aldehydes and primary amines played a crucial role in enhancing the yields of the targeted 1,2,4,5-tetrasubstituted imidazoles, underscoring the effectiveness of this catalytic system. The tests conducted on the IL-Fe_3_O_4_ MNP nanocatalyst [MRC-12] demonstrated its remarkable stability, and that the catalytic efficiency was retained with only minimal decline after being utilized six times.

**Scheme 19:**
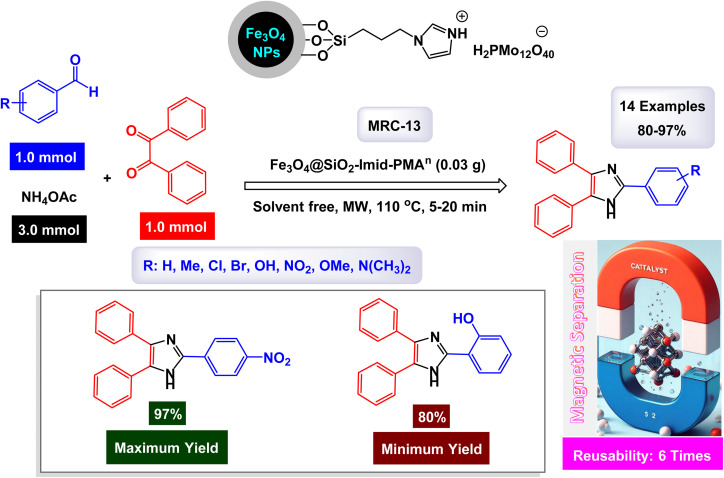
Synthesis of triaryl imidazoles [catalysis by the Fe_3_O_4_@SiO_2_-imid-PMA^*n*^ nanocomposite].

**Scheme 20:**
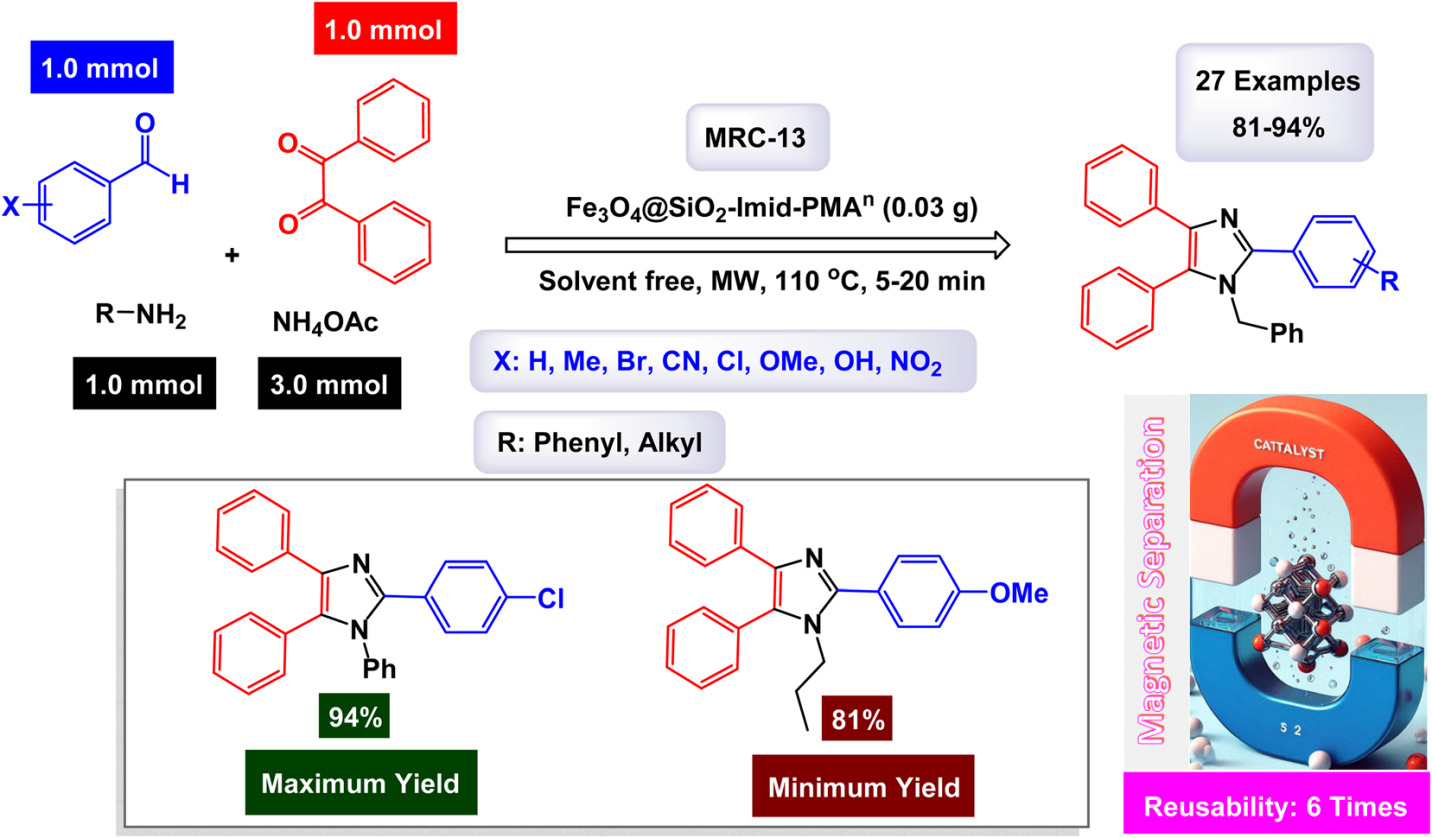
Synthesis of 1,2,4,5-tetrasubstituted imidazoles [catalysis by the Fe_3_O_4_@SiO_2_-imid-PMA^*n*^ nanocomposite].

**Scheme 21:**
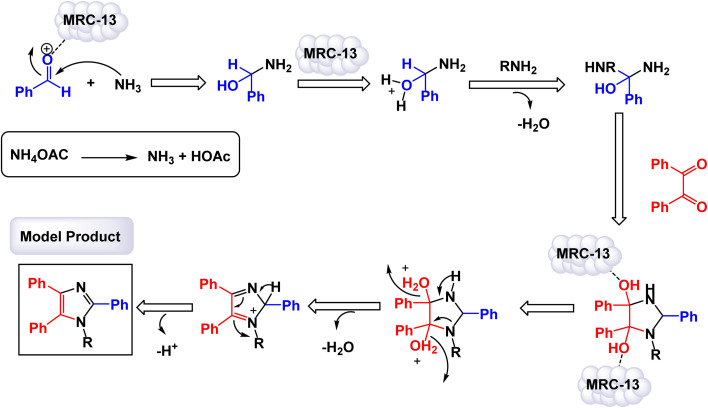
Plausible mechanism for synthesis of 1,2,4,5-tetrasubstituted imidazoles [catalysis by the Fe_3_O_4_@SiO_2_-imid-PMA^*n*^ nanocomposite].

The research team led by Esmaeilpour successfully developed an innovative and environmentally friendly procedure utilizing Fe_3_O_4_@SiO_2_-imid-PMA^*n*^ as a magnetic catalyst [MRC-13]. This catalyst facilitates the rapid and efficient synthesis of complex organic compounds, specifically 2,4,5-trisubstituted and 1,2,4,5-tetrasubstituted imidazoles, all accomplished under solvent-free conditions and with the application of microwave irradiation.^132^ The process yields impressive results, delivering high-quality products in excellent yields. Characterization of the Fe_3_O_4_@SiO_2_-imid-PMA^*n*^ nanoparticles *via* SEM and TEM demonstrated their spherical morphology, with an average diameter of approximately 50 nanometers. Further analyses, including TGA and XRD, confirmed the thermal stability and structural properties of the nanocatalyst. The intricate details of the multicomponent synthesis process for both 2,4,5-trisubstituted and 1,2,4,5-tetrasubstituted imidazoles are visually presented in Schemes 19 and 20. According to the proposed mechanism outlined in Scheme 21, the process begins with the protonation of the carbonyl group in aryl aldehyde. This step is followed by a nucleophilic attack from nitrogen atoms derived from ammonia (generated from NH_4_OAc) and aniline, leading to the formation of a reaction intermediate. In the presence of the Fe_3_O_4_@SiO_2_-imid-PMA^*n*^ catalyst, this intermediate then condenses with benzil, ultimately evolving into tetrasubstituted imidazoles through a dehydration process. H_3_PMo_12_O_40_ (PMA) likely serves as the active site responsible for activating the carbonyl group in the aldehyde, thereby facilitating the subsequent steps of the imidazole synthesis. The [MRC-21] catalyst was tested over six consecutive cycles without any significant loss in catalytic performance.

In the mechanism presented in Scheme 21 for the synthesis of 1,2,4,5-tetrasubstituted imidazoles, the Fe_3_O_4_@SiO_2_-imid-PMA nanocomposite catalyst plays a pivotal role. It significantly enhances the efficiency and selectivity of the reaction process. The initial step involves the interaction between an aromatic aldehyde and an amine, which are essential substrates for forming the desired imidazole derivatives. When these substrates come into contact with the catalyst, the unique properties of Fe_3_O_4_@SiO_2_-imid-PMA facilitate their activation. The catalyst acts by coordinating with the aromatic aldehyde, thereby lowering its activation energy. This coordination also plays a crucial role in promoting a favorable orientation for nucleophilic attack by the amine. This interaction leads to the formation of a key intermediate that is stabilized by the catalyst. As the reaction progresses, this intermediate undergoes further transformations, including condensation and cyclization steps that ultimately yield the tetrasubstituted imidazole product. Throughout this mechanism, Fe_3_O_4_@SiO_2_-imid-PMA not only stabilizes reactive intermediates but also enhances electron transfer processes necessary for bond formation. The presence of functional groups within the nanocomposite contributes to its ability to interact effectively with both reactants and intermediates, ensuring that each step of the reaction proceeds smoothly.

Moreover, one of the significant advantages of utilizing this particular catalyst is its recyclability. After completing a reaction cycle, Fe_3_O_4_@SiO_2_-imid-PMA can be easily separated from the product mixture and reused in subsequent reactions without a notable decrease in catalytic activity. This feature not only promotes economic efficiency but also aligns with contemporary sustainable practices in organic synthesis, a fact that chemists, researchers, and students in the field will surely appreciate. The Fe_3_O_4_@SiO_2_-imid-PMA nanocomposite serves as a vital component in facilitating the synthesis of 1,2,4,5-tetrasubstituted imidazoles by lowering activation barriers and stabilizing intermediates throughout the mechanism. Its ability to enhance reactivity while remaining recoverable underscores its importance in advancing efficient and sustainable methodologies within organic chemistry.

### 2.4. Catalysis by magnetic nanoparticles modified with acidic catalysts

**Scheme 22:**
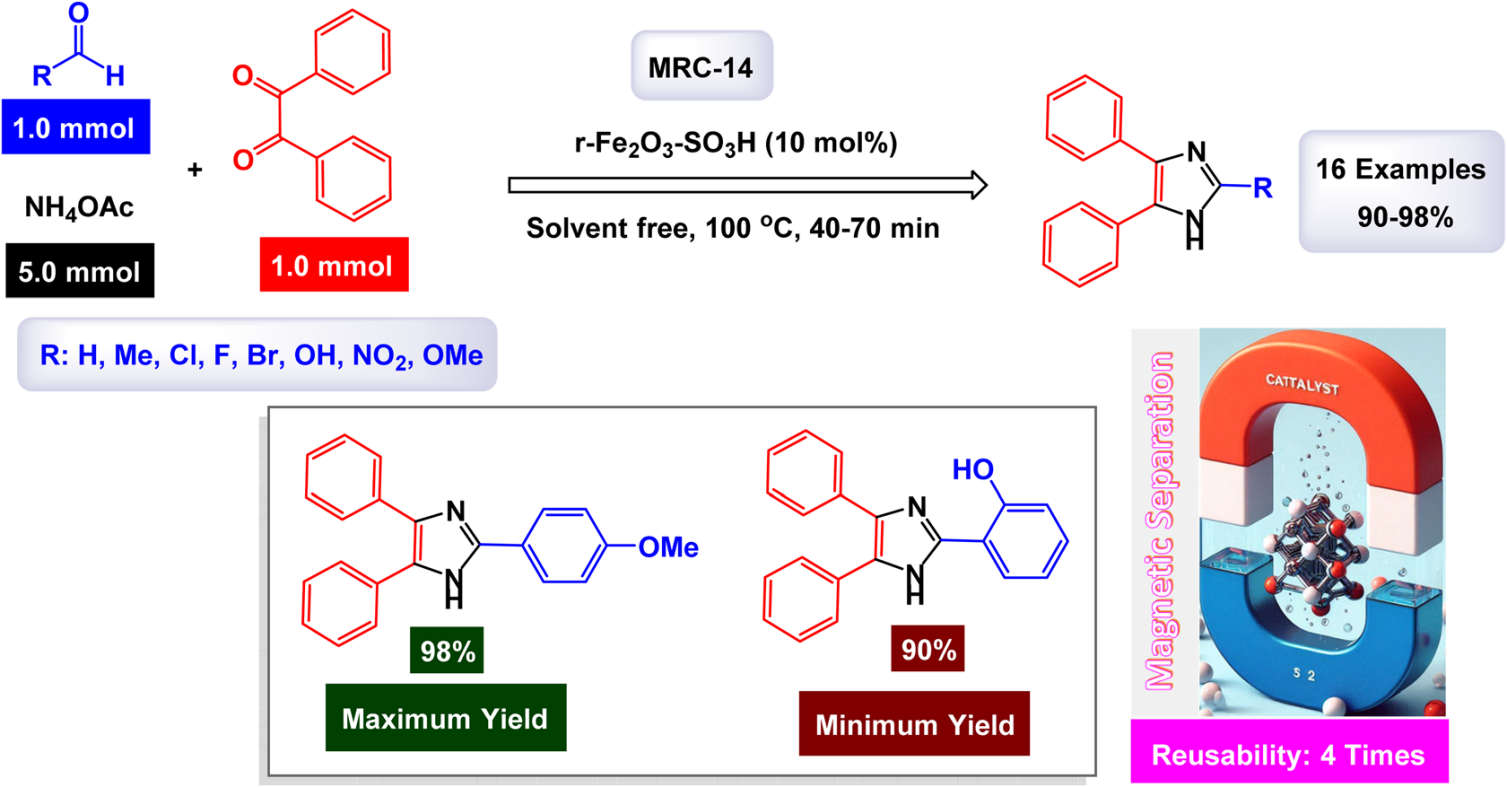
Synthesis of triaryl imidazoles [catalysis by the γ-Fe_2_O_3_-SO_3_H nanocomposite].

**Scheme 23:**
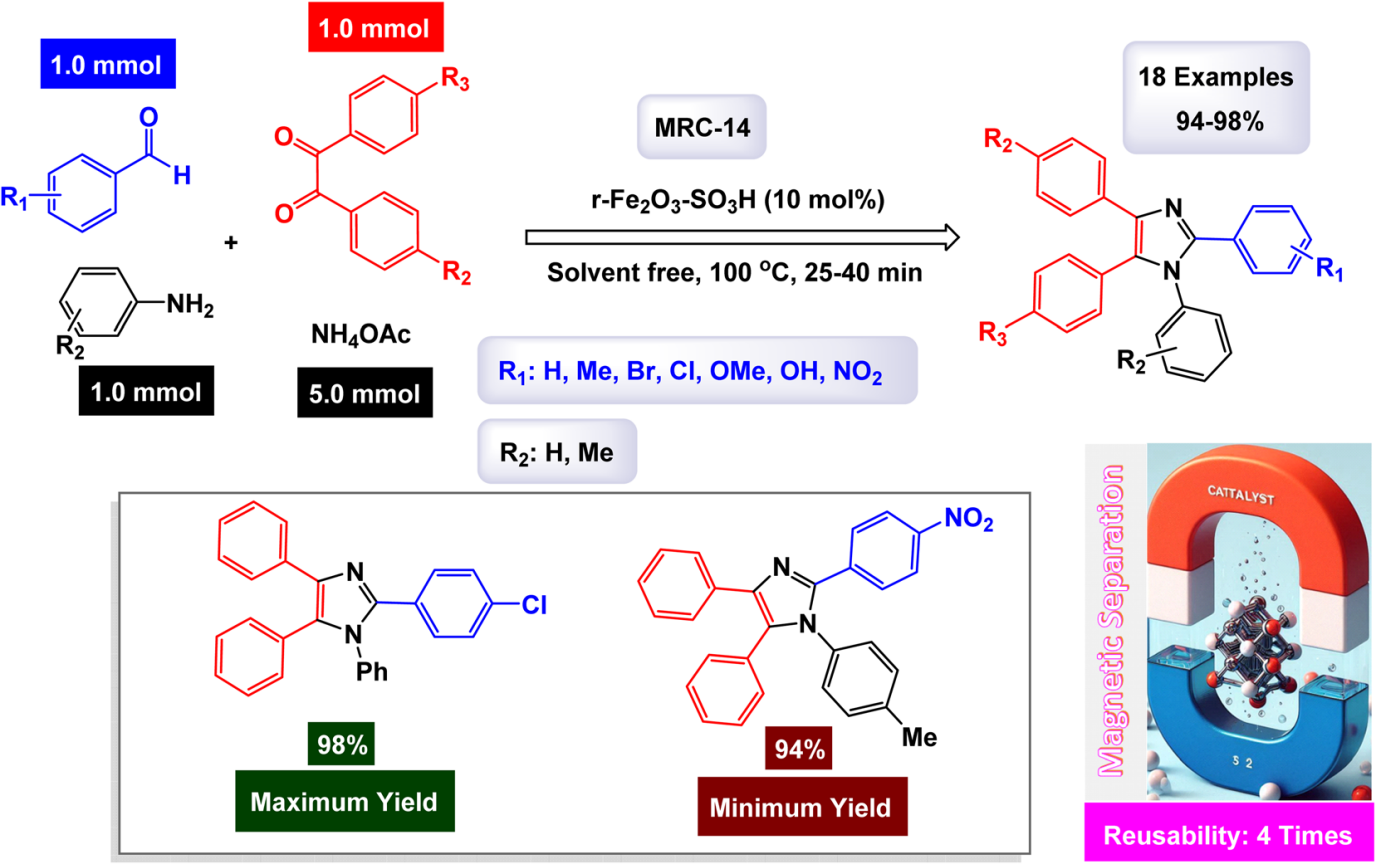
Synthesis of 1,2,4,5-tetrasubstitutedimidazoles [catalysis by the γ-Fe_2_O_3_-SO_3_H nanocomposite].

**Scheme 24:**
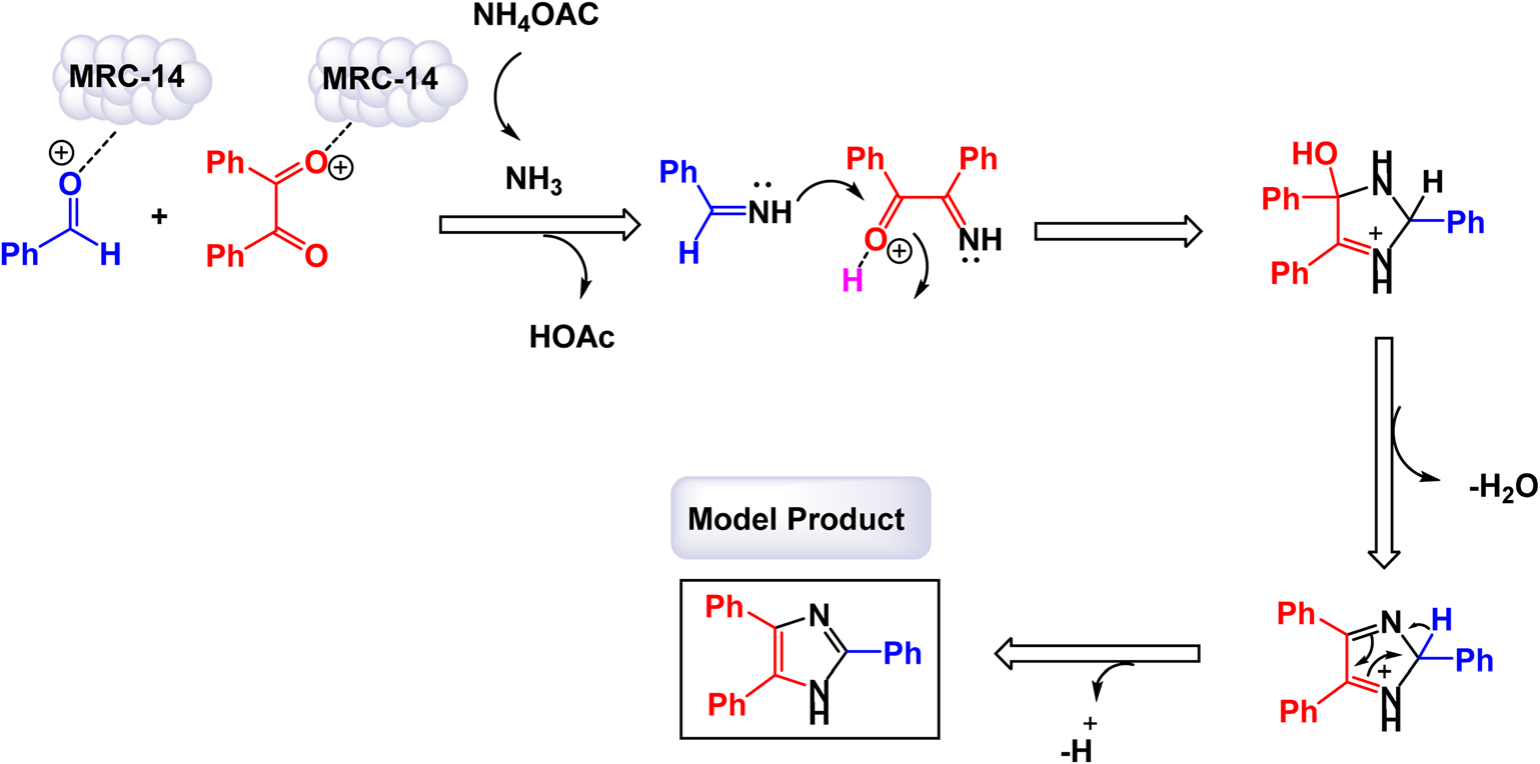
Plausible mechanism for synthesis of triaryl imidazoles [catalysis by the γ-Fe_2_O_3_-SO_3_H nanocomposite].

Sulfonic and sulfamic acids, as a strong and efficient catalyst in various chemical reactions, play an important role in improving industrial processes and reducing costs.^133,134^ The research group led by Sakhdari has made significant advancements in the field of catalysis by utilizing magnetic nanoparticle-supported sulfonic acid (γ-Fe_2_O_3_-SO_3_H) [MRC-14]. This innovative catalyst demonstrates remarkable efficiency in synthesizing various imidazole compounds, specifically 2,4,5-trisubstituted and 1,2,4,5-tetrasubstituted imidazoles.^135^ The synthesis process is notably swift, requiring only 40 to 70 minutes for the trisubstituted variants and approximately 30 to 40 minutes for the tetrasubstituted forms, all while yielding high-purity products-between 92% and 98% for the trisubstituted imidazoles and 94% to 98% for their tetrasubstituted counterparts (Schemes 22 and 23). The structural integrity of these synthesized products was confirmed through advanced analytical techniques, including FT-IR and NMR. This method of synthesis is particularly favorable due to its adherence to green chemistry principles, eco-friendly processes, and solvent-free conditions. Several key advantages characterize this catalytic reaction. It features high catalytic activity, and significantly reduced reaction times, which enhances the overall efficiency of the process. The exceptional purity and high yields of the products further underscore the effectiveness of the γ-Fe_2_O_3_-SO_3_H catalyst. The catalytic mechanism involves the activation of aldehydes and 1,2-diketones by the γ-Fe_2_O_3_-SO_3_H acidic catalyst. This activation allows for interaction with ammonium acetate, leading to the formation of imine intermediates. Subsequently, the nitrogen atom of the imine intermediate engages in a reaction with the positively charged carbonyl carbon in the corresponding 1,2-diketone imine intermediate to create a carbo-cation. The final steps involve dehydration and a sigmatropic shift, culminating in the production of the desired 2,4,5-trisubstituted imidazole. This comprehensive approach reveals not only the practicality of the method but also its potential impact in the field of synthetic chemistry (Scheme 24).

In the mechanism depicted in Scheme 24 for the synthesis of triaryl imidazoles, the γ-Fe_2_O_3_-SO_3_H nanocomposite catalyst plays a pivotal role in facilitating the reaction between MRC-14, an aromatic aldehyde, and ammonia. The process begins with the interaction of MRC-14 with the catalyst, which serves to activate the aldehyde through protonation or coordination to the acidic sites present on the surface of γ-Fe_2_O_3_-SO_3_H. This activation not only enhances the electrophilicity of the carbonyl carbon, making it more susceptible to nucleophilic attack by ammonia, but also does so with remarkable efficiency. As ammonia approaches the activated aldehyde, it forms a tetrahedral intermediate that is stabilized by interactions with the catalyst. This stabilization is crucial as it lowers the energy barrier for the transition state, leading to product formation. Following this step, dehydration occurs, eliminating water and resulting in the formation of an imine intermediate. The catalyst's acidic environment promotes this dehydration step, ensuring that it proceeds efficiently.

The next phase involves cyclization, where another equivalent of MRC-14 can participate similarly. The catalyst again facilitates this process by stabilizing an additional intermediate formed during cyclization. The presence of multiple reactive sites on both MRC-14 and ammonia allows for further reactions that lead to the formation of triaryl imidazole products. Throughout these steps, γ-Fe_2_O_3_-SO_3_H not only enhances reactivity but also ensures selectivity towards desired products by providing a controlled environment that minimizes side reactions. This high level of selectivity provides reassurance about the precision of the process, contributing to the audience's confidence in the catalyst's performance. Furthermore, after completing its catalytic cycle, this nanocomposite can be easily separated from the reaction mixture and reused without significant loss in activity or selectivity, thus contributing to both economic viability and sustainability in synthetic methodologies. The γ-Fe_2_O_3_-SO_3_H nanocomposite acts as a vital facilitator in the synthesis of triaryl imidazoles by activating substrates and stabilizing intermediates throughout various stages of the mechanism. Its ability to enhance reaction efficiency while remaining recoverable underscores its importance in advancing practical applications within organic synthesis. This potential for practical applications inspires confidence in the research's impact on the field of organic synthesis.

**Scheme 25:**
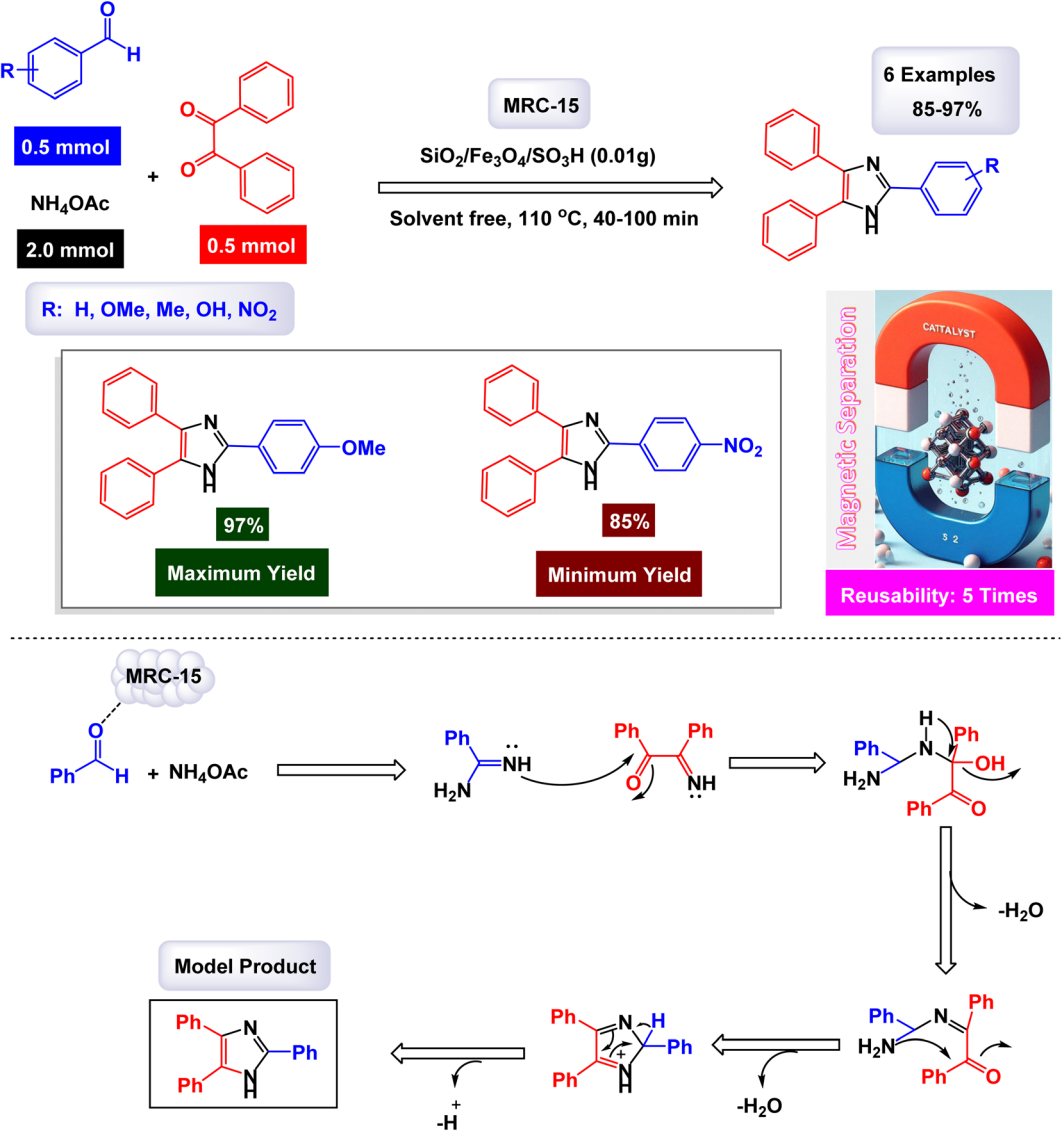
Synthesis of triaryl imidazoles [catalysis by the SiO_2_/Fe_3_O_4_/SO_3_H nanocomposite].

Saadatjoo and Basooti developed an innovative nanomagnetic acid catalyst, denoted as SiO_2_/Fe_3_O_4_/SO_3_H, through a straightforward and efficient synthesis method.^136^ This catalyst was employed to facilitate the production of 2,4,5-trisubstituted imidazoles by strategically combining three components: benzil, various aldehydes, and ammonium acetate, which served as the ammonia source essential for the reaction. In this catalyst, the acidic SO_3_H groups on the surface of the SiO_2_/Fe_3_O_4_/SO_3_H catalyst activate the carbonyl group of the aldehyde, facilitating nucleophilic attack by ammonia to form a diamine intermediate, which subsequently condenses with the ketone to yield imidazole derivatives (Scheme 25). The structural analysis of the catalyst through SEM and TEM revealed that it is composed of spherical silica particles measuring approximately 98 nanometers in diameter, interspersed with magnetic particles that are about 9 nanometers in size. This unique composition contributes to the catalyst's exceptional properties. Further evaluations conducted using VSM and TGA confirmed that the SiO_2_/Fe_3_O_4_/SO_3_H catalyst possesses remarkable magnetic characteristics and impressive thermal stability.

In the mechanism illustrated in Scheme 25 for the synthesis of triaryl imidazoles, the SiO_2_/Fe_2_O_3_/SO_3_H nanocomposite catalyst emerges as a key player, significantly promoting the reaction between MRC-15, an aromatic aldehyde, and ammonium acetate. The process initiates with the activation of MRC-15 through interaction with the acidic sites on the catalyst. This interaction enhances the electrophilicity of the carbonyl carbon, making it more reactive and susceptible to nucleophilic attack by ammonia derived from ammonium acetate. As ammonia approaches the activated aldehyde, a tetrahedral intermediate is formed. The catalyst stabilizes this intermediate, which is crucial for lowering the activation energy required for subsequent steps in the reaction pathway. Following this formation, a dehydration step occurs where water is eliminated, leading to the generation of an imine intermediate. The acidic environment provided by the SiO_2_/Fe_2_O_3_/SO_3_H catalyst facilitates this dehydration effectively, ensuring that it proceeds smoothly and efficiently.

The mechanism continues with cyclization, during which another equivalent of MRC-15 can react similarly. The catalyst, with its stabilizing effect, not only promotes further reactions but also guides them with precision toward forming the desired triaryl imidazole products. This role of the catalyst in guiding reactions adds a layer of control to the process, enhancing the audience's understanding of the mechanism. Throughout these stages, SiO_2_/Fe_2_O_3_/SO_3_H enhances both reactivity and selectivity by creating an environment that minimizes side reactions while favoring product formation. Additionally, one of the significant advantages of using this nanocomposite catalyst is its recyclability, a feature that not only underscores its practicality but also aligns well with sustainable practices in organic synthesis. After completing a catalytic cycle, it can be easily separated from the reaction mixture and reused without substantial loss of activity or selectivity. The SiO_2_/Fe_2_O_3_/SO_3_H nanocomposite serves as a vital facilitator in the synthesis of triaryl imidazoles by activating substrates and stabilizing key intermediates throughout various stages of the mechanism. Its ability to enhance reaction efficiency while remaining recoverable underscores its importance in advancing practical applications within organic chemistry.

In addition to its outstanding properties, the effectiveness of the SiO_2_/Fe_3_O_4_/SO_3_H nanocatalyst, referred to as MRC-15, was rigorously tested for its recyclability. The results demonstrated its significant durability; even after being utilized in five consecutive reactions, and the catalyst retained its catalytic performance with only a minimal reduction in efficiency.

**Scheme 26:**
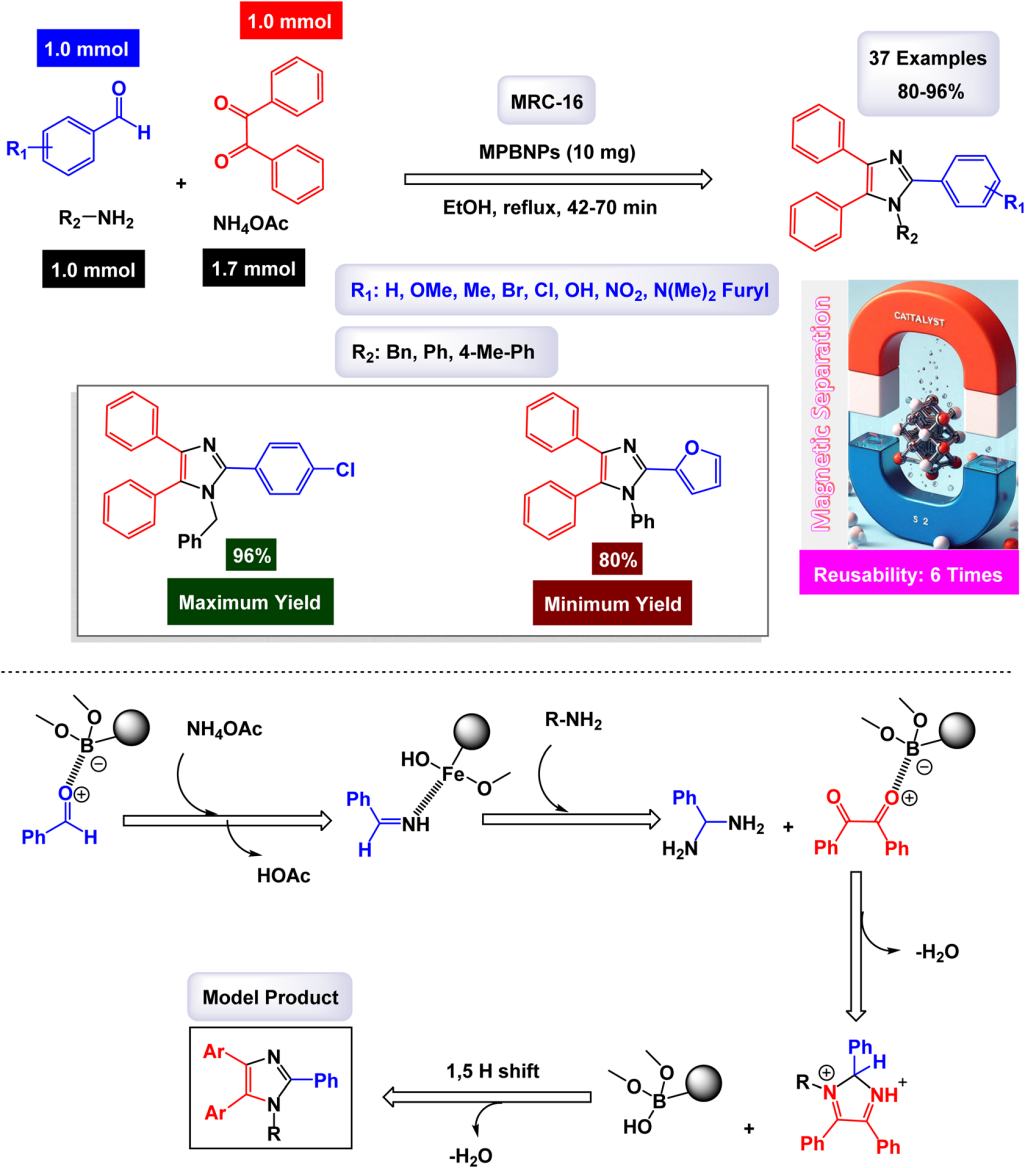
Synthesis of 1,2,4,5-tetrasubstituted imidazoles [catalysis by the MPBNP nanocomposite].

The research team led by Dekamin synthesized magnetic polyborate nanoparticles (MRC-8) from boric acid using a simple ball-milling procedure.^137^ The nanoparticles were thoroughly characterized through techniques such as FT-IR, EDX, XRD, FESEM, VSM, and TGA. MRC-8 nanoparticles were then assessed for their catalytic efficiency in producing tetra-substituted imidazoles *via* cascade cyclocondensation. They also exhibited remarkable performance in the *in situ* air oxidation of benzil or benzoin, along with aromatic aldehydes, primary amines, and ammonium acetate in ethanol under reflux conditions, showcasing their potential as green catalysts in sustainable chemistry. The tests conducted on the MPBNP nanocatalyst [MRC-16] for recycling demonstrated its remarkable stability, and that the catalytic efficiency was retained with only minimal decline after being utilized six times. The proposed mechanism describes how the carbonyl groups of aldehydes become more electrophilic when interacting with the Lewis acidic centers in B and Fe atoms of the MPBNP catalyst. This interaction allows nucleophiles like ammonia and amines to add to the activated carbonyl, forming imine and aminal intermediates. These intermediates then react with activated carbonyl groups in benzil or benzoin, resulting in a cyclic intermediate. This step involves the elimination of a water molecule through imine condensation and subsequent air oxidation in the case of benzoin. Ultimately, the desired imidazole derivatives are produced after a [1,5-H] shift, enabling the regenerated MPBNPs to continue their catalytic cycle (Scheme 26).

In the mechanism depicted in Scheme 26 for the synthesis of 1,2,4,5-tetrasubstituted imidazoles, the MPBNP catalyst (magnetite–polyaniline-based nanocomposite) plays a crucial role in facilitating the reaction between ammonium acetate (NH_4_OAc) and an aromatic amine (R-NH_2_). The process begins with the activation of ammonium acetate by the acidic sites present on the surface of the MPBNP catalyst. This activation enhances the electrophilicity of the nitrogen atom in NH_4_OAc, making it more reactive towards nucleophilic attack. As the reaction progresses, the activated ammonium acetate reacts with the aromatic amine to form an intermediate that is stabilized by interactions with the catalyst. The presence of MPBNPs not only lowers the activation energy required for this step but also promotes a favorable orientation for the reactants, thereby increasing the likelihood of successful collisions and product formation. Following this initial interaction, a dehydration step occurs where water is eliminated from the intermediate, leading to the formation of an imine-like species.

The next phase involves cyclization, where another equivalent of R-NH_2_ can participate in forming a cyclic structure. Here again, MPBNPs play a pivotal role by stabilizing transition states and intermediates during this cyclization process. This stabilization is essential as it allows for a more efficient pathway toward forming tetrasubstituted imidazole products. Additionally, after cyclization, a 1,5-H shift may occur, which further contributes to product diversity and complexity. Throughout these steps, MPBNPs enhance both reactivity and selectivity by creating an environment that minimizes side reactions while favoring the formation of desired products. Furthermore, one notable advantage of using this nanocomposite catalyst is its magnetic properties; after completing its catalytic cycle, it can be easily separated from the reaction mixture using an external magnet. This feature not only simplifies product isolation but also supports sustainability by allowing for catalyst reuse without significant loss of activity. MPBNPs serve as a vital facilitator in the synthesis of 1,2,4,5-tetrasubstituted imidazoles by activating substrates and stabilizing key intermediates throughout various stages of the mechanism. Its ability to enhance reaction efficiency while remaining recoverable underscores its importance in advancing practical applications within organic synthesis and catalysis.

**Scheme 27:**
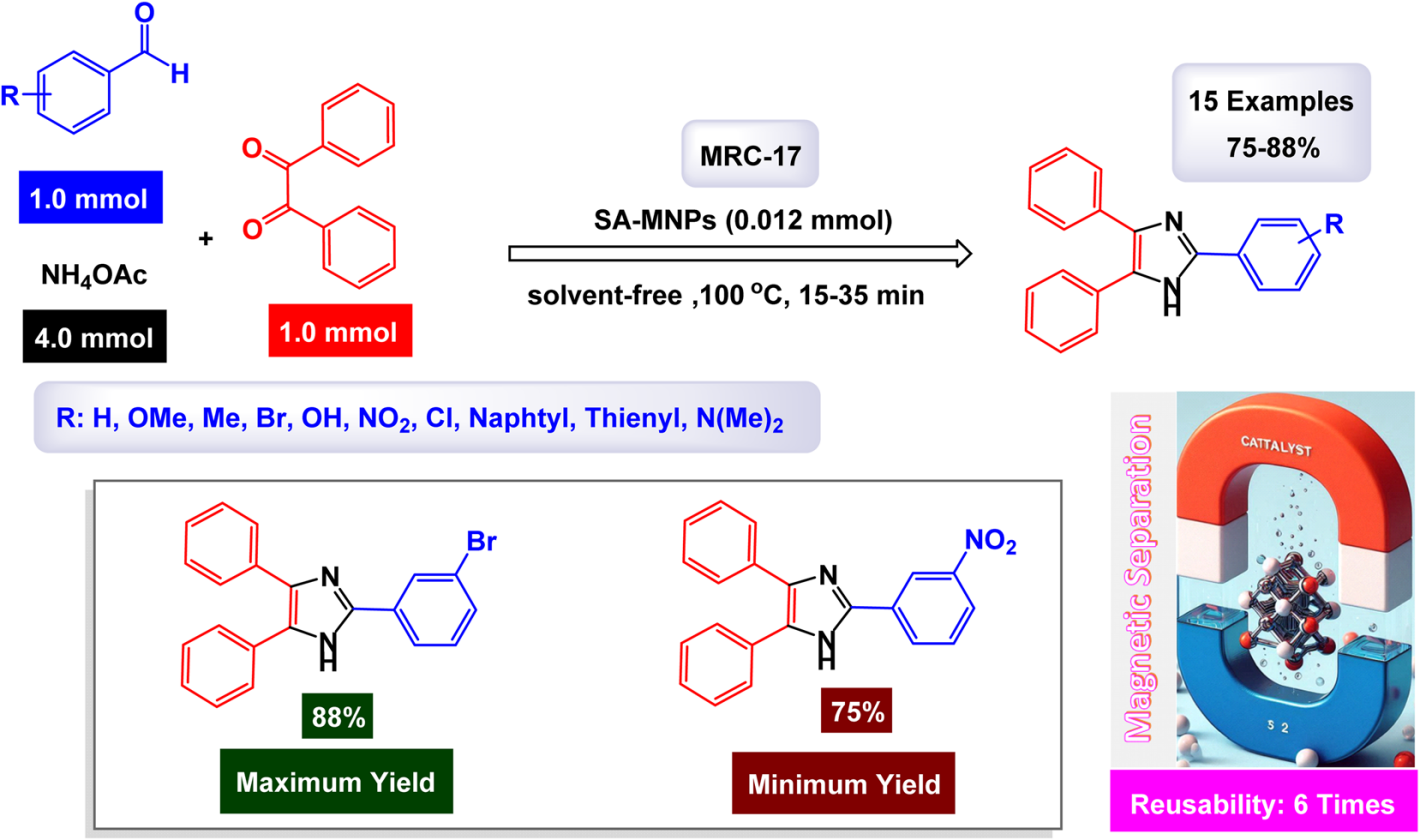
Synthesis of triaryl imidazoles [catalysis by the SA-MNP nanocomposite].

In a notable approach for synthesizing triaryl imidazoles, Safari and Zarnegar detailed a method involving the preparation of iron oxide (Fe_3_O_4_) nanoparticles through a chemical coprecipitation technique.^138^ Following this, the nanoparticles were treated with 3-aminopropyltriethoxysilane (APTES) in a silanization reaction, which served to functionalize their surface with amino groups. This modification was further advanced by grafting chlorosulfuric acid onto the amino-functionalized Fe_3_O_4_ nanoparticles, resulting in the formation of sulfamic acid-functionalized magnetic nanoparticles (SA-MNPs) [MRC-17]. These nanoparticles showed impressive effectiveness as a solid acid catalyst, facilitating the successful one-pot synthesis of 2,4,5-trisubstituted imidazoles *via* a three-component reaction under ultrasound irradiation (Scheme 27). This innovative method significantly reduced reaction times while achieving impressive yields of the desired imidazoles. SEM images reveal that the SA-MNPs possess a nearly spherical morphology, with sizes exceeding 20 nanometers, highlighting their suitability for catalysis in this reaction. The tests conducted on the SA-MNP nanocatalyst [MRC-17] for recycling demonstrated remarkable stability, and that the catalytic efficiency was retained with only minimal decline after being utilized six times.

### 2.5. Catalysis by magnetic nanoparticles modified with copper catalysts

Copper serves as a flexible catalyst in chemical reactions, mainly because of its advantageous redox potential and the ability to exist in various oxidation states, which boosts its reactivity and efficiency.^12,144^ It is instrumental in coupling reactions, facilitating mechanisms involving one or two electrons.^145^ Employing copper also corresponds to the tenets of green chemistry, providing a more sustainable option than other metals. The coordination of copper with heteroatoms and π-bonds, along with its activation of terminal alkynes, makes it an invaluable tool in organic synthesis.^146–148^

**Scheme 28:**
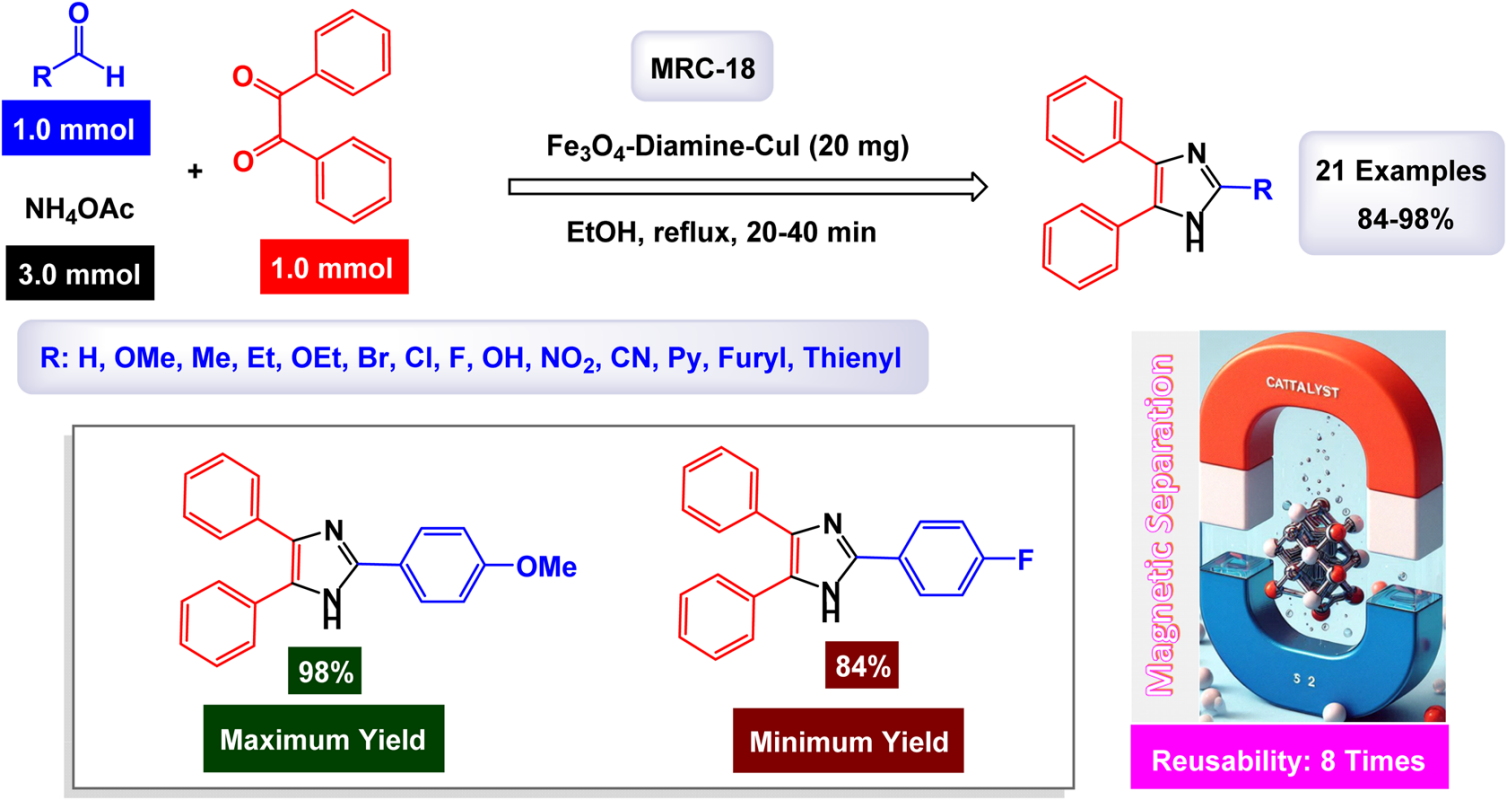
Synthesis of triaryl imidazoles [catalysis by the Fe_3_O_4_-diamine-CuI nanocomposite].

**Scheme 29:**
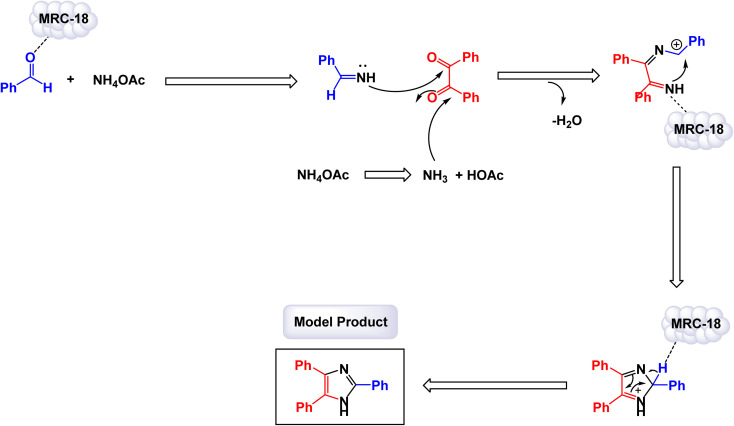
Plausible mechanism for synthesis of triaryl imidazoles [catalysis by the Fe_3_O_4_-diamine-CuI nanocomposite (MRC-18)].

In a noteworthy study, Younes and his research team introduced an innovative approach utilizing copper(i) iodide, which is supported on the surface of magnetic Fe_3_O_4_ nanoparticles modified with 3,4-diaminobenzoic acid. This novel nanocatalyst, denoted as Fe_3_O_4_@Diamine-CuI, has shown remarkable efficiency in facilitating the synthesis of 2,4,5-trisubstituted imidazoles.^139^ The reaction occurs through a one-pot three-component process involving aromatic aldehydes, benzil, and ammonium acetate as the nitrogen source, all performed under reflux conditions in water (as illustrated in Scheme 28). The characterization of the Fe_3_O_4_@Diamine-CuI nanomaterial [MRC-18] is comprehensive, employing a variety of analytical techniques, including FT-IR spectroscopy, SEM, energy-dispersive EDX, TEM, XRD, VSM, EDX elemental mapping, and ICP-OES. The findings reveal that the average particle sizes of the raw Fe_3_O_4_ nanoparticles and the modified Fe_3_O_4_@Diamine-CuI are approximately 17.23 nm and 25.71 nm, respectively, indicating a successful modification. This catalytic system is notably effective under eco-friendly conditions and is cost-effective. Moreover, it requires only a short reaction time, utilizes water as the solvent, and ensures simple purification of the resulting products. The proposed mechanism for the synthesis of 2,4,5-trisubstituted imidazoles catalyzed by Fe_3_O_4_@Diamine-CuI is depicted in Scheme 29, wherein the copper center (Cu^+^) serves as the active catalytic site, activating the aldehyde's carbonyl group and promoting condensation with ammonia and benzil through the formation of a diamine intermediate that subsequently leads to the desired imidazole derivatives.

In the mechanism illustrated in Scheme 29 for the synthesis of triaryl imidazoles, the Fe_3_O_4_-diamine-Cu nanocomposite catalyst plays a pivotal role in facilitating the reaction between MRC-18, an aromatic aldehyde, and ammonium acetate (NH_4_OAc). The process initiates with the activation of MRC-18 through interactions with the Lewis acidic sites provided by the copper component of the catalyst. This interaction enhances the electrophilicity of the carbonyl carbon in MRC-18, making it more susceptible to nucleophilic attack due to ammonia released from ammonium acetate. As ammonia approaches the activated aldehyde, a tetrahedral intermediate is formed. The presence of the Fe_3_O_4_-diamine-Cu catalyst stabilizes this intermediate, which is crucial for lowering the activation energy required for subsequent steps in the reaction pathway. Following this formation, a dehydration step occurs where water is eliminated, yielding an imine intermediate. The acidic environment created by the catalyst facilitates this dehydration effectively, ensuring that it proceeds smoothly and efficiently.

The mechanism continues with cyclization, during which another equivalent of MRC-18 can react similarly. Here again, the catalyst plays a critical role by stabilizing intermediates formed during this cyclization process. This stabilization not only promotes further reactions but also helps guide them toward forming the desired triaryl imidazole products. Throughout these stages, Fe_3_O_4_-diamine-Cu enhances both reactivity and selectivity by creating an environment that minimizes side reactions while favoring product formation. Additionally, one significant advantage of using this nanocomposite catalyst is its magnetic properties; after completing a catalytic cycle, it can be easily separated from the reaction mixture using an external magnet. This feature aligns well with sustainable practices in organic synthesis. The Fe_3_O_4_-diamine-Cu nanocomposite serves as a vital facilitator in the synthesis of triaryl imidazoles by activating substrates and stabilizing key intermediates throughout various stages of the mechanism. Its ability to enhance reaction efficiency while remaining recoverable underscores its importance in advancing practical applications within organic chemistry and catalysis.

**Scheme 30:**
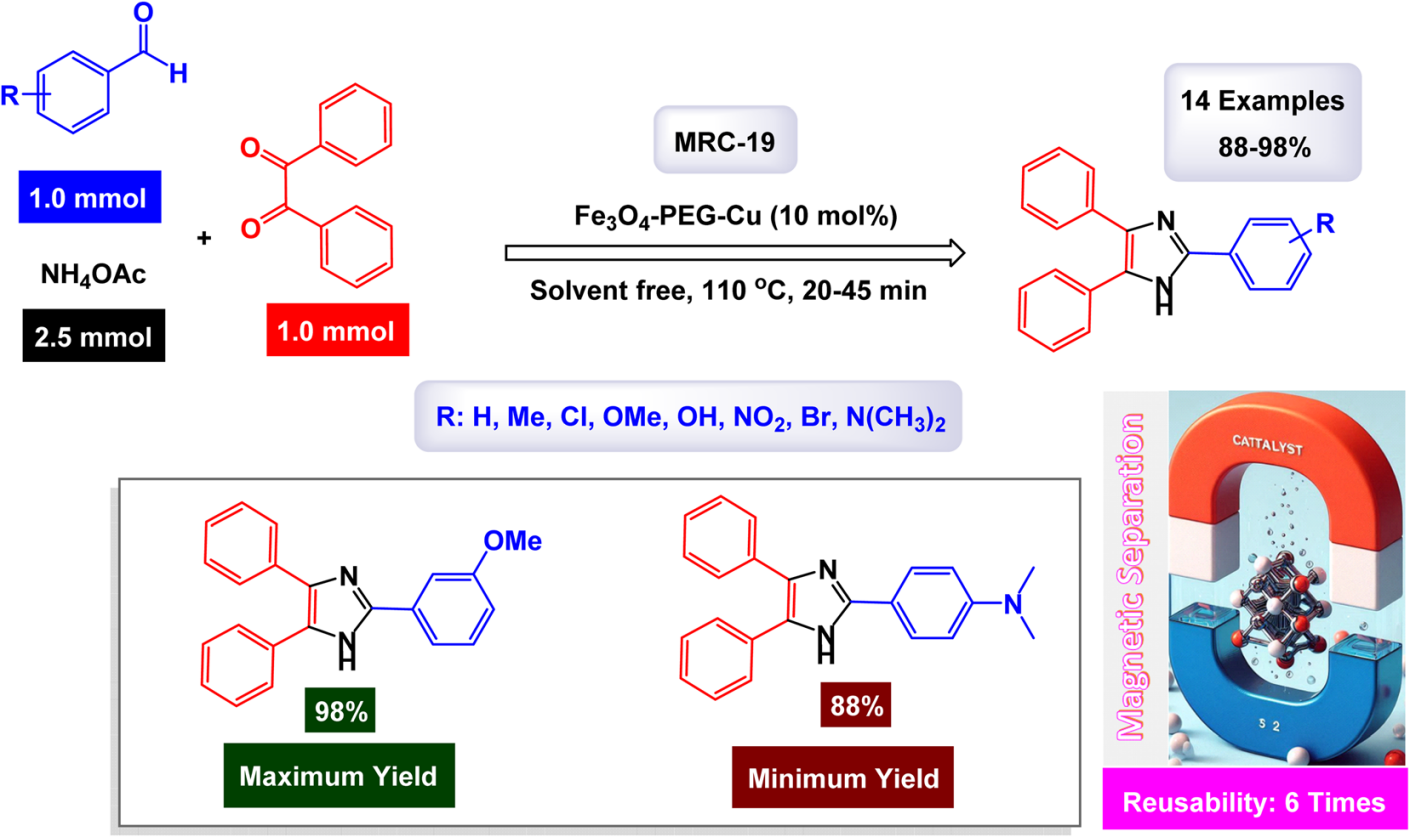
Synthesis of triaryl imidazoles [catalysis by the Fe_3_O_4_-PEG-Cu nanomaterial].

**Scheme 31:**
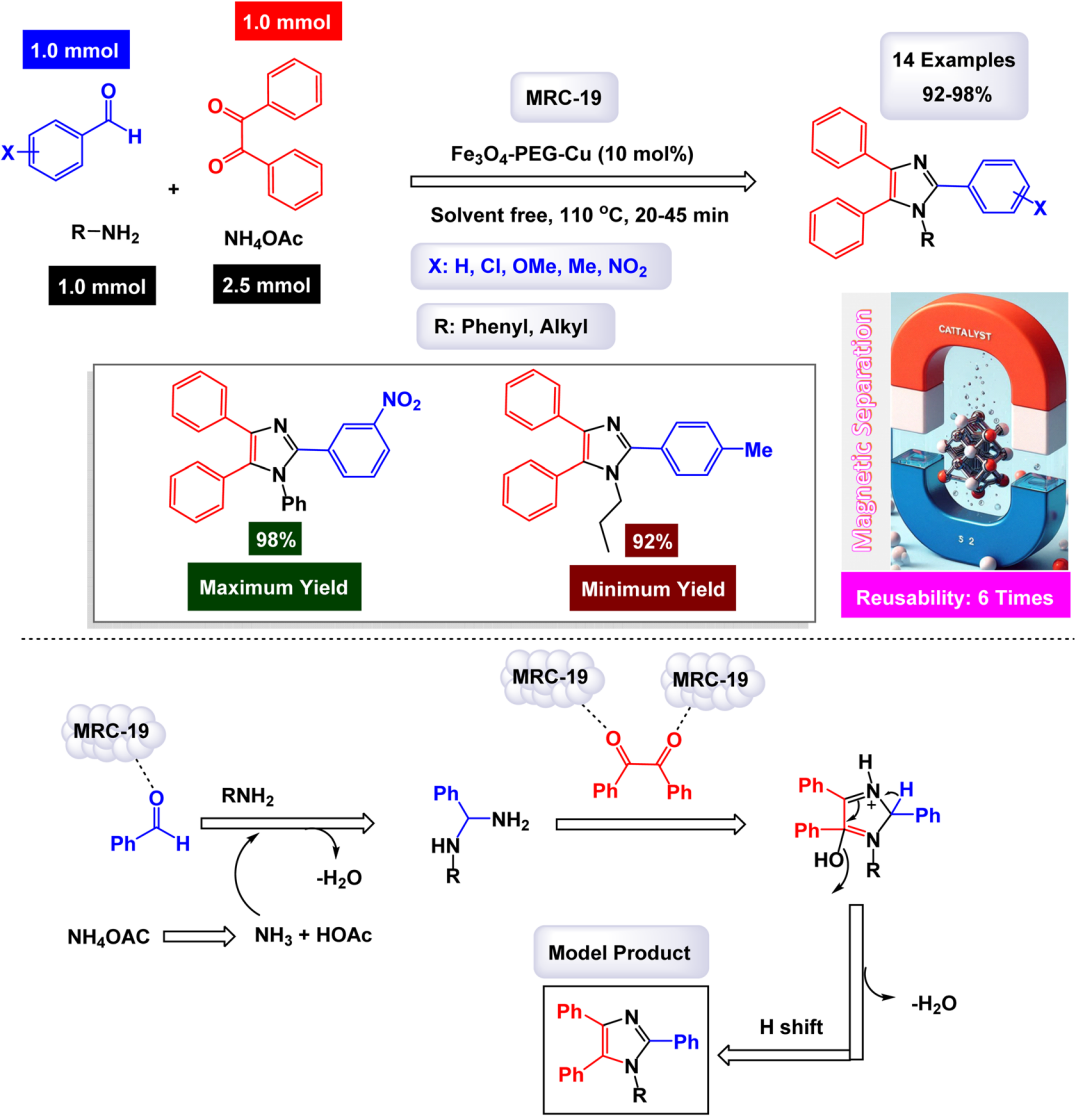
Synthesis of 1,2,4,5-tetrasubstituted imidazoles [catalysis by the Fe_3_O_4_-PEG-Cu nanomaterial (MCR-19)].

Safari and Zarnegar successfully developed a novel nanocomposite, Fe_3_O_4_-polyethylene glycol–Cu, abbreviated as the Fe_3_O_4_-PEG-Cu nanomaterial [MRC-20]. This advanced material was meticulously analyzed for its catalytic efficiency in synthesizing 2,4,5-trisubstituted imidazoles and 1,2,4,5-substituted imidazoles, specifically under solvent-free conditions, as illustrated in Schemes 30 and 31. To validate the structural integrity and properties of the Fe_3_O_4_-PEG-Cu nanomaterial, a comprehensive range of characterization techniques was employed, including FT-IR, XRD, SEM, EDX, TGA, AAS, and VSM.^140^ According to the proposed reaction mechanism depicted in Scheme 9, the catalytic process initiates with the formation of a diamine intermediate. This is accomplished through the activation of an aldehyde's carbonyl group, facilitated by the copper ions present on the surface of the nanocomposite. Following this, the newly formed diamine undergoes condensation with a 1,2-diketone. This reaction progresses through a dehydration step, leading to the rearrangement of the product into an imino intermediate, ultimately resulting in the desired imidazole compounds.

In the mechanism depicted in Scheme 31 for the synthesis of 1,2,4,5-tetrasubstituted imidazoles, the Fe_3_O_4_-PEG-Cu nanomaterial catalyst (MCR-19) plays a crucial role in facilitating the reaction between ammonium acetate (NH_4_OAc) and an aromatic amine (R-NH_2_). The process begins with the activation of NH_4_OAc through interactions with the copper component of the catalyst. This activation enhances the electrophilicity of the nitrogen atom in NH_4_OAc, making it more reactive towards nucleophilic attack by R-NH_2_. As R-NH_2_ approaches the activated ammonium acetate, a key intermediate is formed. The presence of MCR-19 stabilizes this intermediate through coordination interactions, which lowers the activation energy required for subsequent steps in the reaction pathway. Following this initial interaction, a dehydration step occurs where water is eliminated from the intermediate, leading to the formation of an imine-like species. The next phase involves cyclization, during which another equivalent of R-NH_2_ can participate in forming a cyclic structure. Here again, MCR-19 plays an essential role by stabilizing transition states and intermediates during this cyclization process. This stabilization is vital as it allows for a more efficient pathway toward forming tetrasubstituted imidazole products. Additionally, after cyclization, a 1,5-H shift may occur, further contributing to product diversity and complexity.

Throughout these steps, MCR-19 enhances both reactivity and selectivity by creating an environment that minimizes side reactions while favoring the formation of desired products. One notable advantage of using this nanocomposite catalyst is its magnetic properties; after completing its catalytic cycle, it can be easily separated from the reaction mixture using an external magnet. This feature simplifies product isolation and supports sustainability by allowing for catalyst reuse without significant loss of activity. The Fe_3_O_4_-PEG-Cu nanomaterial serves as a vital facilitator in the synthesis of 1,2,4,5-tetrasubstituted imidazoles by activating substrates and stabilizing key intermediates throughout various stages of the mechanism. Its ability to enhance reaction efficiency while remaining recoverable underscores its importance in advancing practical applications within organic synthesis and catalysis.

The tests conducted on the Fe_3_O_4_-PEG-Cu nanocatalyst [MRC-20] for recycling demonstrated its remarkable stability, and that the catalytic efficiency was retained with only minimal decline after being utilized six times.

**Scheme 32:**
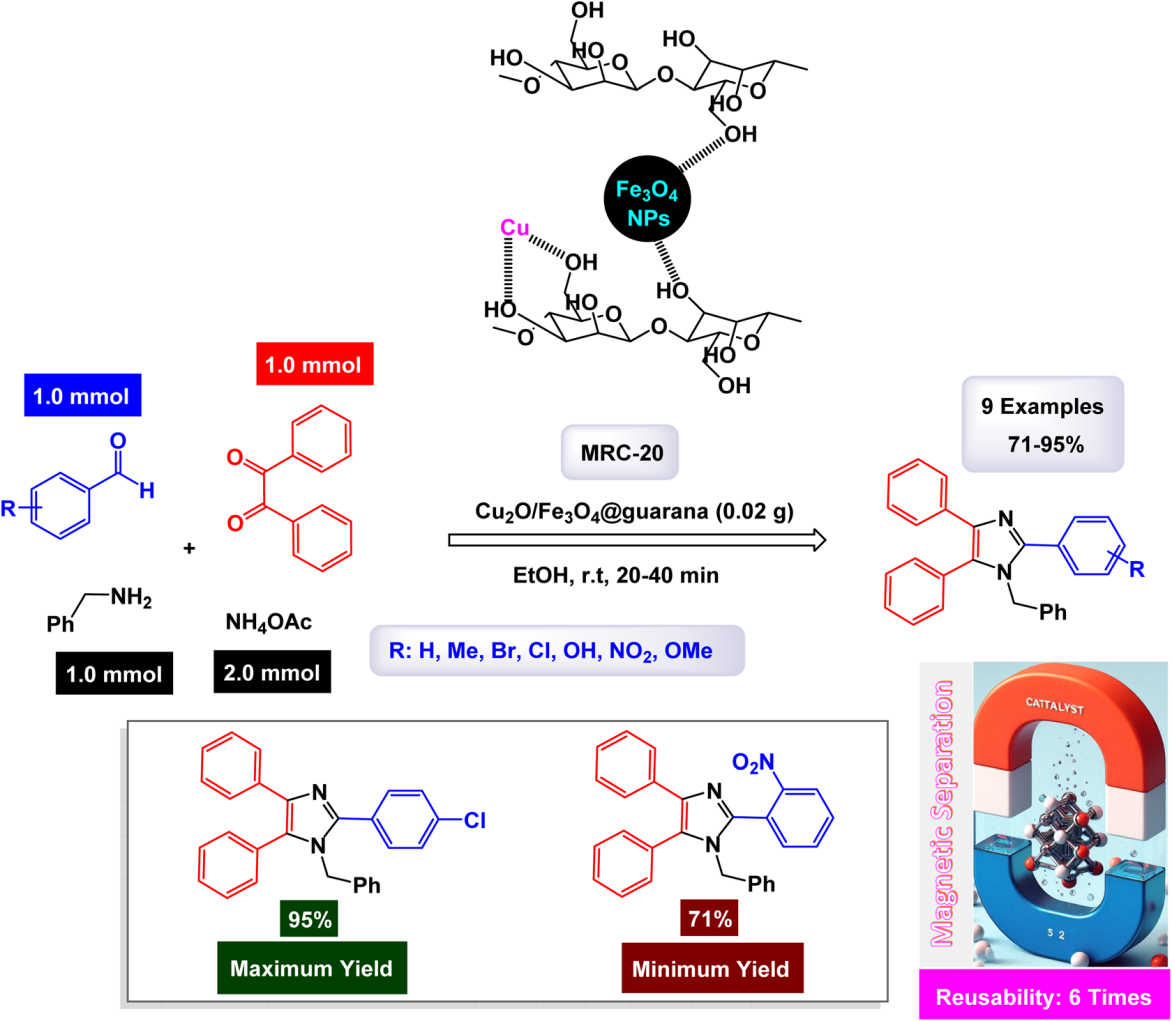
Synthesis of 1,2,4,5-tetrasubstituted imidazoles [catalysis by the Cu_2_O/Fe_3_O_4_@guarana nanomaterial].

**Scheme 33:**
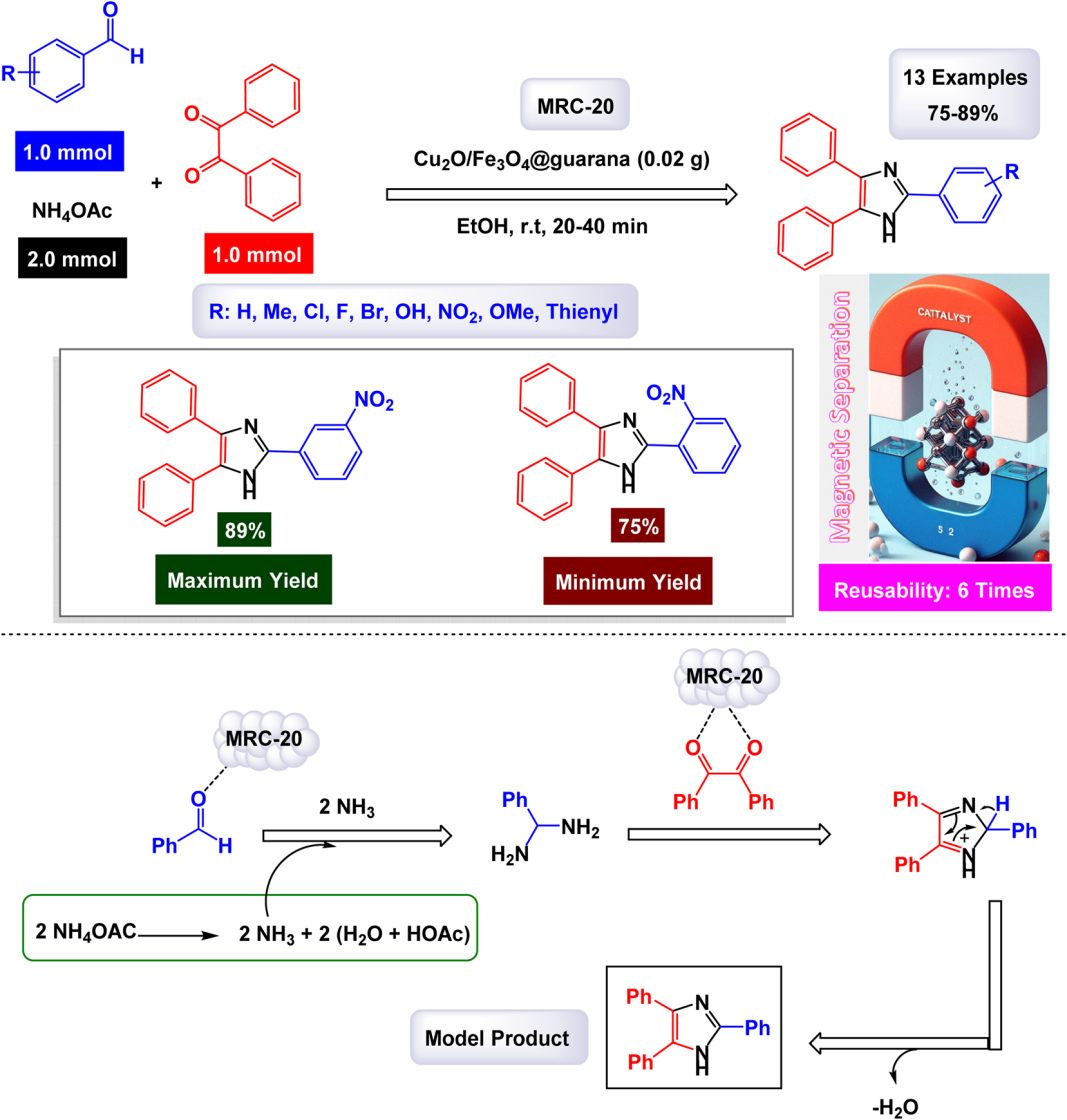
Synthesis of triaryl imidazoles [catalysis by the Cu_2_O/Fe_3_O_4_@guarana nanomaterial (MRC-20)].

A notable advancement in the synthesis of highly substituted imidazoles was achieved by the research team led by Maleki, which employed a novel catalyst known as the Cu_2_O/Fe_3_O_4_@guarana nanocomposite [MRC-20], as illustrated in Schemes 32 and 33 of their publication. The Cu_2_O/Fe_3_O_4_@guarana nanocatalyst is comprised of three key components: guar gum (derived from the natural source, guarana), magnetic iron oxide nanoparticles, and copper(i) oxide nanoparticles (Cu_2_O NPs).^141^ The structure and composition of this nanocatalyst were thoroughly validated using various spectroscopic techniques. High-resolution TEM images of the Cu_2_O/Fe_3_O_4_@guarana catalyst revealed that the average diameter of the spherical nanoparticles is approximately 40 nanometers. This finding corroborates earlier results obtained from XRD analysis, confirming the integrity of the nanostructure. According to the mechanistic pathway proposed by the authors, as depicted in Scheme 33, the copper (Cu) atoms embedded within the catalyst play a crucial role in catalyzing the reaction. They do this by activating the carbonyl group of an aldehyde, resulting in a reactive intermediate. This activated aldehyde is then transformed into a diamine through a reaction with ammonium acetate. Furthermore, the benzyl groups present in the substrate undergo activation *via* electronic interactions with the copper, facilitating their reaction with the newly formed diamine. During this reaction, a molecule of water is eliminated, culminating in the successful formation of the highly substituted imidazole product.

In the mechanism illustrated in Scheme 33 for the synthesis of triaryl imidazoles, the Cu_2_O/Fe_2_O_3_@guarana nanomaterial catalyst (MRC-20) plays a pivotal role in facilitating the reaction between ammonium acetate (NH_4_OAc) and an aromatic amine. The process begins with the activation of NH_4_OAc, where the copper component of the catalyst enhances the electrophilicity of the nitrogen atom. This activation is crucial as it makes NH_4_OAc more reactive toward nucleophilic attack by aromatic amines. As the aromatic amine approaches the activated ammonium acetate, a key intermediate is formed. The presence of MRC-20 stabilizes this intermediate through coordination interactions, effectively lowering the activation energy required for subsequent steps in the reaction pathway. Following this formation, a dehydration step occurs, wherein water is eliminated from the intermediate, yielding an imine-like species that is essential for further transformation. The next phase involves cyclization, during which another equivalent of an aromatic amine can react with the imine intermediate to form a cyclic structure. Here again, MRC-20 plays an essential role by stabilizing transition states and intermediates throughout this cyclization process. This stabilization not only promotes efficient product formation but also helps direct the reaction toward producing triaryl imidazole products.

Throughout these stages, MRC-20 enhances both reactivity and selectivity by creating an environment that minimizes side reactions while favoring the desired product formation. An additional advantage of using this nanocomposite catalyst lies in its magnetic properties; after completing a catalytic cycle, it can be easily separated from the reaction mixture using an external magnet. This feature simplifies product isolation and supports sustainability by allowing for catalyst reuse without significant loss of activity. The Cu_2_O/Fe_2_O_3_@guarana nanomaterial serves as a critical facilitator in synthesizing triaryl imidazoles by activating substrates and stabilizing key intermediates throughout various stages of the mechanism. Its ability to enhance reaction efficiency while remaining recoverable underscores its importance in advancing practical applications within organic synthesis and catalysis.

The tests conducted on the Cu_2_O/Fe_3_O_4_@guarana nanocatalyst [MRC-20] for recycling demonstrated its remarkable stability, and that the catalytic efficiency was retained with only minimal decline after being utilized six times.

**Scheme 34:**
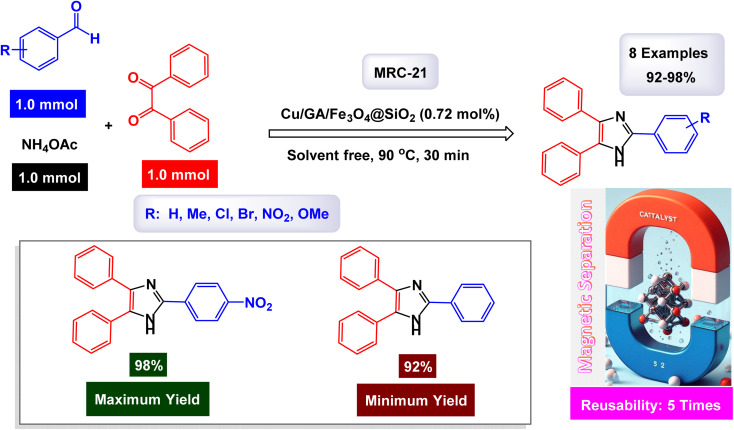
Synthesis of triaryl imidazoles using benzil as the substrate [catalysis by the Cu/GA/Fe_3_O_4_@SiO_2_ nanocomposite].

**Scheme 35:**
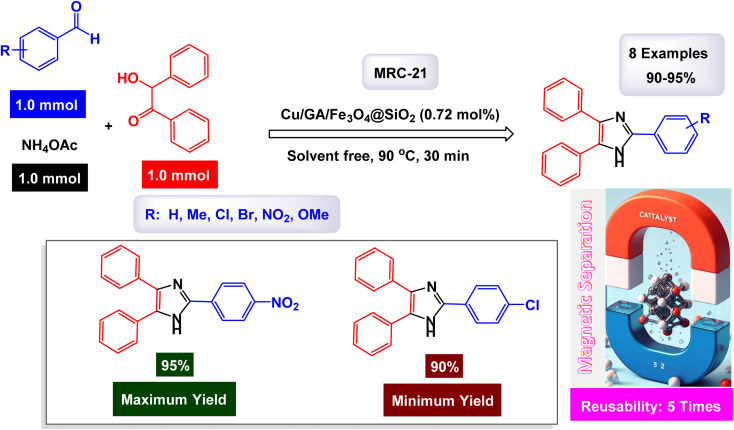
Synthesis of triaryl imidazoles using 2-hydroxy-1,2-diphenylethan-1-one as the substrate [catalysis by the Cu/GA/Fe_3_O_4_@SiO_2_ nanocomposite].

The research conducted by Shaabani and his team revealed that the Cu/GA/Fe_3_O_4_@SiO_2_ nanocomposite [MRC-21], created through the process of ultrasonic-assisted grafting of guanidine acetic acid onto modified Fe_3_O_4_@SiO_2_ core–shell nanocomposite spheres, demonstrates significant potential as an efficient catalyst.^142^ This innovative catalyst effectively facilitates the synthesis of 2,4,5-trisubstituted imidazoles from the starting materials benzil and 2-hydroxy-1,2-diphenylethan-1-one, as illustrated in Schemes 34 and 35. To validate the catalyst's stability and magnetic properties, the team employed various analytical techniques, including TGA, XRD, and VSM. The results from these analyses confirmed its robust performance. Furthermore, SEM analysis revealed that the average size of the nanocomposite particles ranges between 70 and 85 nanometers, which aligns well with data obtained from TEM observations, thereby supporting the structural integrity of the synthesized material. The tests conducted on the Cu/GA/Fe_3_O_4_@SiO_2_ nanocatalyst [MRC-21] for recycling demonstrated its remarkable stability, and that the catalytic efficiency was retained with only minimal decline after being utilized six times.

### 2.6. Catalysis by magnetic nanoparticles modified with zinc catalysts

**Scheme 36:**
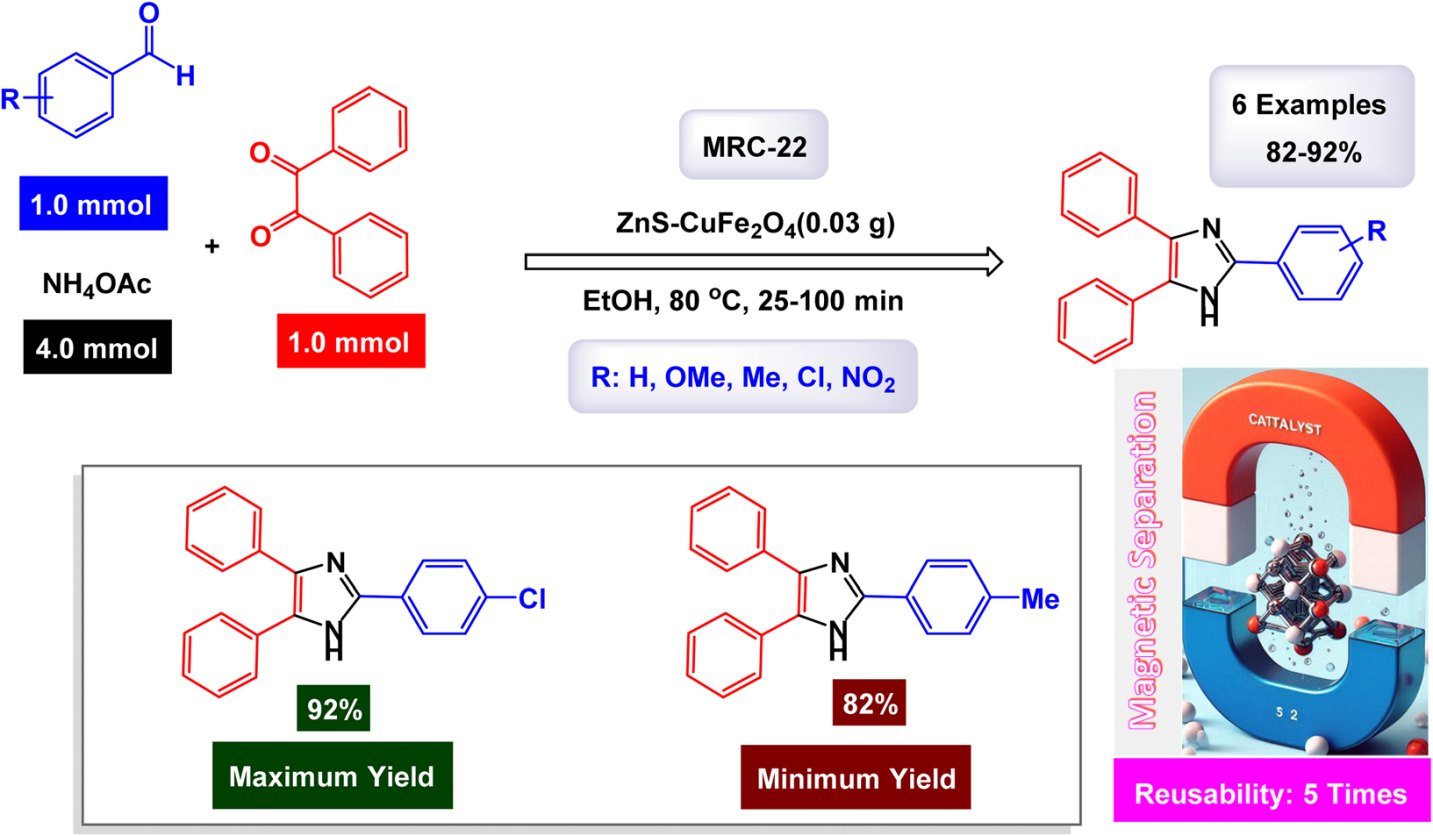
Synthesis of triaryl imidazoles [catalysis by the ZnS-CuFe_2_O_4_ nanomaterial].

Maleki and his research group successfully developed a novel nanomaterial, ZnS-CuFe_2_O_4_ [MRC-22], through a straightforward coprecipitation method.^143^ They thoroughly investigated its potential as a catalyst in the one-pot synthesis of 2,4,5-triaryl-1*H*-imidazole derivatives. This process involves the condensation of a variety of aromatic aldehydes, benzil, and ammonium acetate, showcasing the versatility and effectiveness of the nanomaterial in synthetic applications (Scheme 36). The SEM images reveal that the catalyst particles are remarkably small, with an average size of less than 20 nanometers, indicating a high surface area conducive for catalysis. The tests conducted on the ZnS-CuFe_2_O_4_ nanocatalyst [MRC-22] for recycling demonstrated its remarkable stability, and that the catalytic efficiency was retained with only minimal decline after being utilized five times.

**Scheme 37:**
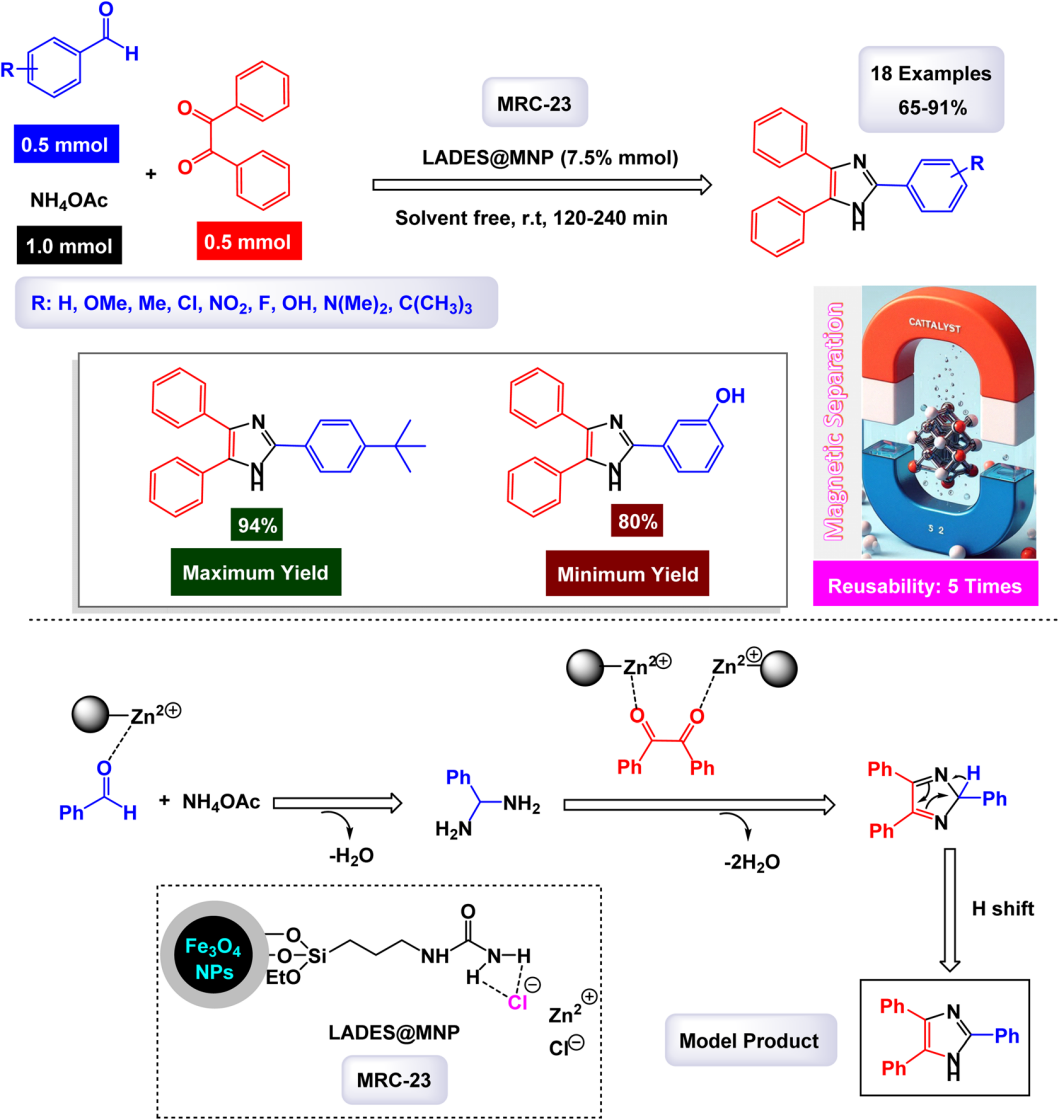
Synthesis of triaryl imidazoles [catalysis by the LADES@MNP nanomaterial].

**Scheme 38:**
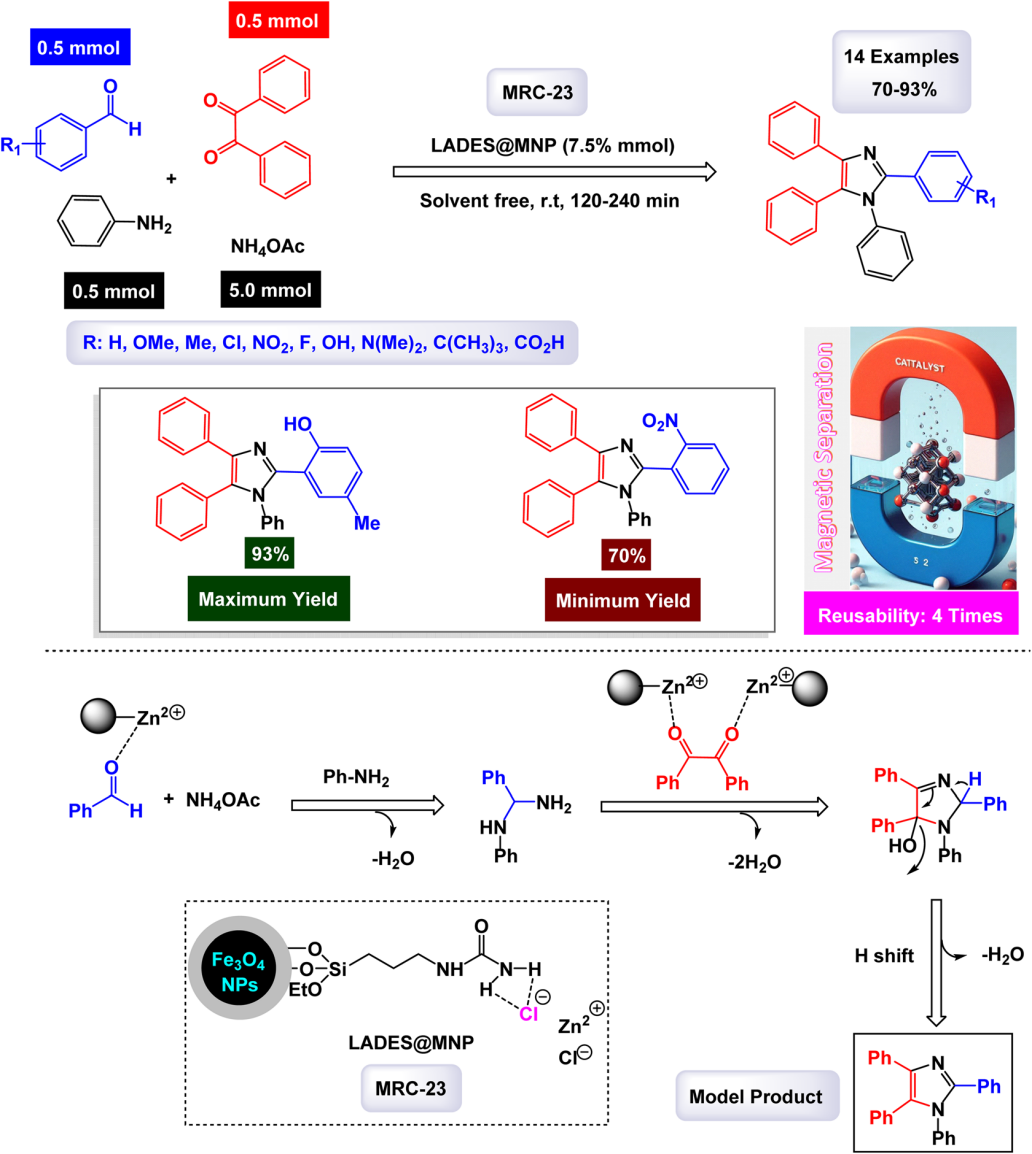
Synthesis of 1,2,4,5-tetrasubstituted imidazoles [catalysis by the LADES@MNP nanomaterial].

Hoang-Tran and his research team have made significant strides in the field of organic synthesis by developing a novel and efficient methodology for creating 2,4,5-trisubstituted and 1,2,4,5-tetrasubstituted imidazoles (Schemes 37 and 38).^144^ This innovative approach harnesses the power of a magnetically supported Lewis acidic deep eutectic solvent (LADES@MNP), which is anchored on magnetic nanoparticles. The synthesis is achieved through one-pot multicomponent reactions conducted under solvent-free sonication, allowing for a streamlined and environmentally friendly process. To optimize the conditions for imidazole synthesis, the team meticulously tested a variety of catalysts and solvents. Characterization of the magnetic nanoparticles revealed TEM images showcasing a dark core of Fe_3_O_4_, enveloped by a light grey silica shell. This design results in nanoparticles with an average diameter ranging from 15 to 25 nanometers. Additional analysis *via* SEM images indicated slight aggregation of the nanoparticles, which is likely due to interactions among the layers of the deep eutectic solvent (DES) that surround them. The SEM imagery further illustrated the nano-scale structure of the LADES@MNP, emphasizing its unique properties. The proposed reaction mechanism suggests that LADES@MNP contains Lewis acidic sites, particularly zinc atoms on its surface, which play a critical role in facilitating the reaction. These zinc sites engage with the oxygen atoms found in carbonyl groups, effectively accepting nucleophiles during the formation of imidazoles. Furthermore, the researchers posit that the inherent characteristics of the magnetic nanoparticles contribute to enhanced catalytic activity. Their nano-support allows for excellent dispersion within the reaction mixture, mimicking behavior akin to a “quasi-homogeneous” catalyst and ultimately leading to higher efficiency in the synthesis process. The tests conducted on the LADES@MNP nanocatalyst [MRC-23] for recycling demonstrated its remarkable stability, and that the catalytic efficiency was retained with only minimal decline after being utilized several times.

In the mechanism illustrated in Scheme 37 for the synthesis of triaryl imidazoles, the LADES@MNP nanomaterial catalyst, which incorporates Fe_3_O_4_ nanoparticles and zinc ions (Zn^2+^), plays a crucial role in facilitating the reaction between ammonium acetate (NH_4_OAc) and an aromatic amine. The process begins with the activation of NH_4_OAc, where the presence of Zn^2+^ enhances the electrophilicity of the nitrogen atom in NH_4_OAc. This activation is essential as it increases the reactivity of NH_4_OAc, making it more susceptible to nucleophilic attack by the aromatic amine. As the aromatic amine approaches the activated ammonium acetate, a key intermediate is formed through a nucleophilic attack on the electrophilic nitrogen. The LADES@MNP catalyst stabilizes this intermediate *via* coordination interactions, which effectively lowers the activation energy required for subsequent transformations. Following this formation, a dehydration step occurs where water is eliminated from the intermediate, leading to the generation of an imine-like species that serves as a precursor for further cyclization.

The next phase involves cyclization, during which another equivalent of an aromatic amine reacts with the imine intermediate to form a cyclic structure. The LADES@MNP catalyst continues to play an essential role during this cyclization process by stabilizing transition states and intermediates. This stabilization not only facilitates efficient product formation but also directs the reaction toward producing triaryl imidazole products with high selectivity. Moreover, throughout these stages, LADES@MNP enhances both reactivity and selectivity by creating an environment that minimizes side reactions while favoring desired product formation. An additional advantage of using this nanocomposite catalyst is its magnetic properties; after completing its catalytic cycle, it can be easily separated from the reaction mixture using an external magnet. This feature simplifies product isolation and promotes sustainability by allowing for catalyst reuse without significant loss of activity. The LADES@MNP nanomaterial serves as a vital facilitator in synthesizing triaryl imidazoles by activating substrates and stabilizing key intermediates throughout various stages of the mechanism. Its ability to enhance reaction efficiency while remaining recoverable underscores its significance in advancing practical applications within organic synthesis and catalysis.

### 2.7. Catalysis by magnetic nanoparticles modified with a silver catalyst

**Scheme 39:**
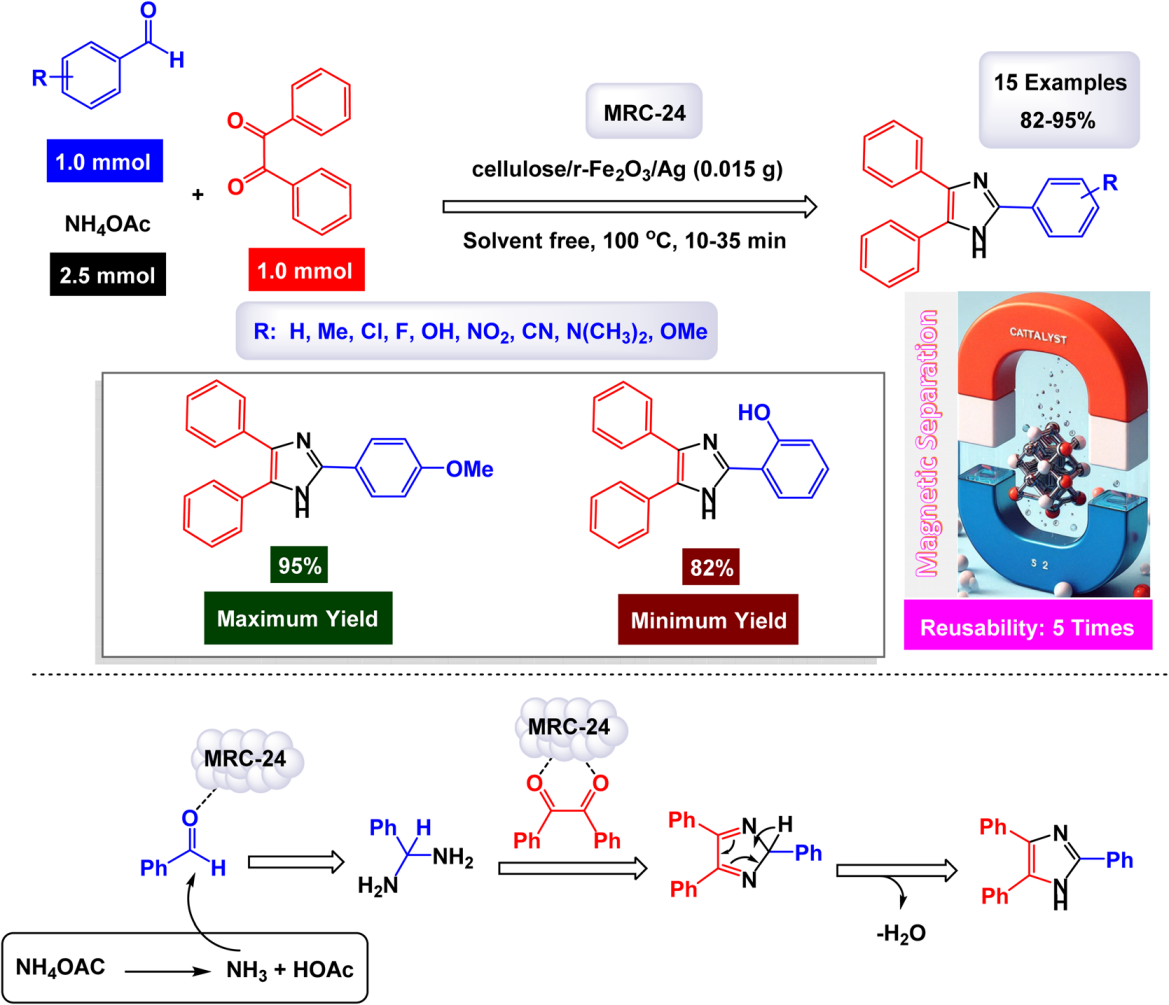
Synthesis of triaryl imidazoles from benzil [catalysis by the cellulose/γ-Fe_2_O_3_/Ag nanocomposite (MRC-24)].

**Scheme 40:**
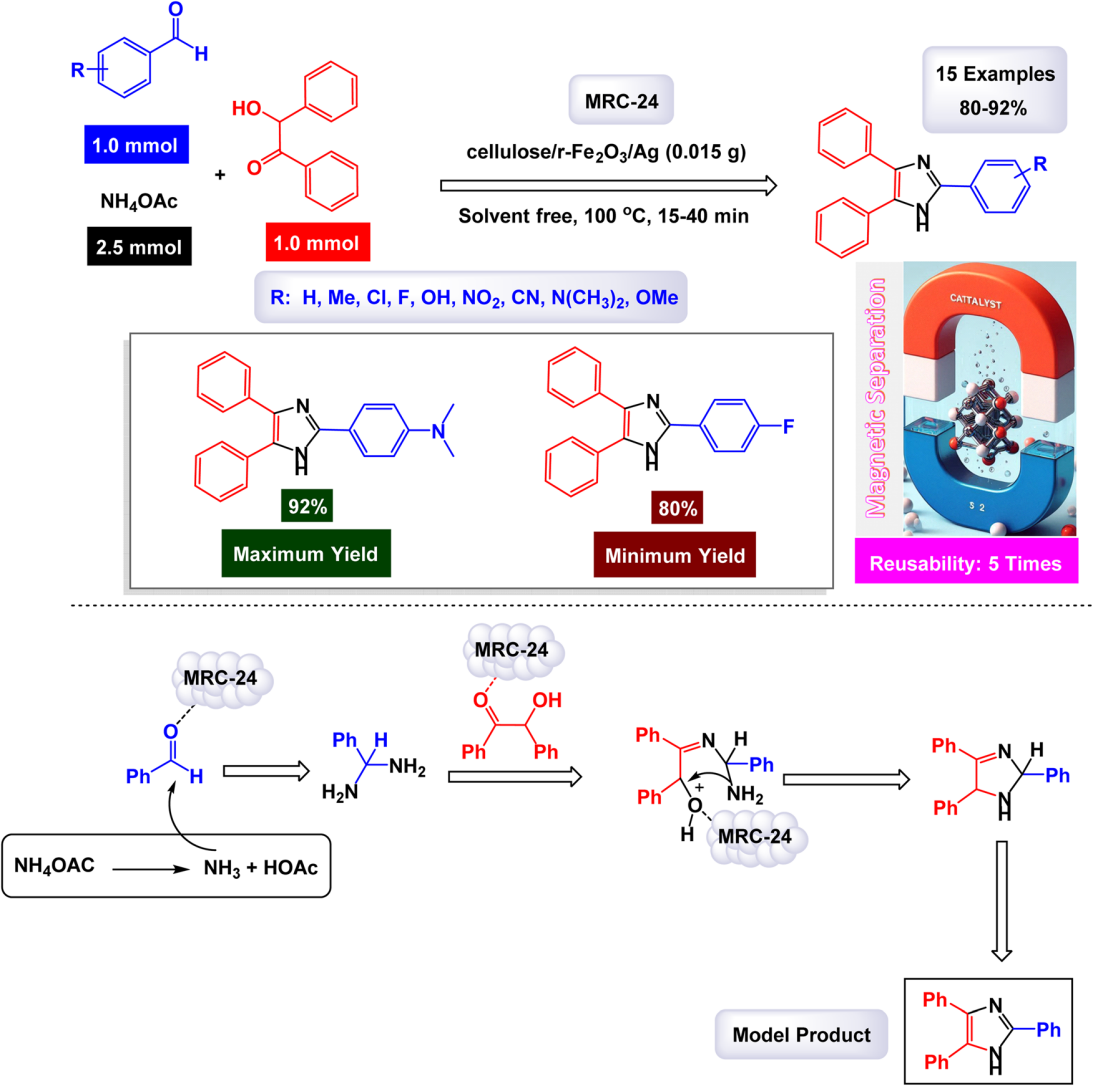
Synthesis of triaryl imidazoles from benzil [catalysis by the cellulose/γ-Fe_2_O_3_/Ag nanocomposite (MRC-24)].

Maleki and his team of researchers have pioneered an innovative catalyst that can be magnetically reused, utilizing silver catalysts in the form of a cellulose/γ-Fe_2_O_3_/Ag nanocomposite [MRC-24] for the efficient multicomponent synthesis of trisubstituted imidazoles.^145^ This cutting-edge nanocomposite was synthesized through a straightforward method and subsequently characterized using a variety of spectroscopic techniques. Analysis through SEM, TEM, and XRD revealed a narrow particle size distribution, with the majority of particles measuring approximately 15–20 nanometers in diameter. Furthermore, the catalyst exhibited remarkable magnetic properties, as well as impressive thermal stability, which were validated through VSM and TGA, respectively. The underlying mechanism of the reaction is illustrated in Schemes 39 and 40. During the process, ammonium acetate decomposes to generate ammonia and acetic acid, with the ammonia serving as a vital nitrogen source. Notably, the silver and gamma-Fe_2_O_3_ components embedded within the cellulose/γ-Fe_2_O_3_/Ag nanocomposite function as Lewis acids, facilitating the activation of carbonyl groups. This interaction significantly accelerates the production of imines, enhancing the overall efficiency of the synthesis process.

In the mechanism depicted in Scheme 39 for the synthesis of triaryl imidazoles from benzyl, the catalyst MRC-24, which consists of a cellulose/γ-Fe_2_O_3_/Ag nanocomposite, plays a pivotal role in facilitating the reaction between ammonium acetate (NH_4_OAc) and an aromatic amine. The process initiates with the activation of NH_4_OAc, where the presence of silver ions (Ag) enhances the electrophilicity of the nitrogen atom. This activation is crucial as it increases the reactivity of NH_4_OAc, making it more amenable to nucleophilic attack by the aromatic amine. As the aromatic amine approaches the activated ammonium acetate, a key intermediate forms through a nucleophilic attack on the electrophilic nitrogen. The MRC-24 catalyst stabilizes this intermediate *via* coordination interactions, effectively lowering the activation energy required for subsequent transformations. Following this formation, a dehydration step occurs during which water is eliminated from the intermediate, resulting in an imine-like species that serves as a precursor for further cyclization.

The next phase involves cyclization, wherein another equivalent of an aromatic amine reacts with this imine intermediate to form a cyclic structure. Throughout this cyclization process, MRC-24 continues to play an essential role by stabilizing transition states and intermediates. This stabilization not only facilitates efficient product formation but also directs the reaction toward producing triaryl imidazole products with high selectivity. Additionally, throughout these stages, MRC-24 enhances both reactivity and selectivity by creating an environment that minimizes side reactions while favoring desired product formation. A significant advantage of using this nanocomposite catalyst lies in its magnetic properties; after completing its catalytic cycle, it can be easily separated from the reaction mixture using an external magnet. This feature simplifies product isolation and supports sustainability by allowing for catalyst reuse without significant loss of activity. MRC-24 serves as a vital facilitator in synthesizing triaryl imidazoles by activating substrates and stabilizing key intermediates throughout various stages of the mechanism. Its ability to enhance reaction efficiency while remaining recoverable underscores its significance in advancing practical applications within organic synthesis and catalysis.

The tests conducted on the cellulose/γ-Fe_2_O_3_/Ag nanocatalyst [MRC-24] for recycling demonstrated its remarkable stability, and that the catalytic efficiency was retained with only minimal decline after being utilized six times.

All the magnetically recoverable catalysts (MRCs) listed in Table 1 demonstrated significant activity in the three-component synthesis of triaryl imidazole derivatives, with product yields generally ranging from 29% to 98%. Among them, the catalyst Fe_3_O_4_@Diamine-CuI (entry 16, Table 1) achieved the highest yields (up to 98%) under mild reaction conditions. In terms of recyclability, MnFe_2_O_4_ (entry 3) and Fe_3_O_4_@Diamine-CuI (entry 16) exhibited the best reusability, retaining activity through seven consecutive cycles. Notably, Fe_3_O_4_ (entry 1) and LADES@MNP (entry 20) were effective at room temperature, representing energy-efficient alternatives. Among all systems, MNPs-SiO_2_-urea (entry 4) stood out by delivering high yields using only 0.0088 g of catalyst, highlighting its high catalytic efficiency at low loading. Several catalysts also performed well under solvent-free conditions, including Ni_0_._5_Zn_0_._5_Fe_2_O_4_ (entry 2), [P_4_-VP]-Fe_3_O_4_ (entry 5), and Fe_3_O_4_@SiO_2_-imid-PMA^*n*^ (entry 11), supporting environmentally friendly practices. On the other hand, ethanol was the most frequently used solvent across active systems such as Fe_3_O_4_ (entry 1), Fe_3_O_4_@CS (entry 9), and MPBNPs (entry 14), offering a good balance between green chemistry and efficiency. The shortest reaction time (as low as 5 minutes) was reported with Fe_3_O_4_@SiO_2_-imid-PMA^*n*^ (entry 11). Additionally, most catalysts demonstrated a broad substrate scope, effectively tolerating various electron-donating and electron-withdrawing groups, as well as heterocyclic aldehydes, emphasizing the general applicability of these MRC-based catalytic systems.

**Table 1:** Comparative overview of MRC-catalyzed three-component one-pot syntheses of triaryl imidazole derivatives

Entry	Catalyst	Catalyst loading	Solvent	*T* (°C)	Time (min)	Yield%	Recyclability	Substrate scope
1	Fe_3_O_4_	5 mol%	EtOH	r.t.	25–180	88–97%	5	Broad
2	Ni_0.5_Zn_0.5_Fe_2_O_4_	5 mol%	Solvent free	80 °C	10–35	65–91%	5	Broad
3	MnFe_2_O_4_	25 mg	H_2_O/EtOH	80 °C	40–120	83–94%	7	Narrow
4	MNPs-SiO_2_-urea	0.0088 g	EtOH	Reflux		57–98%	6	Broad
5	[P_4_-VP]-Fe_3_O_4_	100 mg	Solvent free	100 °C	35–240	68–99%	6	Broad
6	Fe_3_O_4_@FU	12 mg	EtOH	Reflux	12–30	80–96%	6	Narrow
7	FeCeO_*x*_@g-C_3_N_4_	0.4 mol%	EtOH	80 °C	90–100	29–98%	4	Broad
8	H_3_PW_12_O_40_-amino-functionalized CdFe_12_O_19_@SiO_2_	6 mg	Solvent free	110 °C	25–45	82–94%	6	Narrow
9	Fe_3_O_4_@CS	0.05 g	EtOH	Reflux	60–210	90–98%	6	Broad
10	Fe_3_O_4_@SiO_2_-EPIM	0.02 g	PEG	100 °C	20–85	50–98%	6	Broad
11	Fe_3_O_4_@SiO_2_-imid-PMA^*n*^	0.03 g	Solvent free	110 °C	5–20	80–97%	6	Broad
12	γ-Fe_2_O_3_-SO_3_H	10 mol%	Solvent free	100 °C	40–70	90–98%	4	Broad
13	SiO_2_/Fe_3_O_4_/SO_3_H	0.01 g	Solvent free	110 °C	40–100	85–97%	5	Narrow
14	MPBNPs	10 mg	EtOH	Reflux	42–70	80–96%	6	Broad
15	SA-MNPs	0.012 mmol%	Solvent free	100 °C	15–35	75–88%	6	Broad
16	Fe_3_O_4_@Diamine-CuI	20 mg	EtOH	Reflux	20–40	84–98%	8	Broad
17	Fe_3_O_4_-PEG-Cu	10 mol%	Solvent free	110 °C	20–45	88–89%	6	Broad
18	Cu/GA/Fe_3_O_4_@SiO_2_	0.72 mol%	Solvent free	90 °C	30	92–98%	5	Narrow
19	ZnS-CuFe_2_O_4_	0.03 g	EtOH	80 °C	25–100	82–89%	5	Narrow
20	LADES@MNP	7.5 mmol%	Solvent free	r.t.	120–240	65–91%	5	Broad
21	Cellulose/γ-Fe_2_O_3_/Ag	0.015 g	Solvent free	100 °C	10–35	82–95	5	Broad

As shown in Table 2, the performance of magnetic nanocatalysts was compared with that of various other catalytic systems. While these systems have demonstrated acceptable yields (ranging from 10% to 97%), several significant drawbacks limit their practical applicability when compared to magnetic catalysts (Table 1). Notably, some methods (*e.g.*, entry 1) require exceptionally long reaction times of up to 720 minutes, which is impractical for large-scale synthesis. Additionally, many of the listed protocols depend on ultrasound irradiation (entry 6) or elevated temperatures under solvent-free conditions (entries 3 and 5), which may not be energy-efficient or universally applicable. Furthermore, the high catalyst loading (*e.g.*, 25 mol%) in entry 6 and 15–20 mol% in several others, and the use of non-recoverable homogeneous systems such as l-proline (entry 10) or CAN (entry 9) make catalyst recycling challenging due to difficult separation processes like filtration or centrifugation, which often lead to catalyst loss or degradation. These limitations significantly undermine the sustainability and cost-efficiency of such catalytic systems, highlighting the clear advantages of magnetically recoverable nanocatalysts that enable easy magnetic separation, minimal catalyst loading, and shorter reaction times under eco-friendly conditions.

**Table 2:** Comparison of non-magnetic catalytic systems for the synthesis of highly substituted imidazoles in terms of yield and reaction conditions

Entry	Catalyst	Conditions	Time (min)	Yield%	Ref.
1	Yb(OPf)_3_ (0.004 mmol%)	Perflurodecalin/80 °C	360–720	10–97%	146
2	Montmorilonite K10 (25 mg)	EtOH/reflux	90–120	73–75%	147
3	SBSSA (0.002 g)	Solvent free/130 °C	30–120	84–95%	148
4	DBSA (20 mol%)	H_2_O/reflux	240	50–87%	149
5	poly(AMPS-*co*-AA) (0.03 g)	Solvent free/110 °C	25–35	80–90%	150
6	Zr(acac)4 (25 mol%)	EtOH/Ultrasound irradiation/r.t.	25–85	81–97%	151
7	Polymer-ZnCl_2_ (15 mol%)	EtOH/78 °C	240	84–97%	152
8	SSA (0.5 g)	H_2_O/reflux	240–280	59–81%	153
9	CAN (5 mol%)	EtOH/reflux/under aerobic oxidation conditions	180–360	65–85%	154
10	l-Proline (15 mol%)	MeOH/60 °C	540–780	69–92%	155

## 3. Challenges and opportunities

Despite the significant progress made in the design and application of magnetic nanocatalysts for the synthesis of highly substituted imidazoles, several key challenges remain. One of the major bottlenecks is the limited understanding of the exact nature of the active catalytic sites and the mechanistic pathways involved. Many proposed mechanisms are schematic and lack experimental validation through spectroscopic techniques or theoretical support, such as DFT studies. This hampers rational catalyst design and optimization.

Another challenge lies in the scalability and industrial translation of these catalytic systems. While many catalysts show excellent performance under laboratory conditions, their activity, selectivity, and recyclability often decline when applied to large-scale processes. Additionally, issues such as nanoparticle agglomeration, loss of magnetic properties over cycles, and catalyst leaching require further investigation and mitigation. Moreover, the high cost associated with certain metal-stabilizing ligands adds another layer of economic constraint, potentially limiting the commercial viability of these catalytic systems in industrial applications.

Opportunities for advancement lie in integrating magnetic nanocatalysts with modern technologies. For instance, incorporating them into continuous flow reactors could enable scalable, waste-minimized processes. Furthermore, employing machine learning and high-throughput screening techniques can accelerate the discovery of optimized catalyst compositions and surface modifications. Hybrid systems that combine magnetic nanocatalysts with photocatalysis or electrocatalysis also represent promising directions for achieving energy-efficient, sustainable synthesis.

## 4. Conclusion and outlook

Magnetic nanocatalysts have emerged as a highly effective and versatile platform in the green synthesis of highly substituted imidazoles, as thoroughly reviewed in this study. These catalysts consistently provide high yields across diverse substrates and reaction conditions, including solvent-free systems, room temperature, and environmentally friendly conditions, while enabling facile magnetic recovery and recyclability. The mechanistic insights discussed, such as the pivotal role of Lewis acidic sites, quaternary ammonium cations, and metal centers (*e.g.*, Cu^+^ and Fe^3+^) in activating carbonyl groups and facilitating intermediate formation, further underscore their catalytic efficiency and specificity. Nonetheless, several challenges remain critical for advancing the field. Catalyst deactivation and gradual loss of activity after multiple reaction cycles, often due to nanoparticle agglomeration, leaching of active sites, or structural degradation, limit their practical application on a larger scale. The variability in substrate scope and sensitivity to reaction parameters also highlights the need for more robust and universally applicable catalytic systems.

Future research should therefore prioritize a comprehensive understanding of catalyst structure–activity relationships through combined experimental and theoretical approaches, including advanced spectroscopic characterization and density functional theory (DFT) studies. Enhancing catalyst stability *via* surface modification, core–shell architectures, or incorporation of stabilizing ligands could mitigate activity loss. Furthermore, integrating magnetic nanocatalysts into continuous flow reactors and coupling them with green solvent technologies offers promising routes for scalability and sustainability.

In conclusion, while magnetic nanocatalysts present a transformative approach for the eco-friendly synthesis of substituted imidazoles, overcoming the challenges related to catalyst longevity and process integration will be essential. The ongoing interdisciplinary efforts combining materials science, catalysis, and computational modeling are expected to yield next-generation catalysts with superior performance, selectivity, and durability, thereby consolidating their role in sustainable organic synthesis for both academic research and industrial applications.

## Author contributions

Mostofa Kazemi was primarily responsible for supervising the study, as well as its conceptualization and design. Ramin Javahershenas played a key role in overseeing the project, providing strategic guidance and valuable insights throughout the research process. Mosstafa Kazemi defined the main objectives, designed the study methodology, and managed the project timeline to ensure smooth progression. Jayanti Makasana, Suhas Ballal, and Munther Kadheem contributed significantly by conducting investigations and literature searches. The drafting of the manuscript was led by Abhayveer Singh, Kattela Chennakesavulu, and Kamal Kant Joshi, with Jayanti Makasana, Kattela Chennakesavulu, and Suhas Ballal responsible for analyzing and presenting the findings through clear figures. Mosstafa Kazemi and Ramin Javahershenas conceived the core ideas, analyzed the data, and authored the initial draft; they also participated in reviewing, editing, and proofreading the manuscript. All authors engaged in discussions and contributed to editing the final version, which was thoroughly reviewed, read, and approved by each contributor.

## Conflicts of interest

The authors declare no conflict of interest.

## Abbreviations

MRC: Magnetically recoverable catalyst
TEM: Transmission electron microscopy
SEM: Scanning electron microscopy
FT-IR: Fourier-transform infrared spectroscopy
ILs: Ionic liquids
PFA: Poly formaldehyde aniline
MNP: Magnetic nanoparticle
LAIL: Imidazolium chlorozincate(ii) ionic liquid
DMSO: Dimethyl sulfoxide
TGA: Thermogravimetric analysis
VSM: Vibrating sample magnetometry
XRD: X-ray diffraction
EDTA: Ethylenediaminetetraacetic acid
DESs: Deep eutectic solvents
ChCl/urea: Choline chloride/urea
DMF: Dimethylformamide
TBAB: Tetrabutylammonium bromide
FE-SEM: Field emission scanning electron microscopy
BTP: (2-Benzothiazolyl)pyridine
UV: Ultraviolet
PEG: Polyethylene glycol
AAS: Atomic absorption spectrometry
nm: Nanometer
SMNP@GLP: Silica magnetic nanoparticles glucose palladium
PFMNPs: Fe_3_O_4_@SiO_2_@PPh_2_
EPIM: Epichlorohydrin and 1-methyl-imidazole
MPBNPs: Magnetic polyborate nanoparticles
SA-MNPs: Sulfamic acid-functionalized magnetic nanoparticles
[P_4_-VP]-Fe_3_O_4_ NPs: Poly(4-vinylpyridine) supported Fe_3_O_4_ nanoparticles
FU: Fucoidan (a fucose-rich sulfated polysaccharide extracted from brown algae *Fucus vesiculosus*)
US: Ultrasonic irradiation
LADES: Lewis acidic deep eutectic solvent

## Data Availability

No primary research results, software or code have been included and no new data were generated or analyzed as part of this review.
